# Supplementary material for: The Extracellular Surface of the GLP-1 Receptor Is a Molecular Trigger for Biased Agonism
Source: Cell. 2016 Jun 16;165(7):1632–43. doi: 10.1016/j.cell.2016.05.023 (PMC4912689; doi:10.1016/j.cell.2016.05.023)
Supplement: Document S1. Supplemental Experimental Procedures and Tables S1–S6 [file mmc1.pdf]

Cell, Volume *165*

Supplemental Information

The Extracellular Surface of the GLP-1 Receptor

Is a Molecular Trigger for Biased Agonism

Denise Wootten, Christopher A. Reynolds, Kevin J. Smith, Juan C. Mobarec, Cassandra Koole, Emilia E. Savage, Kavita Pabreja, John Simms, Rohan Sridhar, Sebastian G.B. Furness, Mengjie Liu, Philip E. Thompson, Laurence J. Miller, Arthur Christopoulos, and Patrick M. Sexton

Supporting Information

Supplemental Experimental Procedures

Materials. GLP-1(7-36)NH_2, exendin-4 and oxyntomodulin were purchased from Mimotopes (Victoria, Australia). Dulbecco's Modified Eagle's Medium (DMEM) and Fluo-4 AM were purchased from Invitrogen (Carlsbad, CA, USA). Foetal bovine serum (FBS) was purchased from Thermo Electron Corporation (Melbourne, VIC, Australia). AlphaScreen reagents, 384-well opti and proxiplates were purchased from PerkinElmer Life and Analytical Sciences (Waltham, MA, USA). SureFire^{TM} ERK1/2 reagents were obtained from TGR Biosciences (Adelaide, SA, Australia). HRTF® insulin kit was from Cisbio. AlexaFluor® 488 annexin V conjugate (A13201) was from Molecular Probes. BrdU Cell Proliferation ELISA (chemiluminescent) kit was from Roche. N^{α}-Fmoc protected amino acids were purchased from Auspep, Chemimpex and Mimotopes. Rink amide resin (0.53 meq/g, 100-200 mesh), 2-chlorotrityl chloride resin (1.12 meq/g, 200-400 mesh) and HCTU were obtained from Chemimpex. TFA was purchased from Alfa Aesar. All other reagents were purchased from Sigma-Aldrich (St. Louis, MO, USA) or BDH Merck (Melbourne, Vic, Australia) and were of an analytical grade.

Peptide synthesis. Linear peptide chains (0.1 mmol scale) were synthesised on Rink amide resin [Q_3-GLP-1(7-36)NH_2] or 2-chlorotrityl chloride resin [E_3-oxyntomodulin] using a 3-channel serial automated peptide synthesiser ("PS3", Protein Technologies Inc.), which adopted standard Fmoc-based solid phase synthesis strategy. Fmoc deprotection was performed by 20% v/v piperidine in DMF for 2×5 min. Fmoc protected amino acids (3 *eq.*) were coupled using DMF as solvent, and DIPEA in DMF (7% v/v) with HCTU (3 *eq.*) as the activating agent for 50 min. Protected peptidyl-resins were cleaved by treating with TFA-TIPS-DMB (92.5%:2.5%:5%, for Rink amide resin) or TFA-TIPS (95%:5%, for 2-chlorotrityl chloride resin) for 2 h. The cleavage mixture was filtered, concentrated by a stream of N_2, precipitated in cold Et_2O and centrifuged at 3000 rpm for 5 min. The crude product was dissolved in water-acetonitrile mixture (50%:50%) and lyophilised, then purified by semi-preparative RP-HPLC. Peptides were purified on a Phenomenex Luna C-8 column (100Å, 10μm, 250×21.2mm) utilising a Waters 600 semi-preparative RP-HPLC that incorporates a Waters 486 UV detector. The wavelength was set at 230 nm. The eluting profile was a linear gradient of 0-80% acetonitrile in water buffered with 0.1% TFA over 60 min at 10 ml/min. ESI-MS was conducted using a Shimadzu LCMS2020 instrument, incorporating a Phenomenex Luna C-8 column (100Å, 3μm, 100×2.00mm). This system used 0.05% TFA in MilliQ water as the aqueous buffer, and 0.05% TFA in acetonitrile as the organic buffer. The eluting profile was a linear gradient of 0-60% acetonitrile in water over 10 minutes at 0.2 ml/min.

Receptor mutagenesis. To study the influence of ECL amino acids, and R190 on receptor function, the desired mutations were introduced to an N-terminally double c-myc labeled wild-type human GLP-1R in the pEF5/FRT/V5-DEST destination vector (Invitrogen); this receptor had equivalent pharmacology to the untagged human GLP-1R. Mutagenesis was carried out using oligonucleotides for site-directed mutagenesis purchased from GeneWorks (HindMarsh, SA, Australia) and the QuikChange^{TM} site-directed mutagenesis kit (Stratagene) and confirmed by automated sequencing (AGRF, Vic, Australia).

Cell culture: ***Stable transfections***; Wild-type and mutant human GLP-1Rs were isogenically integrated into FlpIn-Chinese hamster ovary (FlpInCHO) cells (Invitrogen) and selection of receptor-expressing cells accomplished by treatment with 600 μg ml^{-1} hygromycin-B as previously described (1). ***ChoFlpIn cell culture;*** All Transfected and parental FlpInCHO cells were maintained in DMEM supplemented with 10% heat-inactivated FBS and incubated in a humidified environment at 37°C in 5% CO_2. ***Ins-1 832/3 cell culture;*** Cells were maintained in RPMI 1640, supplemented with 10% v/v FBS, 1mM sodium pyruvate, 50μM 2-mercaptoethanol, 10mM HEPES, penicillin/streptomycin (50μg/mL) and 11mM glucose at 37°C in 5% CO_2.

Radioligand binding assay. FlpInCHO GLP-1R cells were seeded at a density of 3 x 10^4 cells/well into 96-well culture plates and incubated overnight at 37°C in 5% CO_2, and radioligand binding carried out as previously described (Koole et al., 2012). For each cell line in all experiments, total binding was defined by 0.05 nM ^{125}I-exendin-4(9-39) alone, and nonspecific binding was defined by 1 μM exendin-4(9-39). For analysis, data are normalised to the specific binding for each individual experiment.

Cell surface receptor expression. FlpInCHO WT and mutant human GLP-1R cells, with receptor DNA previously incorporated with an N-terminal double c-myc epitope label, were seeded at a density of 25 x 10^4 cells/well into 24-well culture plates and incubated overnight at 37°C in 5% CO_2, washed three times in 1 x PBS and fixed with 3.7% paraformaldehyde (PFA) at 4°C for 15 min. Cell surface receptor detection was then performed as previously described (Koole et al., 2012). Data were normalized to the basal fluorescence detected in FlpInCHO parental cells. Specific ^{125}I-exendin-4(9-39) binding at each receptor mutant, as identification of functional receptors at the cell surface, was also determined (corrected for nonspecific binding using 1 μM exendin-4(9-39)).

cAMP accumulation assay. FlpInCHO WT and mutant human GLP-1R cells were seeded at a density of 3 x 10^4 cells/well into 96-well culture plates and incubated overnight at 37°C in 5% CO_2. Peptide mediated cAMP accumulation assays were performed using the PerkinElmer AlphaScreen^{TM} kit as previously described (Koole et al., 2010). For Ins-1 832/3 cells were seeded at a density of 3 x 10^4 cells/well into laminin coated 96-well cell culture plates and were incubated for 48 h at 37°C in 5% CO_2. Cells were washed with 1X EBSS supplemented with 1.8 mM $CaCl_2$ and 2.8 mM glucose, pH 7.4 and media was replaced with RPMI containing 2.8mM glucose for 90 mins. Cells were washed and further incubated for 30 min in 135 μL of EBSS containing 1.8 mM $CaCl_2$, 2.8 mM glucose, 0.1% (w/v) BSA and 1 mM 3-isobutyl-1-methylxanthine (IBMX), pH 7.4, at 37°C in 5% CO_2. A. Cells were stimulated with the relevant concentrations of peptide for 30 min at 37°C in 5% CO_2 in the presence of low (2.8mM) or high (11mM) glucose. The reaction was terminated by rapid removal of the ligand containing buffer and addition of 100 μL of ice-cold 100% ethanol. cAMP was detected usinf the PerkinElmer AlphaScreen^{TM} kit as previously described for ChoFlpIn cells (Koole et al., 2010). All values were converted to concentration of cAMP using a cAMP standard curve performed in parallel, and data were subsequently normalized to the response of 100 μM forskolin.

ERK1/2 phosphorylation assay. FlpInCHO GLP-1R cells were seeded at a density of 3 x 10^4 cells/well into 96-well culture plates and incubated overnight at 37°C in 5% CO_2. Receptor-mediated pERK1/2 was determined using the AlphaScreen^{TM} ERK1/2 SureFire^{TM} protocol as previously described (Koole et al., 2010). For Ins-1 832/3 cells were seeded at a density of 3 x 10^4 cells/well into laminin coated 96-well cell culture plates and were incubated for 48 h at 37°C in 5% CO_2. Cells were washed with 1X EBSS supplemented with 1.8 mM $CaCl_2$ and 2.8 mM glucose, pH 7.4 and incubated for 5h in RPMI 1640 containing 2.8 mM glucose. Media was replaced with 135 μL 1X EBSS supplemented with 1.8 mM $CaCl_2$, 0.1% w/v BSA and 2.8 mM glucose, pH 7.4 and cells incubate for a further 1h. Cells were stimulated with the relevant concentrations of peptide ligand in either low (2.8mM) or high (11mM) glucose. Agonist stimulation of cells was terminated by the removal of buffer and the addition of 50 μL of SureFire lysis buffer to each well. Lysates were stored at -20°C. pERK1/2 was determined using the AlphaScreen^{TM} ERK1/2 SureFire^{TM} detection as previously described for ChoFlpIn cells (Koole et al., 2010).

Initial pERK1/2 time course experiments were performed over 1 h to determine the time at which agonist-mediated pERK1/2 was maximal for both the ChoFlpIn and Ins-1 832/3 cells. Subsequent experiments were then performed at the peak time required to generate a maximal pERK1/2 response. Note: None of the mutant receptors significantly altered the kinetic profile of the pERK1/2 response. Data were normalized to the maximal response elicited by 10% FBS determined at 6 min in both ChoFlpIn and Ins1-832 cells (peak FBS response).

Intracellular Ca^{2+} mobilisation assay. FlpInCHO GLP-1R cells were seeded at a density of 3 x 10^4 cells/well into 96-well culture plates and incubated overnight at 37°C in 5% CO_2, and receptor-mediated intracellular Ca^{2+} mobilisation determined as previously described (Koole et al., 2010). Fluorescence was determined immediately after ligand addition, with an excitation wavelength set to 485 nm and an emission wavelength set to 520 nm, and readings taken every 1.36 s for 120 s. Peak magnitude was calculated using five-point smoothing, followed by correction against basal fluorescence. The peak value was used to create concentration-response curves. None of the mutant receptors altered the kinetic profile of the calcium response. Data were normalized to the maximal response elicited by 100 μM ATP.

Insulin secretion. Ins-1 832/3 cells were seeded at 3x10^4 cells/well on laminin coated 96-well plates using RPMI 1640 complete media and incubated at 37°C with 5% CO_2 for 24h. Cells were washed and media replaced with RPMI-1640 supplemented with 2.8mM glucose and 2.5 % FBS and incubated for a further 24h. Prior to assay, the cells were washed twice with 1X EBSS supplemented with 2.8 mM

glucose, 1.8 mM $CaCl_2$ and 0.1% w/v BSA, pH 7.4. Peptide ligands were prepared at the relevant concentrations in 1X EBSS supplemented 1.8 mM $CaCl_2$ and 0.1% w/v BSA, pH 7.4 and with with either 2.8mM and 11 mM glucose and added directly to the cells after washing. After 2h, the supernatants were collected and insulin levels were detected using Cisbio HRTF® insulin kit following the manufacturers recommendations.

Apoptosis assay. Ins-1 832/3 cells plated were plated on to 6-well plates at a density of 5×10^5 cells/well using pre-warmed RPMI 1640 complete media and incubate at 37°C in 5% CO_2 for 48h. Media was replaced with RPMI 1640 supplemented with 2.8 mM and 2.5% FBS and incubated for a further 24h. Cells were washed twice with 1X EBSS supplemented with 2.8 mM glucose and 1.8 mM $CaCl_2$, pH 7.4. Subsequently, peptides prepared at the relevant concentrations in the presence and absence of 100 nM staurosporine in RPMI 1640 complete media with either 2.8mM or 11mM glucose were added to the cells (2mL). Cells were incubated for a further 24 h at 37°C with 5% CO_2. Following incubation supernatant was collected and remaining cells were harvested and pelleted at 350g for 3 min. The supernatant was discarded and cells were resuspended in HBSS, pH 7.4. Apoptotic cells were identified using AlexaFluor® 488 annexin V conjugate (1:10 dilution, A13201, Molecular Probes ®) and PI (1mg/mL in water). Apoptotic cells (annexin and PI positive) were identified using a BD FACS Canto™ II flow cytometer measuring the fluorescence at 530 nm / 30 nm (band pass) and 585 nm / 42 nm using 488 nm excitation.

Proliferation assay. Ins-1 832/3 cells were seeded at 1.2×10^4 cells/well in laminin coated, 96-well, clear bottomed, black view plates in pre-warmed culture media and incubated for 24h at 37°C in 5% CO_2. Media was replaced with RPMI 1640 supplemented with 2.8mM glucose and incubated for a further 12h. Cells were washed and media replaced with 200µL RPMI 1640 containing 1% FBS, 0.2% BSA, 2mM sodium pyruvate, 100 µM β-mercaptoethanol, 20 mM HEPES and either low glucose (2.8mM) or high glucose (11mM), containing the relevant concentrations of peptide ligands. Media with 10% FBS was used at a positive control. Plates were incubated at 37°C in 5% CO_2 for 24h. Media was removed from the wells and proliferation was assessed by BrdU incorporation using the Cell Proliferation ELISA, BrdU (chemiluminescent) kit from Roche, following the manufacturers instructions. Luminescence was detected using the LumiSTAR Omega instrument (BMG LABTECH GmbH, Ortenberg, Germany).

Molecular modelling. An active model of the GLP1 human receptor (residues 27-421) containing both GLP-1(7-36)NH_2 and the C-terminal peptide of Gs was constructed from a number of overlapping structural templates using Modeller 9.15 (Eswar et al., 2007). These templates included the glucagon receptor TM X-ray structure (pdb code 4L6R (Siu et al., 2013)), the extracellular domain containing GLP-1(10-35) (pdb code 3IOL (Underwood et al., 2010)) and two fragments taken from the β_2-adrenergic receptor:G-protein active complex (pdb code 3SN6 (Rasmussen et al., 2011)), namely helices 5 and 6 (the biggest structural difference reside in TM6) and an intracellular portion containing the lower part of the TM domain. Both fragments contained the Gs C-terminal peptide (R373-L394); these fragments were mutated to their GLP-1R equivalent (Taddese et al., 2014) using Modeller. In addition, the CRF-1R X-ray structure (pdb code 4K5Y (Hollenstein et al., 2013)) was used to generate an ECL1 helix-containing template as 4L6R lacks a full ECL1. The NMR structure of an active, conformationally-constrained version of GLP-1(7-17) (pdb code 2N0I (Hoang et al., 2015)), with activity similar to GLP-1(7-36)NH_2 was used to model the N-terminal part of the peptide.

All 15 members of the 2N0I ensemble were mutated to GLP-1(7-17)-NH_2 and minimized using PLOP (Jacobson et al., 2004). They were then docked using GLIDE SP peptide (Friesner et al., 2004; Tubert-Brohman et al., 2013) and the recent OPLS3 force field, with the backbone conformation held rigid, to reflect the biologically active conformation, to a GLP-1R model derived from the glucagon X-ray structure (Siu et al., 2013) that had been primed by Modeller (Eswar et al., 2007) to bind GLP-1(7-36)NH_2. The top scoring pose was for model 7, which belongs to the second cluster of the NMR ensemble, as determined by NMRclust (Kelley et al., 1997; Kelley and Sutcliffe, 1997), and was the only pose to form two good salt-bridge interactions (Table S5). The alpha helical residues T13-S17 of GLP-1(7-17) were structurally aligned to the corresponding residues of GLP-1(10-35) of the 3IOL structure using VMD (Humphrey et al., 1996). The overlap of GLP-1(7-17), as docked to the 7TM structure and structurally aligned to the ECD, is a key feature in enabling Modeller to generate a full GLP-1R structure; these structures were used in addition to the 4L6R and 3IOL templates.

The sequences of the templates were aligned to the target sequence of GLP-1(7-36)NH_2 appended to that of GLP-1R. Six key constrains were used by Modeller in generating the model from the alignment and the template structures, namely the photoaffinity distance constraints between GLP-1R and BPA placed at 4 different peptide positions(Chen et al., 2009; Chen et al., 2010; Miller et al., 2011) and two salt-bridges between conserved residues in GLP-1R or related receptors, namely between E^9 of the peptide and TM2 and D^{15} and TM7 (Moon et al., 2015; Solano et al., 2001; Vertongen et al., 2001) (see Table S5). The effect of these 6 constraints is to supplement the quality of the model at the interface between the two templates using the best information currently available. Two thousand models were generated using Modeller, and the structure with the lowest (best) DOPE score (Shen and Sali, 2006) was selected. This model was refined in the presence of an α-helical ECL1 template derived from the CRF-R1 structure (pdb code 4K5Y (Hollenstein et al., 2013)). The α-helical portion (Q213-S219) and its orientation was determined by matching the variability (Baldwin et al., 1997; Vohra et al., 2013) of GLP-1R sequences (taken from the GPCRDB (Horn et al., 2003)) with the corresponding variability from CRF-R1 sequences; the orientation was maintained during model determination using constraints on 5 ECL1 and 5 peptide residues (Table S6). In this second phase, 5000 structures were generated using Modeller and the structure with the lowest DOPE score was selected. The structure is available from ftp://ftp.essex.ac.uk/pub/oyster/Wootten_Cell_2016/ (username: ftp, password: anonymous). The heat maps were plotted using icm (Molsoft) and these are also presented in the Supplementary file Active_movie_S1.icb that can be viewed on the free ICM browser (available from http://www.molsoft.com/icm_browser.html).

Molecular dynamics simulations. ***Surface simulation (Movie S1)***; The TM domain of GLP-1R was modeled based on the CRF1 receptor crystal structure (Hollenstein et al., 2013) using the homology modelling functionality of PLOP (Jacobson et al., 2004); the CRF1R template was chosen to give a more open structure. The N-terminus segment of GLP-1 was positioned on the surface of the extracellular loops with the help of the Maestro (Schrödinger LLC) graphical user interface. This GLP1-GLP1 complex was embedded in a lipidic POPC bilayer with explicit water and ions and simulated using ACEMD (Harvey et al., 2009), with the AMBER 14SB (Hornak et al., 2006) and lipid 14 force fields (Walker et al., 2014) for 220 ns at 300 K and 1 atmosphere pressure. The initial dimensions were 85.1 Å × 85.45 Å× 86.77 Å. ***Deep pocket simulation (Movie S2)***; The full GLP-1R in complex with GLP-1 (see modeling section) with the N-terminus of GLP-1 located deep into the TM domain, was embedded in a lipid POPC bilayer with water and ions and simulated as described for the surface simulations for 500 ns. The initial dimensions were 88.7 Å × 89.1 Å× 134.17 Å.

Data analysis. All experimental data were analysed using Prism 6 (GraphPad Software Inc., San Diego, CA, USA). For all analyses the data are unweighted and each y value (mean of replicates for each individual experiment) is considered an individual point Concentration response signalling data were analysed using a three-parameter logistic equation as previously described:

$$Y = Bottom + \frac{(Top - Bottom)}{1 + 10^{(LogEC_{50} - log[A])}} \qquad (1)$$

where *Bottom* represents the y value in the absence of ligand(s), *Top* represents the maximal stimulation in the presence of ligand(s), [A] is the molar concentration of ligand, and EC_{50} represents the molar concentration of ligand required to generate a response halfway between Top and Bottom. Similarly, this equation was used in the analysis of inhibition binding data, instead replacing EC_{50} with IC_{50}. In this case, *Bottom* defines the specific binding of the radioligand that is equivalent to non-specific ligand binding, whereas *Top* defines radioligand binding in the absence of a competing ligand, and the IC_{50} value represents the molar concentration of ligand required to generate a response halfway between Top and Bottom. IC_{50} values obtained were then corrected for radioligand occupancy as previously described using the radioligand affinity (K_i) experimentally determined for each mutant.

To quantify efficacy in the system, all data were fitted with an operational model of agonism:

$$Y = Bottom + \frac{E_m - Bottom}{1 + ((10^{logK_A}) + (10^{log[A]}))/(10^{(log\tau + log[A])})} \qquad (2)$$

where *Bottom* represents the y value in the absence of ligand(s), E_m represents the maximal stimulation of the system, K_A is the agonist-receptor dissociation constant, in molar concentration, $[A]$ is the molar concentration of ligand and τ is the operational measure of efficacy in the system, which incorporates signaling efficacy and receptor density. Constraints for this model were determined by fitting the operational model for a partial agonist to each of the peptides at the wild-type receptor, with the most efficacious peptide fitted with:

$$Y = Bottom + \frac{E_m - Bottom}{1 + 10^{(LogEC_{50} - log[A])}} \quad (3)$$

and the less efficacious peptides fitted with equation (2), in order to obtain a value for the system maximum (E_m). This value was then globally constrained in the operational model (equation (2)). All estimated τ values were then corrected to cell surface expression (τ_c) as determined by cell surface ELISA and errors propagated from both τ and cell surface expression.

Bmax from homologous competition was determined using equation 4 to radioligand binding data (^{125}I-exendin(9-39) binding by unlabelled exendin(9-39)), where Bmax is the maximum binding of ligand to receptors, [Hot] is the concentration of ^{125}I-exendin(9-39) in nM, [Cold] is the concentration of unlabelled exendin(9-39) in nM, and Kd is the equilibrium dissociation constant of the ligand in nM, with *Bottom* as defined in equation 1.

$$Y = \frac{Bmax\ x\ [Hot]}{[Hot]+[Cold]+Kd} + Bottom \quad (4)$$

Statistics. All data are represented as mean ± SEM and were compared using ANOVA followed by Dunnett's test. Repeated measures analysis of variance was used to assess the statistical significance between time courses. The null hypothesis was rejected at $P<0.05$.

Table S1. ***Related to Figure 2.*** **Effects of human GLP-1R ECL1 alanine mutants on peptide ligand binding and cell surface expression.** Binding data were analyzed using a three parameter logistic equation as defined in eq 1 in SI. pIC_{50} values represent the negative logarithm of the concentration of ligand that inhibits binding of half the total concentration of radiolabelled antagonist, ^{125}I-exendin(9-39). Data are normalized to maximum ^{125}I-exendin(9-39) binding in the absence of ligand, with non-specific binding measured in the presence of 1 μM exendin(9-39). All values are expressed as mean ± S.E.M. of three to four independent experiments, conducted in duplicate. Cell surface expression was determined through antibody detection of the N-terminal c-myc epitope label and expressed as percentage of wildtype human GLP-1R expression. All values are mean ± S.E.M. of five to six individual experiments. Bmax values were determined through homologous whole cell competition binding. Data is expressed as percentage wildtype human GLP-1R Bmax, with all values expressed as mean ± S.E.M. of three to four independent experiments performed in duplicate Data were analyzed with one-way analysis of variance and Dunnett's post test.

		Whole cell competition radioligand binding (pKi)				Cell surface expression (% wildtype)	Whole cell binding (Bmax) (% wildtype)
		GLP-1(7-36)NH_2	Oxyntomodulin	Exendin-4	Exendin(9-39)		
	Wildtype	8.76 ± 0.04	7.66 ± 0.06	9.11 ± 0.07	7.96 ± 0.04	100 ± 2	100 ± 1
**ECL1**	L201A	8.05 ± 0.13*	6.74 ± 0.14*	8.83 ± 0.13	7.66 ± 0.10	94 ± 2	94 ± 11
	K202A	8.42 ± 0.11	6.77 ± 0.07*	8.88 ± 0.11	7.59 ± 0.09	94 ± 3	110 ± 9
	W203A	8.52 ± 0.06	7.50 ± 0.05	9.00 ± 0.04	7.89 ± 0.05	94 ± 6	104 ± 3
	M204A	7.09 ± 0.18*	6.21 ± 0.24*	7.98 ± 0.07*	7.78 ± 0.84	99 ± 2	100 ± 16
	Y205A	8.25 ± 0.11	7.33 ± 0.08	8.18 ± 0.03	7.90 ± 0.09	68 ± 5*	58 ± 6*
	S206A	8.64 ± 0.10	7.76 ± 0.08	8.83 ± 0.07	8.12 ± 0.09	111 ± 6	112 ± 9
	T207A	8.67 ± 0.06	7.65 ± 0.15	8.85 ± 0.09	7.89 ± 0.10	75 ± 6*	59 ± 5*
	Q210A	8.64 ± 0.08	7.02 ± 0.12	8.81 ± 0.08	7.93 ± 0.06	88 ± 4	87 ± 11
	Q211A	8.66 ± 0.08	7.63 ± 0.10	8.73 ± 0.14	8.05 ± 0.03	112 ± 5	105 ± 6
	H212A	8.81 ± 0.07	7.84 ± 0.10	9.43 ± 0.10	7.96 ± 0.05	103 ± 3	72 ± 9
	Q213A	8.88 ± 0.10	7.74 ± 0.04	8.72 ± 0.06	7.96 ± 0.07	101 ± 2	104 ± 3
	W214A	8.20 ± 0.07*	7.44 ± 0.12	8.90 ± 0.09	7.78 ± 0.12	99 ± 5	108 ± 4
	D215A	8.34 ± 0.04	7.50 ± 0.12	*8.92* ± 0.13	7.90 ± 0.02	90 ± 2	85 ± 9
	L217A	8.72 ± 0.07	7.47 ± 0.08	8.69 ± 0.09	7.91 ± 0.06	109 ± 5	97 ± 9
	L218A	7.91 ± 0.08*	7.15 ± 0.10	8.14 ± 0.08*	7.64 ± 0.07	89 ± 6	98 ± 8
	S219A	8.82 ± 0.03	7.79 ± 0.06	9.05 ± 0.06	8.23 ± 0.09	104 ± 3	93 ± 9
	Y220A	8.49 ± 0.07	7.33 ± 0.10	9.23 ± 0.09	7.88 ± 0.07	94 ± 4	73 ± 5
	Q221A	8.53 ± 0.05	7.81 ± 0.08	8.91 ±0.05	7.69 ± 0.09	97 ± 3	87 ± 4
	D222A	8.62 ± 0.07	7.35 ± 0.04	8.95 ± 0.05	7.99 ± 0.04	102 ± 4	99 ± 17

	S223A	8.32 ± 0.07	7.32 ± 0.07	8.68 ± 0.06	8.07 ± 0.08	90 ± 5	100 ± 1
	Wildtype	8.75 ± 0.06	7.30 ± 0.07	9.22 ± 0.04	8.22 ± 0.04	100 ± 3	100 ± 1
**ECL3**	D372A	7.15 ± 0.08*	6.81 ± 0.26	8.12 ± 0.06*	7.63 ± 0.09	86 ± 4	92 ± 5
	E373A	7.08 ± 0.09*	6.43 ± 0.17*	7.95 ± 0.02*	8.15 ± 0.07	94 ± 7	99 ± 8
	H374A	8.59 ± 0.08	7.08 ± 0.16	9.10 ± 0.07	7.35 ± 0.12*	82 ± 8	89 ± 3
	R376A	8.15 ± 0.13	7.14 ± 0.13	8.90 ± 0.06	7.79 ± 0.11	92 ± 7	96 ± 6
	G377A	7.85 ± 0.06*	6.89 ± 0.10	8.79 ± 0.12	8.18 ± 0.09	96 ± 3	103 ± 4
	T378A	8.79 ± 0.09	6.67 ± 0.13	8.71 ± 0.05*	8.10 ± 0.07	173 ± 11*	153 ± 8*
	L379A	7.08 ± 0.05*	6.45 ± 0.20*	8.47 ± 0.11*	8.30 ± 0.05	90 ± 6	97 ± 3
	R380A	7.65 ± 0.13*	6.71 ± 0.29	7.75 ± 0.07*	8.03 ± 0.07	73 ± 5*	76 ± 7*
	F381A	8.42 ± 0.04	6.18 ± 0.16*	8.97 ± 0.06	6.86 ± 0.10*	92 ± 5	95 ± 1
	I382A	8.82 ± 0.10	7.37 ± 0.12	9.10 ± 0.03	8.18 ± 0.16	115 ± 6	109 ± 6
	K383A	7.76 ± 0.14*	6.40 ± 0.34*	8.17 ± 0.13*	7.29 ± 0.09*	78 ± 4*	81 ± 1*
	L384A	7.69 ± 0.12*	6.47 ± 0.30*	8.06 ± 0.08*	7.89 ± 0.15	74 ± 7*	83 ± 3
	F385A	8.97 ± 0.07	7.17 ± 0.07	9.00 ± 0.09	8.29 ± 0.09	110 ± 7	106 ± 5
	T386A	8.41 ± 0.08	7.23 ± 0.14	8.66 ± 0.05*	8.22 ± 0.07	128 ± 6	108 ± 5
	E387A	8.55 ± 0.07	6.86 ± 0.09	8.83 ± 0.08	8.03 ± 0.06	104 ± 6	101 ± 4

* Statistically significant at $p < 0.05$, one way ANOVA and Dunnett's post test in comparison to wildtype performed within the same dataset (ECL1 or ECL3).

Table S2. ***Related to Figure 4.*** **Effects of human GLP-1R ECL1 and ECL3 mutants on peptide agonist-induced cAMP accumulation.** Data were analyzed using an operational model of agonism as defined in eq 1 in SI. pEC_{50} values represent the negative logarithm of the concentration of agonist that produces half the maximal response. E_{max} E_{max} are presented as a % of the WT response. Logτ values represent the intrinsic efficacy. All Logτ values were corrected to the cell surface expression data as determined by the ELISA ($Log\tau_c$) All values are expressed as mean ± S.E.M. of four to six individual experiments, performed in duplicate. Data were analyzed with one-way analysis of variance and Dunnett's post test.

		Agonist-mediated cAMP Accumulation								
		GLP-1(7-36)NH_2			Oxyntomodulin			Exendin		
		pEC_{50}	E_{max}	$Log\tau_c$ (τ_c)	pEC_{50}	E_{max}	$Log\tau_c$ (τ_c)	pEC_{50}	E_{max}	$Log\tau_c$ (τ_c)
	Wildtype	9.66 ± 0.05	100 ± 1	1.02 ± 0.04 (10.5)	8.70 ± 0.06	100 ± 2	0.83 ± 0.05 (6.7)	10.45 ± 0.07	100 ± 2	1.03 ± 0.05 (10.7)
**ECL1**	L201A	8.47 ± 0.11*	92 ± 3	0.65 ± 0.05 (4.5)*	7.43 ± 0.15*	61 ± 4*	0.10 ± 0.19 (1.3)*	9.30 ± 0.12*	100 ± 3	0.51 ± 0.07 (3.2)*
	K202A	8.39 ± 0.08*	97 ± 2	0.37 ± 0.04 (2.4)*	8.14 ± 0.06*	98 ± 2	0.54 ± 0.10 (3.5)	9.80 ± 0.09*	104 ± 2	0.61 ± 0.05 (4.1)
	W203A	9.13 ± 0.06*	96 ± 2	0.63 ± 0.06 (4.3)	8.81 ± 0.11	90 ± 6	0.57 ± 0.05 (3.7)	10.43 ± 0.07	94 ± 2	0.88 ± 0.08 (7.6)
	M204A	7.93 ± 0.05*	105 ± 2	0.90 ± 0.07 (7.9)	7.58 ± 0.06*	100 ± 5	0.69 ± 0.17 (4.9)	9.71 ± 0.07*	107 ± 2	1.19 ± 0.07 (15.5)
	Y205A	8.90 ± 0.27*	30 ± 3*	-0.21 ± 0.09 (0.6)*	7.90 ± 0.20*	40 ± 8*	0.23 ± 0.06 (1.7)*	9.32 ± 0.25*	47 ± 8*	0.56 ± 0.08 (3.6)*
	S206A	9.78 ± 0.10	112 ± 3	1.27 ± 0.07 (18.6)	8.83 ± 0.05	109 ± 2	0.78 ± 0.05 (6.0)	10.84 ± 0.09	120 ± 3*	1.47 ± 0.08 (29.3)
	T207A	9.10 ± 0.04*	102 ± 1	0.95 ± 0.05 (8.9)	7.92 ± 0.05*	94 ± 2	0.53 ± 0.04 (3.4)	10.34 ± 0.09	102 ± 2	1.23 ± 0.07 (17.1)
	Q210A	9.63 ± 0.10	96 ± 3	0.93 ± 0.06 (8.5)	8.17 ± 0.07*	92 ± 4	0.85 ± 0.18 (7.1)	10.78 ± 0.10	99 ± 4	1.30 ± 0.11 (19.8)
	Q211A	9.14 ± 0.01*	98 ± 3	0.67 ± 0.04 (4.7)*	8.20 ± 0.08*	101 ± 4	0.45 ± 0.04 (2.8)*	10.51 ± 0.10	104 ± 4	1.13 ± 0.09 (13.5)
	H212A	9.62 ± 0.06	99 ± 3	1.10 ± 0.05 (13)	8.64 ± 0.07	100 ± 2	0.77 ± 0.04 (5.9)	10.45 ± 0.08	96 ± 2	1.06 ± 0.06 (11.6)
	Q213A	9.42 ± 0.11	100 ± 1	0.86 ± 0.07 (7.3)	8.70 ± 0.08	102 ± 3	0.48 ± 0.04 (3.1)*	10.38 ± 0.16	100 ± 5	0.96 ± 0.12 (9.0)
	W214A	8.95 ± 0.09*	97 ± 3	0.69 ± 0.04 (4.9)	8.24 ± 0.11*	92 ± 4	0.28 ± 0.04 (1.9)*	10.29 ± 0.13	104 ± 3	0.97 ± 0.10 (9.4)
	D215A	9.49 ± 0.12	105 ± 4	1.22 ± 0.38 (16.4)	8.40 ± 0.09	104 ± 3	0.42 ± 0.05 (2.6)*	10.02 ± 0.08*	107 ± 3	0.94 ± 0.06 (8.7)
	L217A	9.55 ± 0.14	97 ± 3	1.08 ± 0.08 (12.1)	8.66 ± 0.09	106 ± 4	0.66 ± 0.05 (4.6)	10.25 ± 0.05	112 ± 2	0.66 ± 0.04 (4.6)
	L218A	8.92 ± 0.07*	99 ± 2	0.87 ± 0.05 (7.4)	7.59 ± 0.10*	94 ± 4	0.41 ± 0.11 (2.6)*	9.51 ± 0.14*	100 ± 5	0.74 ± 0.09 (5.5)
	S219A	9.76 ± 0.09	101 ± 2	1.13 ± 0.06 (13.5)	8.55 ± 0.14	97 ± 7	0.57 ± 0.06 (3.7)	10.23 ± 0.09	108 ± 4	1.0 ± 0.07 (10.0)
	Y220A	9.47 ± 0.05	105 ± 2	1.09 ± 0.04 (12.4)	8.05 ± 0.10*	95 ± 7	0.50 ± 0.04 (3.1)*	10.53 ± 0.07	104 ± 3	1.33 ± 0.06 21.6)
	Q221A	9.85 ± 0.05	108 ± 1	1.38 ± 0.04 (23.9)	8.39 ± 0.10	92 ± 5	0.60 0.03 (4.0)	9.75 ± 0.08*	102 ± 2	0.75 ± 0.06 (5.6)
	D222A	9.94 ± 0.08*	99 ± 3	1.20 ± 0.06 (15.9)	8.48 ± 0.06	101 ± 2	0.57 ± 0.04 (3.7)	10.73 ± 0.08	103 ± 2	0.63 ± 0.09 (4.3)*

	S223A	*9.36 ± 0.07*	98 ± 2	0.92 ± 0.05 (8.4)	8.12 ± 0.07*	102 ± 2	0.51 ± 0.04 (3.2)	9.86 ± 0.09*	103 ± 3	0.63 ± 0.21 (4.3)*
**ECL3**	Wildtype	10.0 ± 0.09	100 ± 1	0.97 ± 0.07 (9.3)	8.43 ± 0.05	100 ± 3	0.91 ± 0.06 (8.0)	10.6 ± 0.10	100 ± 4	1.09 ± 0.11 (12.2)
	D372A	7.82 ± 0.15*	80 ± 9	0.50 ± 0.09 (3.2)*	6.63 ± 0.22*	75 ± 10	0.45 ± 0.18 (2.8)*	9.10 ± 0.11*	99 ± 5	0.99 ± 0.35 (9.8)
	E373A	6.97 ± 0.23*	64 ± 9*	0.23 ± 0.10 (1.7)*	6.92 ± 0.31*	40 ± 6*	-0.22 ± 0.11 (0.6)*	8.81 ± 0.24*	80 ± 13	0.39 ± 0.11 (2.5)*
	H374A	10.11 ± 0.08	102 ± 3	1.20 ± 0.09 (15.9)	8.49 ± 0.21	93 ± 6	0.94 ± 0.09 (8.7)	10.7 ± 0.17	101 ± 6	1.11 ± 0.13 (13.0)
	R376A	9.58 ± 0.10	101 ± 5	1.03 ± 0.16 (10.9)	8.29 ± 0.13	92 ± 4	0.72 ± 0.07 (5.2)	10.4 ± 0.14	97 ± 6	0.91 ± 0.10 (8.2)
	G377A	9.45 ± 0.11*	98 ± 4	0.83 ± 0.11 (6.7)	8.09 ± 0.16	88 ± 5	0.55 ± 0.06 (3.5)	10.5 ± 0.19	92 ± 9	0.83 ± 0.09 (6.8)
	T378A	10.6 ± 0.08*	101 ± 4	1.33 ± 0.09 (21.6)	8.44 ± 0.14	105 ± 4	0.92 ± 0.10 (8.4)	11.0 ± 0.16	108 ± 4	1.03 ± 0.11 (10.6)
	L379A	8.23 ± 0.15*	80 ± 6	0.45 ± 0.07 (2.8)*	6.95 ± 0.21*	62 ± 6*	0.13 ± 0.11 (1.4)*	10.0 ± 0.27	78 ± 13	0.45 ± 0.11 (2.8)*
	R380A	6.73 ± 0.13*	74 ± 8	0.58 ± 0.14 (3.8)*	ND*	ND*	ND*	8.33 ± 0.25*	69 ± 10*	0.36 ± 0.09 (2.3)*
	F381A	9.71 ± 0.18	74 ± 6	0.40 ± 0.05 (2.5)*	7.96 ± 0.16	80 ± 4	0.40 ± 0.10 (2.5)*	10.5 ± 0.24	88 ± 11	0.80 ± 0.10 (6.3)
	I382A	9.55 ± 0.10	96 ± 3	0.57 ± 0.05 (3.7)*	8.02 ± 0.12	86 ± 3	0.42 ± 0.06 (2.6)*	10.2 ± 0.15	86 ± 6	0.55 ± 0.07 (3.5)*
	K383A	6.89 ± 0.09*	87 ± 6	0.87 ± 0.22 (7.4)	7.22 ± 0.29*	32 ± 4*	-0.26 ± 0.12 (0.5)*	8.70 ± 0.24*	73 ± 14*	0.38 ± 0.10 (2.4)*
	L384A	8.65 ± 0.16*	91 ± 4	0.69 ± 0.08 (4.9)	7.34 ± 0.17*	92 ± 6	0.70 ± 0.12 (5.0)	10.0 ± 0.13	82 ± 2	0.50 ± 0.10 (3.1)*
	F385A	9.41 ± 0.11*	91 ± 6	0.44 ± 0.04 (2.8)*	7.84 ± 0.13	101 ± 5	0.54 ± 0.07 (3.5)*	10.3 ± 0.13	90 ± 6	0.64 ± 0.08 (4.3)*
	T386A	10.0 ± 0.13	101 ± 5	0.83 ± 0.07 (6.8)	8.56 ± 0.13	101 ± 4	0.91 ± 0.10 (8.2)	10.7 ± 0.15	98 ± 6	0.92 ± 0.11 (8.3)
	E387A	9.90 ± 0.09	108 ± 2	1.03 ± 0.08 (10.7)	7.23 ± 0.17*	61 ± 9*	0.14 ± 0.12 (1.4)*	10.3 ± 0.11	106 ± 4	0.98 ± 0.11 (9.5)

* Statistically significant at $p < 0.05$, one way ANOVA and Dunnett's post test in comparison to the wildtype response

Table S3. ***Related to Figure 5.*** **Effects of human GLP-1R ECL1 and ECL3 mutants on peptide agonist-induced intracellular calcium mobilisation.** Data were analyzed using an operational model of agonism as defined in eq 1 inSI. pEC_{50} values represent the negative logarithm of the concentration of agonist that produces half the maximal response. E_{max} are presented as a % of the WT response. Logτ values represents the intrinsic efficacy. All Logτ values were corrected to the cell surface expression data as determined by the ELISA (Logτ_c) All values are expressed as mean ± S.E.M. of four to six individual experiments, performed in duplicate. Data were analyzed with one-way analysis of variance and Dunnett's post test.

		Agonist-mediated intracellular calcium mobilisation					
		GLP-1(7-36)NH_2			Exendin		
		pEC_{50}	E_{max}	Logτ_c (τ_c)	pEC_{50}	E_{max}	Logτ_c (τ_c)
	Wildtype	7.81 ± 0.09	100 ± 4	0.23 ± 0.08 (1.68)	7.94 ± 0.09	100 ± 4	0.43 ± 0.12 (2.69)
**ECL1**	L201A	ND *	ND*	ND*	6.76 ± 0.58*	17 ± 5*	-0.94 ± 0.33 (0.11)*
	K202A	6.86 ± 0.36*	40 ± 8*	-0.85 ± 0.20 (0.14)*	6.76 ± 0.25*	39 ± 5*	-0.52 ± 0.20 (0.30)*
	W203A	7.31 ± 0.41	51 ± 9*	-0.50 ± 0.20 (0.32)*	7.89 ± 0.19	74 ± 6*	0.03 ± 0.11 (1.07)
	M204A	ND*	ND*	ND*	ND*	ND*	ND*
	Y205A	7.26 ± 0.78	14 ± 3*	-0.99 ± 0.24 (0.10)*	6.84 ± 0.78*	22 ± 7*	-0.57 ± 0.28 (0.27)*
	S206A	7.92 ± 0.14	97 ± 5	0.17 ± 0.19 (1.45)	8.25 ± 0.18	82 ± 6	0.01 ± 0.10 (1.02)
	T207A	7.81 ± 0.33	22 ± 3*	-0.76 ± 0.27 (0.18)*	7.65 ± 0.26	13 ± 1*	-0.78 ± 0.21 (0.17)*
	Q210A	7.62 ± 0.16	113 ± 7	0.34 ± 0.19 (2.18)	7.78 ± 0.26	83 ± 10	0.22 ± 0.15 (1.66)
	Q211A	7.83 ± 0.11	135 ± 7*	0.69 ± 0.24 (3.89)	8.06 ± 0.16	123 ± 8*	0.65 ± 0.17 (4.47)
	H212A	7.87 ± 0.12	109 ± 5	0.48 ± 0.20 (3.03)	8.24 ± 0.12	87 ± 4	0.35 ± 0.12 (2.24)
	Q213A	7.39 ± 0.25	74 ± 8*	-0.20 ± 0.16 (0.64)	7.83 ± 0.28	85 ± 10	0.17 ± 0.12 (1.48)
	W214A	6.80 ± 0.14*	97 ± 8	0.44 ± 0.29 (2.77)	7.68 ± 0.19	90 ± 7	0.22 ± 011 (1.60)
	D215A	7.83 ± 0.10	125 ± 5*	1.00 ± 0.32 (9.90)*	8.08 ± 0.15	117 ± 7	0.70 ± 0.16 (5.01)
	L217A	7.40 ± 0.19	84 ± 7	-0.07 0.16 (0.86)	8.13 ± 0.20	78 ± 6	0.11 ± 0.09 (1.29)
	L218A	7.03 ± 0.12*	95 ± 9	0.58 ± 0.23 (3.81)	7.64 ± 0.14	85 ± 4	0.23 ± 0.12 (1.70)
	S219A	8.14 ± 0.14	101 ± 5	0.39 ± 0.21 (2.48)	8.26 ± 0.19	90 ± 6	0.29 ± 0.13 (1.95)
	Y220A	7.73 ± 0.13	118 ± 7	0.50 ± 0.21 (3.20)	7.36 ± 0.23	90 ± 9	0.37 ± 0.17 (2.34)
	Q221A	7.46 ± 0.12	94 ± 1	0.09 ± 0.17 (1.24)	7.96 ± 0.14	93 ± 5	0.39 ± 0.13 (2.45)
	D222A	7.84 ± 0.13	97 ± 5	0.22 ± 0.18 (1.64)	7.85 ± 0.10	101 ± 5	0.44 ± 0.13 (2.75)
	S223A	7.45 ± 0.14	74 ± 5*	-0.17 ± 0.16 (0.67)	7.99 ± 0.18	71 ± 5*	-0.03 ± 0.09 (0.93)

**ECL3**	Wildtype	7.87 ± 0.12	100 ± 6	0.23 ± 0.08 (1.68)	7.99 ± 0.08	100 ± 5	0.45 ± 0.10 (2.81)
	D372A	6.83 ± 0.18*	35 ± 2*	-0.50 ± 0.09 (0.31)*	6.79 ± 0.14*	18 ± 6*	-0.83 ± 0.10 (0.15)*
	E373A	ND*	ND*	ND*	ND*	ND*	ND*
	H374A	7.69 ± 0.08	97± 8	0.20 ± 0.09 (1.59)	7.98 ± 0.09	92 ± 7	0.34 ± 0.09 (2.19)
	R376A	7.88 ± 0.19	102 ± 10	0.24 ± 0.16 (1.73)	8.01 ± 0.10	95 ± 5	0.41 ± 0.09 (2.58)
	G377A	7.47 ± 0.19	89 ± 8	-0.02 ± 0.11 (0.95)	7.94 ± 0.12	86 ± 4	0.36 ± 0.01 (2.30)
	T378A	8.42 ± 0.09	179 ± 18*	1.23 ± 0.09 (171)*	8.55 ± 0.10	157 ± 14*	1.09 ± 0.14 (12.3)*
	L379A	ND*	ND*	ND*	6.61 ± 0.17*	38 ± 10*	-1.37 ± 0.12 (0.04)*
	R380A	ND*	ND*	ND*	ND*	ND*	ND*
	F381A	7.16 ± 0.15	83 ± 11	-0.40 ± 0.05 (0.39)	7.58 ± 0.16	110 ± 9	0.28 ± 0.08 (1.92)
	I382A	7.21 ± 0.10	79 ± 7	-0.27 ± 0.05 (0.54)	7.83 ± 0.10	85 ± 10	0.18 ± 0.16 (1.53)
	K383A	ND	ND	ND*	ND	ND	ND*
	L384A	7.47 ± 0.07	82 ± 6	-0.17 ± 0.08 (0.68)	6.89 ± 0.29*	75 ± 7*	-0.89 ± 0.11 (0.13)*
	F385A	7.81 ± 0.05	85 ± 4	0.02 ± 0.04 (1.06)	8.12 ± 0.08	99 ± 6	0.46 ± 0.10 (2.88)
	T386A	7.78 ± 0.12	95 ± 9	0.03 ± 0.06 (1.07)	7.29 ± 0.12	73 ± 9	-0.20 ± 0.08 (0.63)*
	E387A	7.69 ± 0.13	98 ± 10	0.23 ± 0.08 (1.70)	8.12 ± 0.14	106 ± 8	0.49 ± 0.10 (3.09)

* Statistically significant at $p < 0.05$, one way ANOVA and Dunnett's post test in comparison to the wildtype response

Table S4. ***Related to Figure 6.*** **Effects of human GLP-1R ECL1 and ECL3 mutants on peptide agonist-induced ERK/1/2 phosphorylation.** Data were analyzed using an operational model of agonism as defined in eq 1 in SI. pEC_{50} values represent the negative logarithm of the concentration of agonist that produces half the maximal response. E_{max} are presented as a % of the WT response. Logτ values represents the intrinsic efficacy. All Logτ values were corrected to the cell surface expression data as determined by the ELISA (Logτ_c) All values are expressed as mean ± S.E.M. of four to six individual experiments, performed in duplicate. Data were analyzed with one-way analysis of variance and Dunnett's post test.

		Agonist-mediated ERK1/2 phosphorylation								
		GLP-1(7-36)NH_2			Oxyntomodulin			Exendin		
		pEC_{50}	E_{max}	Logτ_c (τ_c)	pEC_{50}	E_{max}	Logτ_c (τ_c)	pEC_{50}	E_{max}	Logτ_c (τ_c)
	Wildtype	8.33 ± 0.06	100 ± 2	0.42 ± 0.06 (2.62)	7.63 ± 0.08	100 ± 3	0.41 ± 0.05 (2.54)	8.48 ± 0.06	100 ± 2	0.44 ± 0.06 (2.74)
**ECL1**	L201A	7.37 ± 0.19*	82 ± 7	0.12 ± 0.11 (1.31)	7.93 ± 0.44	36 ± 7*	-0.44 ± 0.15 (0.36)*	7.94 ± 0.20	78 ± 6*	0.08 ± 0.05 (1.19)
	K202A	8.32 ± 0.11	111 ± 5	0.48 ± 0.18 (3.03)	7.62 ± 0.23	115 ± 11	0.59 ± 0.36 (3.90)	8.72 ± 0.17	129 ± 7*	0.89 ± 0.09 (7.72)
	W203A	7.79 ± 0.34	74 ± 10*	0.01 ± 0.06 (1.01)	6.87 ± 0.15	100 ± 9	0.08 ± 0.10 (1.20)	7.39 ± 0.40*	55 ± 11*	-0.31 ± 0.09 (0.49)*
	M204A	6.98 ± 0.12*	67 ± 5*	-0.08 ± 0.20 (0.84)*	7.35 ± 0.25	79 ± 9	0.01 ± 0.09 (1.26)	7.61 ± 0.18*	77 ± 6*	0.08 ± 0.12 (1.21)
	Y205A	7.87 ± 0.55	44 ± 9*	-0.14 ± 0.14 (0.72)*	6.15 ± 0.23*	49 ± 3*	-0.51 ± 0.22 (0.31)*	7.55 ± 0.14*	24 ± 5*	-0.68 ± 0.24 (0.22)*
	S206A	8.01 ± 0.18	83 ± 6	0.09 ± 0.07 (1.24)	7.68 ± 0.26	95 ± 11	0.32 ± 0.09 (2.10)	7.98 ± 0.18	72 ± 4*	-0.06 ± 0.07 (0.87)
	T207A	7.85 ± 0.39	58 ± 10*	0.03 ± 0.09 (1.07)	7.61 ± 0.30	53 ± 7*	0.08 ± 0.09 (1.21)	8.26 ± 0.33	36 ± 3*	-0.20 ± 0.10 (0.63)
	Q210A	8.71 ± 0.16	96 ± 5	0.54 ± 0.10 (3.43)	7.71 ± 0.13	123 ± 4	0.91 ± 0.38 (8.13)	8.22 ± 0.27	78 ± 8	0.17 ± 0.07 (1.48)
	Q211A	7.71 ± 0.23	117 ± 11	0.37 ± 0.09 (2.36)	7.89 ± 0.32	86 ± 11	0.33 ± 0.08 (2.14)	8.17 ± 0.17	95 ± 6	0.26 ± 0.07 (1.82)
	H212A	7.97 ± 0.24	109 ± 11	0.54 ± 0.07 (3.45)	7.60 ± 0.19	98 ± 8	0.53 ± 0.08 (3.40)	8.60 ± 0.17	105 ± 6	0.70 ± 0.06 (5.01)
	Q213A	7.79 ± 0.28	45 ± 3*	-0.37 ± 0.12 (0.42)*	7.25 ± 0.50	75 ± 21	0.01 ± 0.09 (1.01)	7.88 ± 0.21	80 ± 7	0.03 ± 0.08 (1.07)
	W214A	7.62 ± 0.21*	106 ± 10	0.40 ± 0.16 (2.52)	7.65 ± 0.26	85 ± 10	0.22 ± 0.07 (1.66)	8.02 ± 0.21	105 ± 9	0.32 ± 0.06 (2.07)
	D215A	7.75 ± 0.10	109 ± 5	0.55 ± 0.19 (3.52)	7.74 ± 0.25	127 ± 14	0.79 ± 0.10 (6.20)	8.65 ± 0.20	105 ± 7	0.63 ± 0.08 (4.23)
	L217A	7.37 ± 0.12*	108 ± 6	0.17 ± 0.07 (1.47)	7.43 ± 0.30	73 ± 10	0.06 ± 0.08 (1.16)	8.03 ± 0.19	64 ± 5*	-0.06 ± 0.09 (0.86)
	L218A	7.69 ± 0.20*	83 ± 7	0.12 ± 0.12 (1.31)	7.26 ± 0.22	80 ± 9	0.13 ± 0.12 (1.33)	7.85 ± 0.18	82 ± 6*	0.15 ± 0.11 (1.41)
	S219A	8.43 ± 0.13	125 ± 6	0.81 ± 0.11 (6.50)	7.86 ± 0.16	120 ± 8	0.76 ± 0.10 (5.82)	8.77 ± 0.14	91 ± 4	0.44 ± 0.07 (2.73)
	Y220A	8.13 ± 0.22	90 ± 8	0.38 ± 0.09 (2.39)	7.48 ± 0.22	77 ± 7	0.24 ± 0.09 (1.73)	8.12 ± 0.13	100 ± 5	0.45 ± 0.07 (2.82)
	Q221A	8.14 ± 0.27	86 ± 9	0.25 ± 0.08 (1.80)	7.81 ± 0.26	119 ± 8	0.77 ± 0.11 (5.90)	8.14 ± 0.23	89 ± 8	0.27 ± 0.07 (1.86)
	D222A	8.13 ± 0.25	108 ± 10	0.46 ± 0.08 (2.88)	7.39 ± 0.16	107 ± 7	0.41 ± 0.09 (2.56)	8.30 ± 0.20	104 ± 8	0.42 ± 0.06 (2.61)
	S223A	8.06 ± 0.16	141 ± 9	0.45 ± 0.11 (2.82)	7.76 ± 0.23	131 ± 12	0.84 ± 0.13 (6.91)	8.64 ± 0.25	100 ± 8	0.63 ± 0.22 (4.31)

**ECL3**	Wildtype	8.28 ± 0.11	100 ± 4	0.40 ± 0.03 (2.5)	7.57 ± 0.09	100 ± 4	0.49 ± 0.07 (3.09)	8.42 ± 0.14	100 ± 5	0.43 ± 0.19 (2.69)
	D372A	6.45 ± 0.20*	99 ± 14	-0.20 ± 0.17 (0.63)*	6.01 ± 0.08*	105 ± 21	-0.26 ± 0.11 (0.55)*	7.88 ± 0.40	60 ± 9*	-0.62 ± 0.17 (0.24)*
	E373A	6.78 ± 0.38*	59 ± 13*	-0.52 ± 0.03 (0.30)*	6.48 ± 0.25	104 ± 20	0.39 ± 0.16 (2.45)	7.27 ± 0.34	42 ± 6*	-1.05 ± 0.23 (0.09)*
	H374A	8.62 ± 0.12	88 ± 4	0.44 ± 0.09 (2.75)	7.71 ± 0.19	96 ± 8	0.30 ± 0.09 (2.0)	9.01 ± 0.18	64 ± 3*	-0.10 ± 0.22 (0.79)
	R376A	8.09 ± 0.17	77 ± 5	-0.18 ± 0.04 (0.66)*	6.99 ± 0.14	98 ± 8	0.04 ± 0.08 (1.10)	8.33 ± 0.24	79 ± 6	0.01 ± 0.16 (1.02)
	G377A	7.80 ± 0.15	87 ± 6	0.01 ± 0.02 (1.02)	7.05 ± 0.27	96 ± 13	1.53 ± 0.08 (33.9)*	8.78 ± 0.21	94 ± 6	0.58 ± 0.22 (3.80)
	T378A	8.77 ± 0.08	136 ± 4*	1.02 ± 0.24 (10.5)*	7.64 ± 0.13	192 ± 11*	-0.89 ± 0.14 (0.13)*	9.05 ± 0.15	136 ± 7*	1.33 ± 0.25 (21.4)*
	L379A	7.87 ± 0.22	52 ± 3*	-0.64 ± 0.05 (0.23)*	6.89 ± 0.23	92 ± 13	0.30 ± 0.24 (2.0)	7.70 ± 0.36	87 ± 7	0.06 ± 0.30 (1.15)
	R380A	7.15 ± 0.48*	25 ± 6*	-1.13 ± 0.04 (0.07)*	7.32 ± 0.45	30 ± 8*	1.20 ± 0.16 (15.8)*	6.94 ± 0.39*	69 ± 14	-0.34 ± 0.15 (0.46)*
	F381A	8.98 ± 0.12	115 ± 5	1.22 ± 0.44 (16.6)*	6.92 ± 0.20	105 ± 13	0.41 ± 0.12 (2.57)	8.50 ± 0.14	125 ± 5	0.92 ± 0.23 (8.32)
	I382A	8.71 ± 0.16	117 ± 6	0.98 ± 0.07 (9.55)*	7.54 ± 0.15	143 ± 9*	0.21 ± 0.12 (1.62)	8.51 ± 0.20	125 ± 8	0.92 ± 0.22 (8.32)
	K383A	6.68 ± 0.71*	38 ± 15*	-0.89 ± 0.11 (0.13)*	ND*	ND*	ND*	ND*	ND*	ND*
	L384A	7.06 ± 0.16*	99 ± 9	0.46 ± 0.13 (2.88)	6.63 ± 0.24	64 ± 10	0.03 ± 0.13 (1.07)	7.80 ± 0.11	108 ± 5	0.36 ± 0.18 (2.29)
	F385A	7.94 ± 0.17	91 ± 6	0.04 ± 0.04 (1.10)	7.24 ± 0.20	96 ± 8	-0.14 ± 0.10(0.72)*	8.47 ± 0.25	93 ± 8	0.31 ± 0.19 (2.04)
	T386A	7.76 ± 0.24	88 ± 9	-0.17 ± 0.11 (0.68)*	7.26 ± 0.11	93 ± 5	-0.21 ± 0.10 (0.62)*	8.34 ± 0.37	74 ± 9	-0.27 ± 0.15 (0.54)*
	E387A	7.79 ± 0.18	45 ± 7*	0.18 ± 0.03 (1.51)	6.96 ± 0.32	74 ± 15	-0.12 ± 0.11 (0.76)*	8.14 ± 0.21	72 ± 6	-0.31 ± 0.15 (0.49)*

* Statistically significant at $p < 0.05$, one way ANOVA and Dunnett's post test in comparison to the wildtype response

Table S5. *Related to Supplemental Experimental Precedures.* Major distance Constraints used in Modeller for the peptide docking.

Point A (GLP-1R)	GLP-1R location	Point B (GLP-1)	Evidence for constraint	constraint[c]	Reference
E125; C_{δ}	ECD	G^{35}; C_{α}	Bpa^{35} GLP-1 photoaffinity crosslink with E125.[a]	$r_{AB} \geq 20.0$ Å	Chen *et al.,* 2009
E133; C_{γ}	ECD	A^{24}; C_{β}	Bpa^{24} GLP-1 photoaffinity crosslink with E133.	$r_{AB} \leq 9.0$ Å	Chen *et al.,* 2009
L141;$C_{\delta 1}$	TM1	V^{16}; $C_{\gamma 1}$	Bpa^{16} GLP-1 photoaffinity crosslink with L141.	$r_{AB} \leq 9.0$ Å	Miller *et al.*, 2011
Y145;C_{ξ}	TM1	F^{12}; C_{γ}	Bpa^{12} GLP-1 photoaffinity crosslink with Y145.	$r_{AB} \leq 6.0$ Å	Chen *et al.*, 2010
W297;$C_{\eta 2}$	ECL2	L^{20}; C_{γ}	Bpa^{20} GLP-1 photoaffinity crosslink with W297.	$r_{AB} \leq 9.0$ Å	Miller *et al.*, 2011
K197;N_{ξ}	TM2	E^{9}; $O_{\varepsilon 2}$	Gain of function via reciprocal mutagenesis of residues between VIP and VPAC2R.[b]	$r_{AB} \leq 4.0$ Å	Solano et al., 2001
R380;$N_{\eta 2}$	ECL3	D^{15}; $O_{\varepsilon 2}$	Gain of function via reciprocal mutagenesis of residues between GLP-1 and GLP-1R.	$r_{AB} \leq 4.0$ Å	Moon *et al.*, 2015

a Constraint not used as the distance between Oε1 of E133 and Cα of G^{35} is 21.5 Å.
b The restraint gives similar results for E^{9} with K197 and/or R190; if the constraint is used with both it is relaxed to $r_{AB} \leq 6.0$ Å
c The distances used were estimated from preliminary models in which BPA was replaced by tyrosine and a 6 Å constraint was used between O_{η} of the tyrosine (topologically equivalent to the reactive carbon atom of BPA) and a suitable point on the target residue.

Table S6. *Related to Supplemntal Experimental Procedures.* ECL1 Distance Constraints and other minor constraints used in Modeller for the molecular modelling. The ECL1 constraints were used to control the orientation of ECL1 in line with the variability data and were chosen by visual analysis. Torsional constraints were also used to constrain the backbone conformation of the last four residues of the Gs C-terminal peptide.

Point A (GLP-1R)	point A Location	Point B (GLP-1R)	Point B location	constraint
M204; C_{ε}	TM2	V26; $C_{\gamma 1}$	GLP-1	$r_{AB} \leq 8.0$
W214; $C_{\xi 2}$	ECL1	V16; $C_{\gamma 1}$	GLP-1	$r_{AB} \geq 12.0$
W214; $C_{\xi 2}$	ECL1	W203; $C_{\xi 3}$	TM2	$r_{AB} \geq 5.0$
H212; $C_{\varepsilon 1}$	ECL1	F12; C_{ξ}	GLP-1	$r_{AB} \geq 10.0$
H212; $C_{\varepsilon 1}$	ECL1	V16; C_{β}	GLP-1	$r_{AB} \geq 12.0$
H212; $C_{\varepsilon 1}$	ECL1	M13; C_{γ}	GLP-1	$r_{AB} \geq 12.0$
H212; $C_{\varepsilon 1}$	ECL1	L20; C_{γ}	GLP-1	$r_{AB} \geq 12.0$
Q213; C_{δ}	ECL1	H19; C_{β}	GLP-1	$r_{AB} \geq 10.0$
D215; C_{γ}	ECL1	V16; C_{β}	GLP-1	$r_{AB} \geq 12.0$
C226; C_{γ}	TM3	C296; C_{γ}	ECL2	disulfide
D15	GLP-1	L33	GLP-1	helix
T378	Helix 8	S392	Helix8	helix
V194	TM2	A209	TM3	helix
A221	TM3	F232	TM3	helix

Supplemental References

Baldwin, J.M., Schertler, G.F., and Unger, V.M. (1997). An alpha-carbon template for the transmembrane helices in the rhodopsin family of G-protein-coupled receptors. J Mol Biol *272*, 144-164.

Chen, Q., Pinon, D.I., Miller, L.J., and Dong, M.Q. (2009). Molecular Basis of Glucagon-like Peptide 1 Docking to Its Intact Receptor Studied with Carboxyl-terminal Photolabile Probes. J Biol Chem *284*, 34135-34144.

Chen, Q.A., Pinon, D.I., Miller, L.J., and Dong, M.Q. (2010). Spatial Approximations between Residues 6 and 12 in the Amino-terminal Region of Glucagon-like Peptide 1 and Its Receptor. A region critical for biological activity. J Biol Chem *285*, 24508-24518.

Dickson, C.J., Madej, B.D., Skjevik, A.A., Betz, R.M., Teigen, K., Gould, I.R., Walker, R.C. (2014). J Chem Theory Comput *10,* 865-879.

Eswar, N., Webb, B., Marti-Renom, M.S., Madhusudham, D.E., Shen, M.Y., Pieper, U., and Sali, A. (2007). Comparative Protein Structure Modeling with MODELLER. Curr Prot in Bioinformatics, 2.9.1-2.9.31.

Friesner, R.A., Banks, J.L., Murphy, R.B., Halgren, T.A., Klicic, J.J., Mainz, D.T., Repasky, M.P., Knoll, E.H., Shelley, M., Perry, J.K.*, et al.* (2004). Glide: A new approach for rapid, accurate docking and scoring. 1. Method and assessment of docking accuracy. J Med Chem *47*, 1739-1749.

Harvey M.J, Giupponi G, and De Fabritiis G. (2009). ACEMD: Accelerating biomolecular dynamics in the microsecond time scale. J Chem Theory Comp *5,* 1632-1639.

Hoang, H.N., Song, K., Hill, T.A., Derksen, D.R., Edmonds, D.J., Kok, W.M., Limberakis, C., Liras, S., Loria, P.M., Mascitti, V.*, et al.* (2015). Short Hydrophobic Peptides with Cyclic Constraints Are Potent Glucagon-like Peptide-1 Receptor (GLP-1R) Agonists. J Med Chem *58*, 4080-4085.

Hollenstein, K., Kean, J., Bortolato, A., Cheng, R.K.Y., Dore, A.S., Jazayeri, A., Cooke, R.M., Weir, M., and Marshall, F.H. (2013). Structure of class B GPCR corticotropin-releasing factor receptor 1. Nature *499*, 438-43.

Horn, F., Bettler, E., Oliveira, L., Campagne, F., Cohen, F.E., and Vriend, G. (2003). GPCRDB information system for G protein-coupled receptors 3. Nucleic Acids Res *31*, 294-297.

Hornak V, Abel R, Okur A, Strockbine B, Roitberg A, and Simmerling C. (2006). Comparison of multiple Amber force fields and development of improved protein backbone parameters. Proteins *65*, 712-725.

Humphrey, W., Dalke, A., and Schulten, K. (1996). VMD: visual molecular dynamics. J MolGraph *14*, 33-38.

Jacobson, M.P., Pincus, D.L., Rapp, C.S., Day, T.J., Honig, B., Shaw, D.E., and Friesner, R.A. (2004). A hierarchical approach to all-atom protein loop prediction. Proteins *55*, 351-367.

Kelley, L.A., Gardner, S.P., and Sutcliffe, M.J. (1997). An automated approach for defining core atoms and domains in an ensemble of NMR-derived protein structures. Protein Engineering *10*, 737-741.

Kelley, L.A., and Sutcliffe, M.J. (1997). OLDERADO: On-line database of ensemble representatives and domains. Protein Sci *6*, 2628-2630.

Koole, C., Wootten, D., Simms, J., Miller, L.J., Christopoulos, A., and Sexton, P.M. (2012). Second extracellular loop of human glucagon-like peptide-1 receptor (GLP-1R) has a critical role in GLP-1 peptide binding and receptor activation. J Biol Chem *287*, 3642-3658.

Koole, C., Wootten, D., Simms, J., Valant, C., Sridhar, R., Woodman, O.L., Miller, L.J., Summers, R.J., Christopoulos, A., and Sexton, P.M. (2010). Allosteric ligands of the glucagon-like peptide 1 receptor (GLP-1R) differentially modulate endogenous and exogenous peptide responses in a pathway-selective manner: implications for drug screening. Mol Pharmacol *78*, 456-465.

Miller, L.J., Chen, Q., Lam, P.C.H., Pinon, D.I., Sexton, P.M., Abagyan, R., and Dong, M.Q. (2011). Refinement of Glucagon-like Peptide 1 Docking to Its Intact Receptor Using Mid-region Photolabile Probes and Molecular Modeling. J Biol Chem *286*, 15895-15907.

Moon, M.J., Lee, Y.N., Park, S., Reyes-Alcaraz, A., Hwang, J.I., Millar, R.P., Choe, H., and Seong, J.Y. (2015). Ligand Binding Pocket Formed by Evolutionarily Conserved Residues in the Glucagon-like Peptide-1 (GLP-1) Receptor Core Domain. J Biol Chem *290*, 5696-5706.

Rasmussen, S.G.F., DeVree, B.T., Zou, Y.Z., Kruse, A.C., Chung, K.Y., Kobilka, T.S., Thian, F.S., Chae, P.S., Pardon, E., Calinski, D.*, et al.* (2011). Crystal structure of the beta(2) adrenergic receptor-Gs protein complex. Nature *477*, 549-U311.

Shen, M.Y., and Sali, A. (2006). Statistical potential for assessment and prediction of protein structures. Protein Sci *15*, 2507-2524.

Siu, F.Y., He, M., de Graaf, C., Han, G.W., Yang, D., Zhang, Z., Zhou, C., Xu, Q., Wacker, D., Joseph, J.S.*, et al.* (2013). Structure of the human glucagon class B G-protein-coupled receptor. Nature *499*, 444-449.

Solano, R.M., Langer, I., Perret, J., Vertongen, P., Juarranz, M.G., Robberecht, P., and Waelbroeck, M. (2001). Two basic residues of the h-VPAC(1) receptor second transmembrane helix are essential for ligand binding and signal transduction. J Biol Chem *276*, 1084-1088.

Taddese, B., Upton, G.J., Bailey, G.R., Jordan, S.R., Abdulla, N.Y., Reeves, P.J., and Reynolds, C.A. (2014). Do plants contain G protein-coupled receptors? Plant Physiol *164*, 287-307.

Tubert-Brohman, I., Sherman, W., Repasky, M., and Beuming, T. (2013). Improved Docking of Polypeptides with Glide. J Chem Inf Model *53*, 1689-1699.

Underwood, C.R., Garibay, P., Knudsen, L.B., Hastrup, S., Peters, G.H., Rudolph, R., and Reedtz-Runge, S. (2010). Crystal Structure of Glucagon-like Peptide-1 in Complex with the Extracellular Domain of the Glucagon-like Peptide-1 Receptor. J Biol Chem *285*, 723-730.

Vertongen, P., Solano, R.M., Perret, J., Langer, I., Robberecht, P., and Waelbroeck, M. (2001). Mutational analysis of the human vasoactive intestinal peptide receptor subtype VPAC(2): role of basic residues in the second transmembrane helix. Brit J Pharmacol *133*, 1249-1254.

Vohra, S., Taddese, B., Conner, A.C., Poyner, D.R., Hay, D.L., Barwell, J., Reeves, P.J., Upton, G.J., and Reynolds, C.A. (2013). Similarity between class A and class B G-protein-coupled receptors exemplified through calcitonin gene-related peptide receptor modelling and mutagenesis studies. J RSoc Interface *10*, 20120846.
