# Supplementary figures and images for: The Extracellular Surface of the GLP-1 Receptor Is a Molecular Trigger for Biased Agonism
Source: Cell. 2016 Jun 16;165(7):1632–43. doi: 10.1016/j.cell.2016.05.023 (PMC4912689; doi:10.1016/j.cell.2016.05.023)

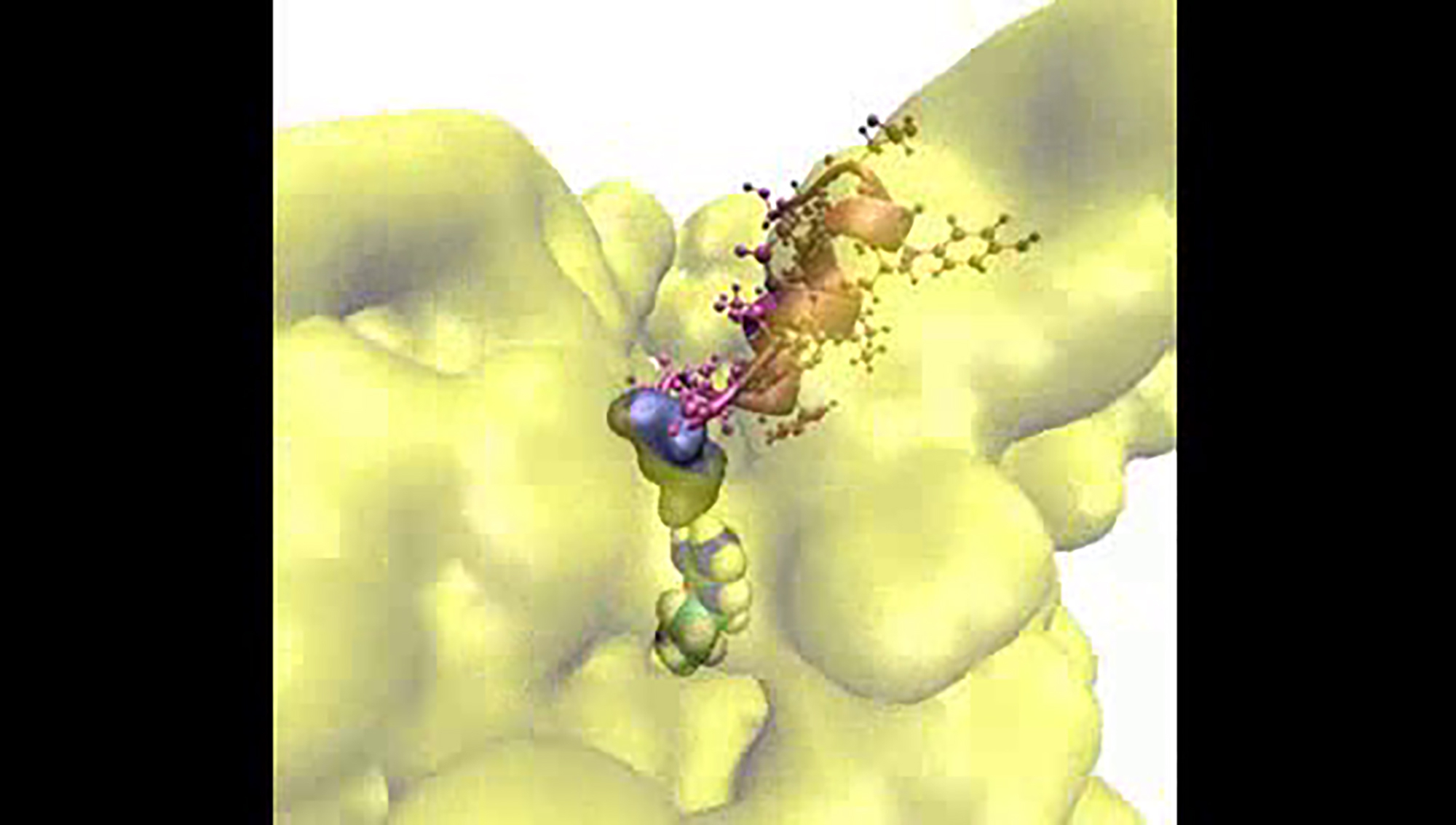

Supplement: Movie S1. Surface MD Simulation, Related to Figures 1 and 2 — The N terminus of GLP-1 initially engages superficially with the GLP-1R receptor before moving deeper into the cavity driven by the E9/R190 interaction. The receptor surface is colored yellow; GLP1 depicted in ball and stick and ribbons, both colored in mauve; E9 is depicted by its molecular surface (iced blue); and R190 is shown as CPK-colored spheres. The E9-R190 interaction holds for 220 ns. [file mmc3.jpg]

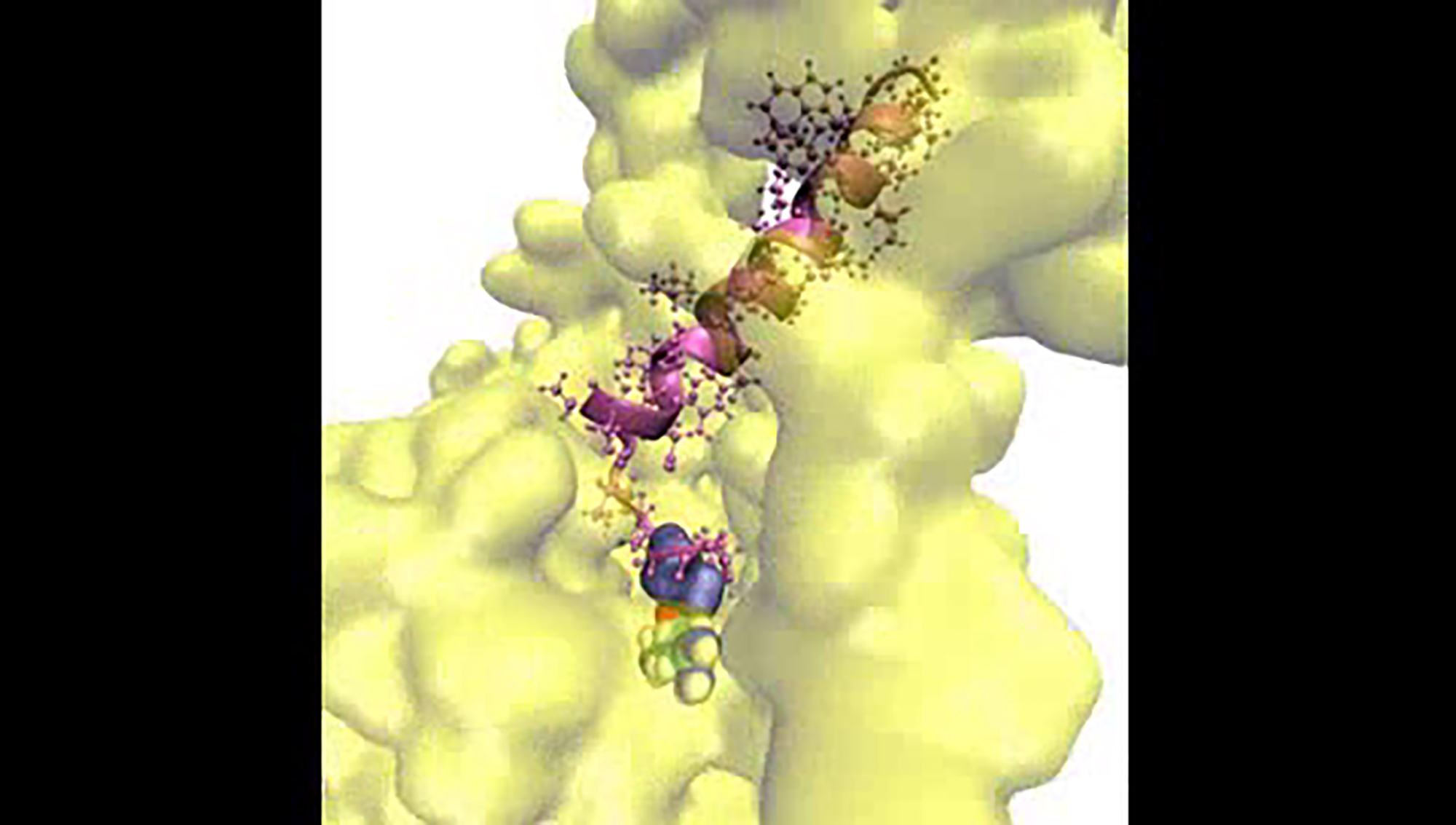

Supplement: Movie S2. Deep-Pocket MD Simulation, Related to Figures 1 and 2 — In the deep-pocket MD simulation, GLP1 and E9 start deep within the pocket and remain stable up to the full 500 ns. The color-coding is as above. [file mmc4.jpg]
