# Supplementary material for: The Extracellular Surface of the GLP-1 Receptor Is a Molecular Trigger for Biased Agonism
Source: Cell. 2016 Jun 16;165(7):1632–43. doi: 10.1016/j.cell.2016.05.023 (PMC4912689; doi:10.1016/j.cell.2016.05.023)
Supplement: Document S2. Article plus Supplemental Information [file mmc5.pdf]

Cell

Article

The Extracellular Surface of the GLP-1 Receptor Is a Molecular Trigger for Biased Agonism

Graphical Abstract

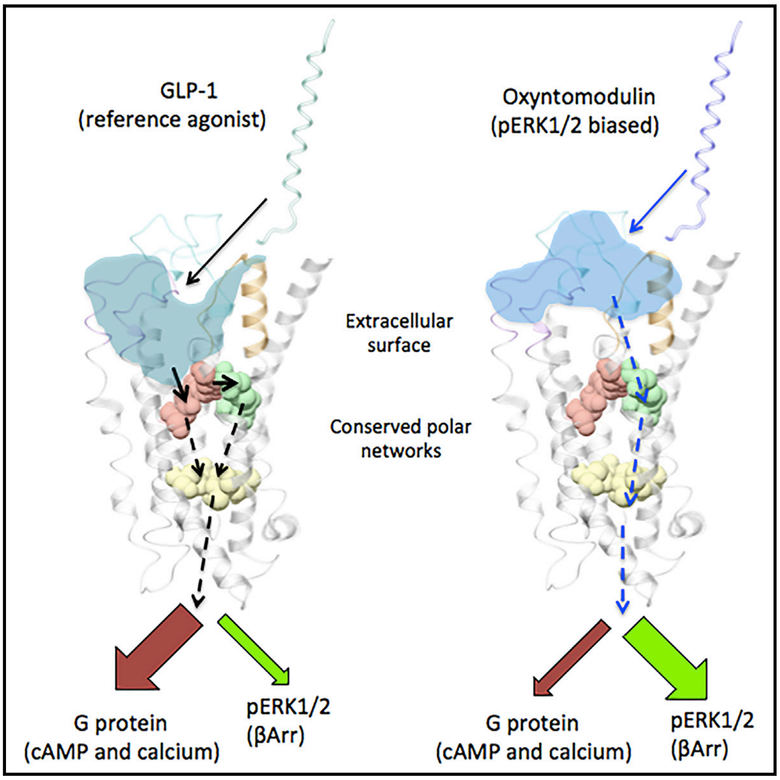

Highlights

- Effect of mutation on affinity and efficacy of biased ligands mapped onto 3D models
- Biased agonists form distinct interactions with the GLP-1R extracellular surface
- Engagement of unique elements of the extracellular surface promotes biased agonism
- Insights into class B GPCR activation/biased agonism can aid rational drug design

Authors

Denise Wootten, Christopher A. Reynolds, Kevin J. Smith, ..., Laurence J. Miller, Arthur Christopoulos, Patrick M. Sexton

Correspondence

denise.wootten@monash.edu (D.W.),
patrick.sexton@monash.edu (P.M.S.)

In Brief

Understanding how different ligands engage and activate signaling pathways downstream of the GLP-1 receptor, a key type II diabetes target, suggests strategies for shaping signaling outcomes to favor physiologically beneficial effects.

Wootten et al., 2016, Cell *165*, 1632–1643
June 16, 2016 © 2016 The Authors. Published by Elsevier Inc.
http://dx.doi.org/10.1016/j.cell.2016.05.023

Cell

Article

The Extracellular Surface of the GLP-1 Receptor Is a Molecular Trigger for Biased Agonism

Denise Wootten,[1,*] Christopher A. Reynolds,[3] Kevin J. Smith,[3] Juan C. Mobarec,[3] Cassandra Koole,[1,4] Emilia E. Savage,[1] Kavita Pabreja,[1] John Simms,[5] Rohan Sridhar,[1] Sebastian G.B. Furness,[1] Mengjie Liu,[2] Philip E. Thompson,[2] Laurence J. Miller,[6] Arthur Christopoulos,[1] and Patrick M. Sexton[1,*]

[1]Drug Discovery Biology

[2]Medicinal Chemistry

Monash Institute of Pharmaceutical Sciences, Monash University, Parkville, Victoria 3052, Australia

[3]School of Biological Sciences, University of Essex, Colchester CO4 3SQ, UK

[4]Laboratory of Chemical Biology and Signal Transduction, The Rockefeller University, 1230 York Avenue, New York, NY 10065, USA

[5]School of Life and Health Sciences, Aston University, Birmingham B4 7ET, UK

[6]Department of Molecular Pharmacology and Experimental Therapeutics, Mayo Clinic, Scottsdale, AZ 85259, USA

*Correspondence: denise.wootten@monash.edu (D.W.), patrick.sexton@monash.edu (P.M.S.)

http://dx.doi.org/10.1016/j.cell.2016.05.023

SUMMARY

Ligand-directed signal bias offers opportunities for sculpting molecular events, with the promise of better, safer therapeutics. Critical to the exploitation of signal bias is an understanding of the molecular events coupling ligand binding to intracellular signaling. Activation of class B G protein-coupled receptors is driven by interaction of the peptide N terminus with the receptor core. To understand how this drives signaling, we have used advanced analytical methods that enable separation of effects on pathway-specific signaling from those that modify agonist affinity and mapped the functional consequence of receptor modification onto three-dimensional models of a receptor-ligand complex. This yields molecular insights into the initiation of receptor activation and the mechanistic basis for biased agonism. Our data reveal that peptide agonists can engage different elements of the receptor extracellular face to achieve effector coupling and biased signaling providing a foundation for rational design of biased agonists.

INTRODUCTION

G protein-coupled receptors (GPCRs) are critical for the transmission of extracellular signals across the cell membrane to initiate intracellular responses (Fredriksson et al., 2003) and are the leading targets of currently marketed therapeutics (Overington et al., 2006). It is therefore vital to understand molecular interactions that govern ligand binding and how these interactions initiate intracellular signaling. Key advances in GPCR structural biology have greatly enhanced our knowledge of ligand interaction with GPCRs and yielded insight into receptor activation (reviewed in Katritch et al., 2013). However, to date, full-length structures have only been solved for a subset of class A GPCRs, mostly in complex with small-molecule ligands and in single inactive conformations. In contrast, there is limited information addressing the molecular details by which peptide binding at class B GPCRs couples to effector activation.

Class B peptide hormone receptors are a subfamily of GPCRs that are major targets for the treatment of chronic disease, including type 2 diabetes, obesity, and dis-regulated bone metabolism (Couvineau and Laburthe, 2012). They include receptors that bind calcitonin, calcitonin gene-related peptide, vasoactive intestinal polypeptide, pituitary adenylate cyclase-activating polypeptide, corticotropin releasing factor (CRF), gastric inhibitory polypeptide, parathyroid hormone, glucagon, and glucagon-like peptides (GLP-1 and GLP-2). Class B GPCRs share the basic seven transmembrane (TM) topology common to all GPCRs but also possess a large N terminus that forms the major binding site for selective recognition of peptide ligands (Couvineau and Laburthe, 2012). Despite sequence divergence in this region between different receptors, this extracellular domain (ECD) contains key conserved residues, including three disulphide bonds that aid in stability and confer structural similarities between receptors.

Structural data for class B receptors are limited to partial domains, including several NMR and crystal structures of peptide-bound N-terminal domains (reviewed in Pal et al., 2012) and, more recently, two inactive structures of the isolated TM core of the CRF1 receptor (CRF1R) and the glucagon receptor (GCGR) (Hollenstein et al., 2013; Siu et al., 2013). This structural data, along with structure-activity studies, support the proposed two-domain model for peptide binding to class B GPCRs, with the α-helical C terminus binding to the receptor N-terminal ECD and the peptide N terminus interacting with the extracellular face of the TM bundle (this includes the top of the TMs and the extracellular loops [ECLs]) (Pal et al., 2012). However, there is very limited information available to define these N-terminal peptide interactions with the extracellular face of the receptor core and even less to indicate how this engagement drives receptor activation. Photoaffinity and mutagenesis data highlight the significance of the core domain in both peptide binding and receptor activation, including residues within the three ECLs and their juxtamembrane regions of class B GPCRs (Barwell et al., 2011; Bergwitz et al., 1997). These studies suggest

 © 2016 The Authors. Published by Elsevier Inc.
This is an open access article under the CC BY license (http://creativecommons.org/licenses/by/4.0/).

that the extracellular face of the TM bundle forms a significant site of receptor interaction and/or plays an important role in stabilizing active receptor conformations in the presence of agonists, allowing for activation of intracellular signaling.

The GLP-1R couples to multiple effectors, and in vivo data support this as important for normal physiology in both glucose and energy homeostasis (Baggio and Drucker, 2007). The GLP-1R is an important target for treatment of type 2 diabetes mellitus, and there are multiple endogenous peptides that activate this receptor. These include four forms of GLP-1 and the related peptide oxyntomodulin (Baggio and Drucker, 2007). In addition, there are clinically approved peptides for treatment of type 2 diabetes, including exendin-4 and metabolically stabilized forms of GLP-1 (Reid, 2013). N-terminally truncated forms of these peptides are antagonists, for example exendin-4(9-39). In previous studies, we identified exendin-4 and oxyntomodulin as biased agonists relative to GLP-1 (GLP-1(7-36)NH_2) (Koole et al., 2010; Wootten et al., 2013a). The phenomenon of biased agonism describes the ability of different ligands acting at the same receptor to promote distinct cellular responses (Kenakin and Christopoulos, 2013). Intriguingly, a biased GLP-1R peptide agonist, P5, that maintains G protein signaling, while exhibiting attenuated β-arrestin recruitment, induced adiposity and was more effective at correcting hyperglycaemia in diabetic animals than exendin-4, despite having markedly lower insulinotropic properties (Zhang et al., 2015). This highlights the potential utility of biased agonists as novel GLP-1R therapeutics.

Biased agonism is currently of great interest for drug discovery, with the potential to sculpt cellular responses to favor therapeutically beneficial signaling pathways over those leading to harmful effects. However, the mechanistic basis underlying biased signaling needs to be understood if this is to be exploited for rational drug design. Pleiotropic coupling of the GLP-1R leads to cAMP production, Ca^{2+} mobilization, and phosphorylation of ERK1/2 (pERK1/2) (Koole et al., 2010), each of which are physiologically important (Baggio and Drucker, 2007). The contribution of these signaling pathways and the extent to which one is activated relative to another is therefore important for optimal development of therapeutics. Existing data demonstrate that biased signaling does indeed occur at the GLP-1R; however, the mechanistic basis for this is unknown (Koole et al., 2010; Wootten et al., 2013a).

Using a combination of alanine-scanning mutagenesis, followed by pharmacological quantification of the effects of mutation on peptide agonist affinity and three distinct signaling pathways, we have identified critical regions within the extracellular face of the receptor core both for peptide agonist affinity and for driving receptor coupling to distinct signaling pathways, extending our initial work on ECL2 (Koole et al., 2012). We used a GLP-1R model in conjunction with experimental data to generate comparative heatmaps of the contribution of the extracellular surface to agonist affinity and signaling efficacy. These revealed distinct elements of the extracellular face of the GLP-1R that are engaged to activate individual signaling pathways in a ligand-dependent manner. Collectively, the work allows us to yield novel molecular insights into the initiation of receptor activation and the mechanistic basis for biased agonism at this important class B GPCR. This provides a framework to enable future design of agonists with tailored signal bias for this receptor.

RESULTS

To understand the functional interface at the GLP-1R extracellular surface, we completed alanine-scanning mutagenesis of the ECLs and adjacent TM residues, coupled with analysis of ligand affinity and signaling for three key pathways that are involved in GLP-1R function and rely on different effector engagement (Figure S1). We assessed three peptides (GLP-1, oxyntomodulin, and exendin-4) with highly conserved N-terminal sequences that display biased agonism (Figure S2). This biased agonism can be observed in both the recombinant cells used in this mutagenesis study and natively expressing insulinoma cells that display key features of β islets, where both exendin-4 and oxyntomodulin were biased away from GLP-1 in promotion of cellular proliferation and reducing apoptosis, compared to cAMP signaling (Figure S2). In addition to measurements of agonist binding affinity, the effects on signaling efficacy for each of the pathways were quantified using an operational model of agonism (Black and Leff, 1983). This enables comparison of effects of mutations across the different signaling pathways and reveals how individual peptide ligands interact with the receptor surface to elicit signaling. To understand the importance of residues in ligand binding and function, we developed a full-length, GLP-1-bound, GLP-1R model (Active model S1) (Wootten et al., 2016). Residues located within the ECL/TM boundaries of the N-terminal ECD are numbered based on their location in the protein sequence. Residues that are located within the TM bundle also contain, in superscript, the class B numbering described in Wootten et al. (2013b).

The predicted ECL1 and adjacent TM boundary comprises 23 residues from L201 to S223, ECL2, 23 residues from G285 to L307 and ECL3, 16 residues from D372 to E387. The results for the pharmacology of ECL2 mutants have been published previously (Koole et al., 2012) and are discussed here, along with novel data on ECL1 and ECL3, in context of the 3D surface map developed for the receptor.

All mutant receptors, with the exception of W306A, were expressed at the cell surface, and most were expressed at levels equivalent to wild-type (Table S1) (Koole et al., 2012). Of the ECL1 and ECL3 mutants, only three exhibited a change in antagonist binding: H374A, an effect specific to exendin-4 (9–39); K383A, which had global effects on peptide binding; and F381A, which had selective effects dependent on the peptide (Table S1). Given that only limited mutations grossly altered cell-surface expression and antagonist affinity, altered effects on receptor function (affinity and efficacy) for most engineered mutations are likely a result of loss of direct interactions with ligands (affinity) or of altering (either directly or indirectly) interactions between receptor side chains and disruptions to hydrogen bonding networks that are crucial for the receptor to explore its conformational landscape, thereby indicating residues that are important in the mechanism of signal propagation.

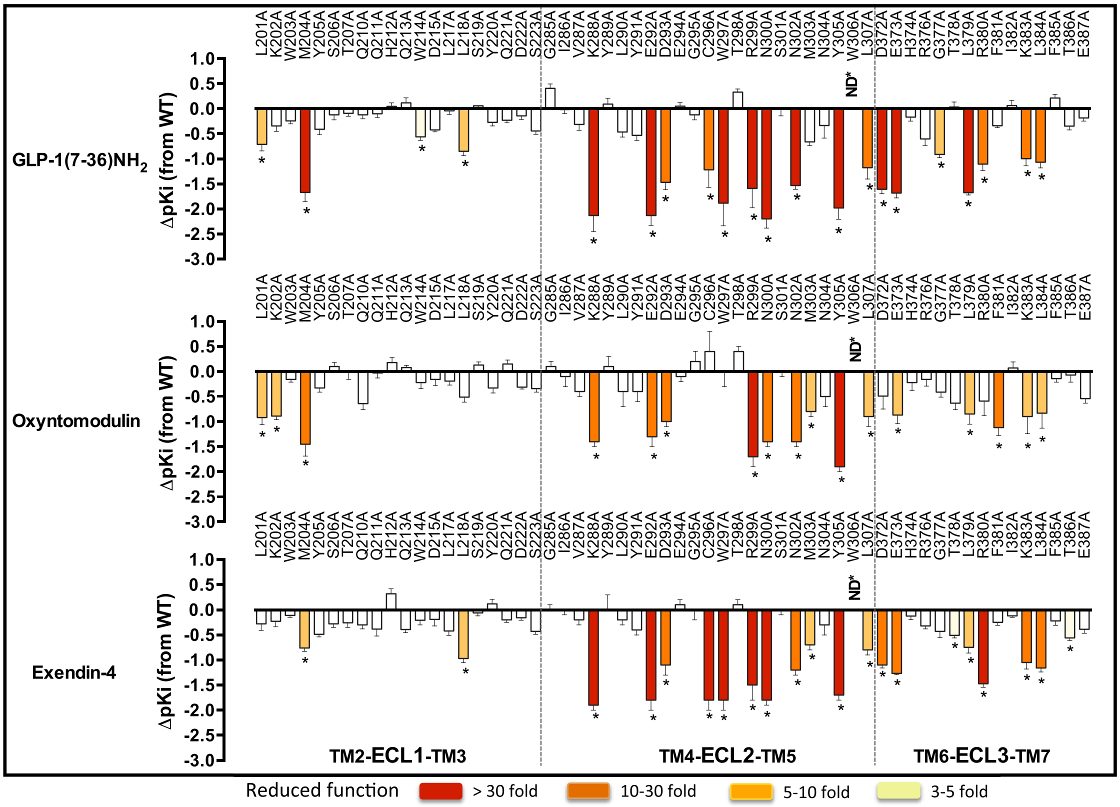

Figure 1. Agonist Affinity Profiles of GLP-1R ECL Alanine Mutants Reveal the Importance of Individual Residues for Peptide Affinity

pKi values for each peptide were derived from radioligand inhibition-binding experiments. Bars represent differences in calculated affinity (pKi) values for each mutant relative to the wild-type receptor for GLP-1 (top), oxyntomodulin (middle), and exendin-4 (bottom). Statistical significance of changes in affinity in comparison with wild-type was determined by one-way analysis of variance and Dunnett's post-test, and values are indicated with an asterisk (*p < 0.05). Data that are statistically significant are colored based on the extent of effect. All values are ± SEM of four to six independent experiments, conducted in duplicate.

Involvement of the Extracellular Surface in Agonist Peptide-Binding Affinity

Effects of mutations on agonist affinity were established by heterologous competition with the antagonist radioligand (^{125}I-exendin-4 (9–39)) (Table S1) (Koole et al., 2012). The affinity measures for each mutant were compared to the wild-type to determine the relative importance of each individual residue in peptide agonist affinity (Figure 1). These were mapped onto the 3D model to provide a comparative heatmap of the contribution of the extracellular surface to agonist affinity (Figure 2, Active model S1).

Overall, there was a high degree of overlap in the impact of alanine mutation on binding of GLP-1, exendin-4, and oxyntomodulin. In 3D space, there is a continuum of residues from K288, E292, D293, R299, N300 within the proximal part of ECL2 that link to TM6/ECL3 membrane-proximal residues D372, E373 and L379, K383, L384 in ECL3 that are globally important for binding affinity, along with most residues in the distal segment of ECL2 (Table S1, Figures 1 and 2, Active model S1).

In addition, M204 at the TM2/ECL1 boundary lines the peptide-binding groove in our model and is important for the affinity of all peptides. There is an additional network of residues deeper in the protein (C296, W297, R380) that are important for GLP-1 and exendin-4 affinity but have little role in oxyntomodulin binding (Table S1, Figures 1 and 2, Active model S1). L218 within ECL1 is also important for the affinity of GLP-1 and exendin-4 but not oxyntomodulin. Additionally, L201 lies deeper in the peptide groove of the protein and is important for GLP-1 and oxyntomodulin affinity but not exendin-4 (Table S1, Figures 1 and 2, Active model S1). Only a limited number of residues were selectively important for affinity of individual peptides: W214 (ECL1) and G377 (ECL3) for GLP-1; K202 (ECL1) and F381 (ECL3) for oxyntomodulin; and T378 and T386 (both in ECL3) for exendin-4.

Of all the residues important for peptide affinity, most are likely to have indirect effects on peptide binding. Alterations to agonist affinity can be achieved by the mutation either altering the conformation of residues that directly interact with the peptide within the binding pocket or altering the shape of the binding pocket such that the peptide cannot bind in the same manner. The only ECL side chains that our modeling predicted to interact directly with GLP-1 are L201, W297, R299, N300, and R380 (Figure S3). This includes three residues that, when mutated, have differential effects on peptide affinity. Although all are important for GLP-1 affinity, alanine mutation of L201 had little effect on exendin-4 affinity, and mutation of W297 and R380 had no effect on

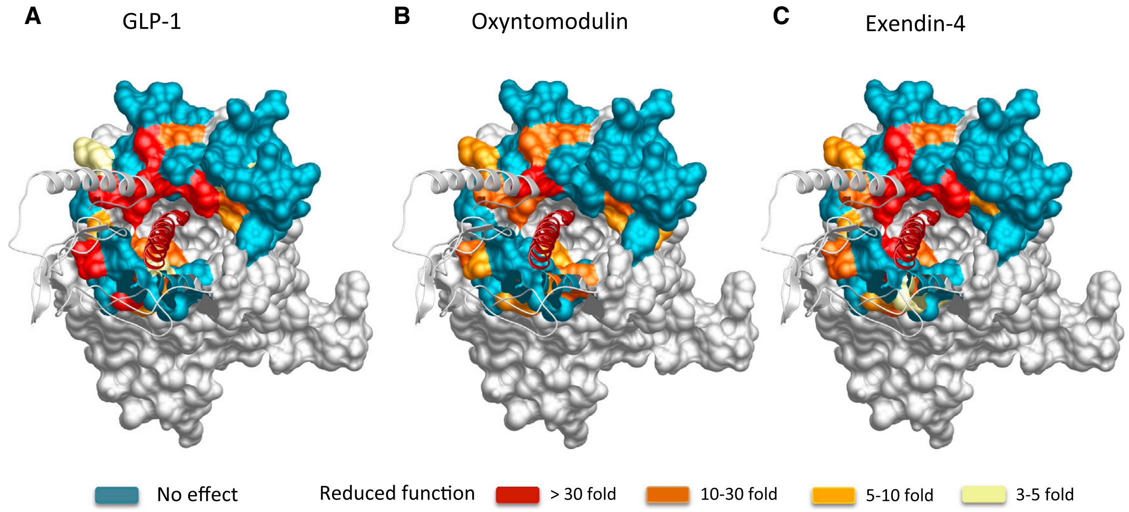

Figure 2. Heatmap 3D Representation of the GLP-1R Extracellular Face Based on Affinity-Binding Data

Molecular model of the GLP-1R-GLP-1 complex showing the extracellular surface of the TM bundle. Residues that altered affinity of GLP-1 (A), oxyntomodulin (B), and exendin-4 (C) when mutated are highlighted. Teal indicates residues that were assessed and did not alter affinity; yellow (3- to 5-fold), pale orange (5- to 10-fold), orange (10- to 30-fold), and red (>30-fold) are residues that statistically altered affinity.

oxyntomodulin affinity (Figures 1 and 2, Table S1). This is particularly interesting, as the residues in the GLP-1 peptide that are predicted to interact with these side chains are absolutely conserved in the N terminus of the three peptide ligands (Figure S3). This implies that differential interactions of the C terminus of the peptides with the N-terminal ECD may differentially orient the N terminus of these peptides in the binding groove such that they form distinct interactions with the bundle.

Involvement of the Extracellular Surface in Ligand Efficacy

Agonist potency is a composite of efficacy and affinity and cannot be used to distinguish pathway-specific effects of mutations. In contrast, in the operational model, the efficacy term "τ" relates receptor occupancy to magnitude of response for an individual pathway and is independent of ligand affinity, although not receptor expression levels. However, τ values can be normalized to experimentally determined levels of cell-surface expression to provide a measure of pathway activation (τ_c) that is independent of both affinity and cell-surface expression levels (Kenakin and Christopoulos, 2013). Concentration response curves for each of the peptides for cAMP formation, pERK1/2, and Ca^{2+} mobilization were established for wild-type and each receptor mutation to determine EC_{50} and E_{max} and τ_c values for each pathway for all mutants (Tables S2, S3, and S4). As with affinity, τ_c estimates for each mutant receptor were compared to the wild-type to determine the relative importance of each residue for efficacy in each pathway (Figures 3, 4, and 5). These were mapped onto the 3D model to provide a comparative heatmap of the contribution of the extracellular surface to efficacy for individual pathways (Figure 6, Active model S1). Overall, there was a significant correlation between residues identified as important for peptide affinity, cAMP formation, and Ca^{2+} mobilization. Generally, there was less correlation between agonist affinity and pERK1/2 efficacy with a distinct pattern of residues in 3D space being important for transmitting efficacy down this pathway (Figures 6 and 7).

Involvement of the Extracellular Surface in Peptide-Mediated cAMP Formation

Consistent with binding studies, there was a high degree of overlap in the impact of alanine mutation on cAMP-mediated signaling by GLP-1, exendin-4, and oxyntomodulin (Figures 3 and 6; Tables S1 and S2). These residues were concentrated mainly within ECL2 and the more buried, membrane-proximal regions of ECL1 and ECL3 that included residues deep within the binding groove important for affinity of these peptides (Figures 3 and 6A–6C, Active model S1). However, when mutated, these residues had a smaller effect on cAMP efficacy than they did on affinity (Figures 1, 2, 3, and 6A–6C). An additional residue, Y205, proximal to the binding groove at the TM2/ECL1 boundary was important for cAMP efficacy by all three peptides. Furthermore, two residues within the peptide binding groove (W297 and R380) that were important for GLP-1 and exendin-4, but not oxyntomodulin, affinity were required for all three peptides to activate this signaling pathway. Here the effect of mutation was larger for oxyntomodulin (no appreciable cAMP response) (Figures 2, 3, and 6; Table S2) (Koole et al., 2012). GLP-1 and exendin-4 also engage a large proportion of ECL2 and ECL3 with only minor contributions from ECL1 for transmission of efficacy to the cAMP pathway (Figures 3, 6A, and 6C). In contrast, a large proportion of all three loops contributed to cAMP signaling via the peptide oxyntomodulin, with more involvement of ECL1 but less involvement of ECL2 compared with GLP-1 and exendin-4 (Figures 3 and 6B). The heatmaps indicate that the lower-affinity ligand oxyntomodulin engages regions (albeit not necessarily the same residues) in the extracellular surface similar to those of GLP-1 and exendin-4 upon binding to the receptor

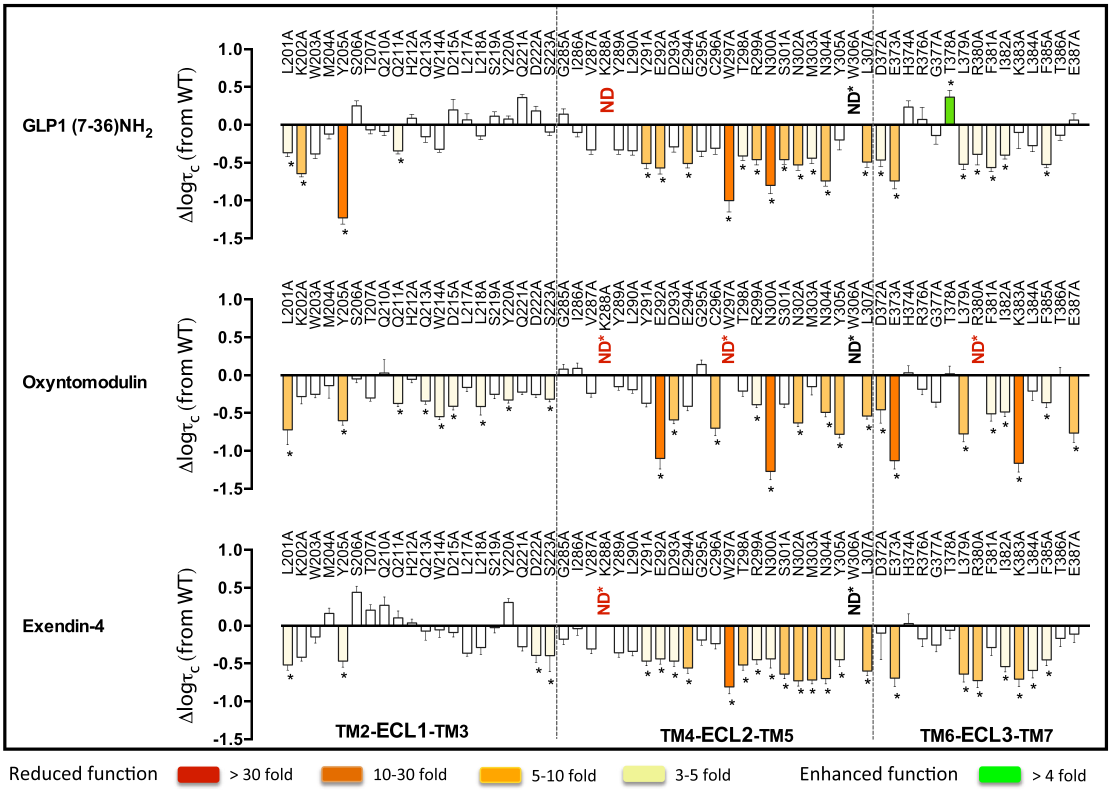

Figure 3. Peptide-Dependent Effects of ECL Mutations on cAMP Efficacy

Differences in the coupling efficiency ($\log\tau_c$) for cAMP formation of ECL mutations, compared to the wild-type receptor, by GLP-1 (top), oxyntomodulin (middle), and exendin-4 (bottom). Statistical significance of changes in coupling efficacy was determined by one-way analysis of variance and Dunnett's post-test, and values are indicated with an asterisk (*$p < 0.05$ compared with wild-type). Data that are statistically significant are colored based on the direction and extent of effect. All values are $\log\tau_c$ ± SEM of four to six independent experiments, conducted in duplicate.

but requires distinct regions of this surface to promote conformational transitions leading to formation of cAMP (Figures 2 and 6, Active model S1).

Despite the critical importance of ECL2 and the membrane-proximal region of ECL3 for all three peptides to couple to cAMP, the relative contribution of each individual residue within this region varied considerably between oxyntomodulin and the other two peptides (Figure 7). This included a number of residues within ECL2 (Y291, E294, T298, S301, and M303) that were required for GLP-1- and exendin-4-mediated cAMP accumulation but not for oxyntomodulin and two residues, C296 in ECL2 and E387 in ECL3, that had the reverse profile. In addition, there were a number of residues that had global effects across all three peptides but with different magnitudes in the extent of effect (Figures 3, 6, and 7; Table S2). Only a very limited number of residues were selectively important for cAMP efficacy between GLP-1 and exendin-4. K202 (ECL1) was selective for GLP-1 only, and Q211 (ECL1), D372, and I382 (ECL3) affected both GLP-1 and oxyntomodulin but not exendin-4. D222 (ECL1) and L384 (ECL3) were selective for exendin-4 only, whereas mutation of S223 (ECL1), D293, Y305 (ECL2), and K383 (ECL3) altered cAMP signaling by both exendin-4 and oxyntomodulin but not GLP-1 (Figures 3, 6, and 7; Table S2).

Involvement of the Extracellular Surface in Peptide-Mediated Intracellular Calcium Mobilization

Due to the low efficacy of oxyntomodulin for promoting Ca^{2+} mobilization, this pathway was only assessed for GLP-1 and exendin-4 (Figures 4, 6D, and 6E, Active model S1). Consistent with binding and cAMP data, a continuum of residues in 3D space within ECL2 (K288, E292, D293, C296, W297, R299, N300) linking the membrane-proximal residues in ECL3 (D372, E373, L379, R380, K383), along with most residues in the distal segment of ECL2, were required for both ligands to promote Ca^{2+} mobilization. However, mutation of these residues had a larger impact on exendin-4 than on GLP-1, with more mutant receptors unable to produce a detectable exendin-4-mediated Ca^{2+} response (Figures 4, 6D, and 6E). In addition, residues within the TM2/ECL1 membrane-proximal region (L201, K202, M204, Y205, T207) were globally important for both peptides. There were also additional residues within the TM4/ECL2 (I286, V287, Y289, L290, Y291) and the ECL3/TM7 (L384, T386) membrane-proximal portions of ECL2 and ECL3, respectively, that were important for exendin-4-mediated signaling to this pathway, but with little role for GLP-1 coupling (Figures 4, 6D, and 6E). These residues extend within 3D space from the continuum of residues that are globally important.

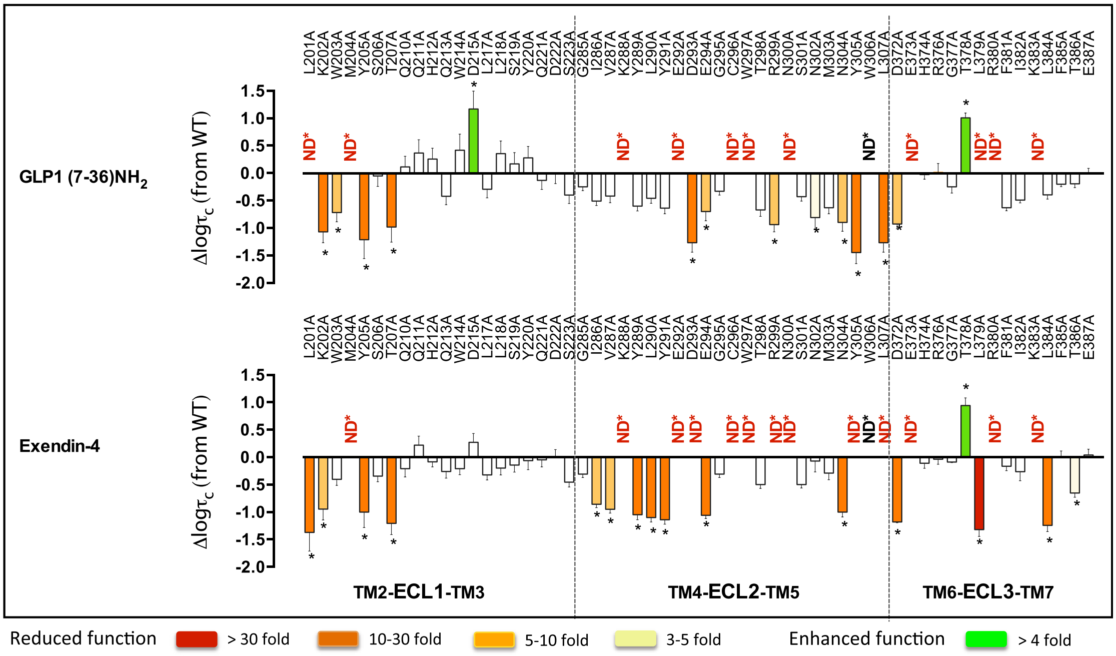

Figure 4. Peptide-Dependent Effects of ECL Mutations on Efficacy for Ca^{2+} Mobilization

Differences in the coupling efficiency ($\log\tau_c$) for Ca^{2+} mobilization of ECL mutations, compared to the wild-type receptor, by GLP-1 (top) and exendin-4 (bottom). Statistical significance of changes in coupling efficacy was determined by one-way analysis of variance and Dunnett's post-test, and values are indicated with an asterisk (*$p < 0.05$ compared with wild-type). Data that are statistically significant are colored based on the extent of effect. All values are $\log\tau_c$ ± SEM of four to six independent experiments, conducted in duplicate.

Interestingly, mutation of two residues (D215 in ECL1 and T378 in ECL3) lying outside of the predicted peptide-binding groove enhances the ability of GLP-1 to promote Ca^{2+} mobilization, and one of these residues (T378) also had the same effect for exendin-4 (Figures 4 and 6).

Involvement of the Extracellular Surface in Peptide-Mediated pERK1/2

Mapping mutational effects for coupling to pERK1/2 onto the 3D model revealed a strikingly distinct pattern in regions of the GLP-1R extracellular face that were involved in coupling to this pathway, in comparison to those important for affinity, cAMP, and Ca^{2+} mobilization. Whereas these latter aspects of receptor function required a large area of the protein's extracellular surface for transmission of signal, residues important for pERK1/2 were localized mainly to membrane-proximal residues of ECL3 (Figures 5 and 6F–6H, Active model S1) with very little involvement (at least for GLP-1 and exendin-4) of ECL2 (the most critical domain for all other assessed aspects of receptor function). Only one residue throughout the entire extracellular surface, Y205 in ECL1, was globally important for coupling all three peptides to cAMP, iCa^{2+}, and pERK1/2 (Figures 3, 4, 5, 6, and 7).

Despite all three peptides utilizing ECL3 for coupling receptor activation to pERK1/2, the importance of individual residues within this loop varied between the ligands. D372, T378 and R380, and T386 were globally important for signaling by all three peptides, but for T378 and R380, the effect of mutation varied. Interestingly, T378A increased the efficacy for GLP-1 and exendin-4 but had the opposite effect on oxyntomodulin, reducing its efficacy. The reverse effect was observed for R380A, where oxyntomodulin efficacy was increased and GLP-1 and exendin-4 efficacies were both impaired (Figures 5, 6F–6H, and 7). This implies that these residues may be important for conformational switching of the receptor, altering the ensemble of conformations that allow for coupling to this pathway. In addition, within this loop, E373 was required for both GLP-1 and exendin-4 but played little role in the ability of oxyntomodulin to activate this pathway. Furthermore, mutation of R376, L379, F381, and I382 significantly altered signaling by GLP-1, with a similar trend displayed by exendin-4 but little role in oxyntomodulin-mediated pERK1/2 (Figure 5).

Although, compared to GLP-1, exendin-4 utilized a larger proportion of the extracellular surface for coupling the GLP-1R to iCa^{2+} mobilization, mutational effects on pERK1/2 were even more confined to ECL3 than those for GLP-1. Intriguingly, despite requiring a very large portion of ECL2 for intracellular Ca^{2+} mobilization, this domain played no role in coupling exendin-4 binding to pERK1/2. ECL2 played a limited role for GLP-1 coupling to this pathway with E292 and N300 being important. These residues were also important for oxyntomodulin coupling to pERK1/2 along with D293, N302, and Y305 (Figures 5, 6F, and 6G).

Additional residues selectively important for pERK1/2 by individual peptides included M204 and Q213 (ECL1) for GLP-1; L201 (ECL1) and F385 (ECL3) for oxyntomodulin; and W203 (ECL1) for exendin-4.

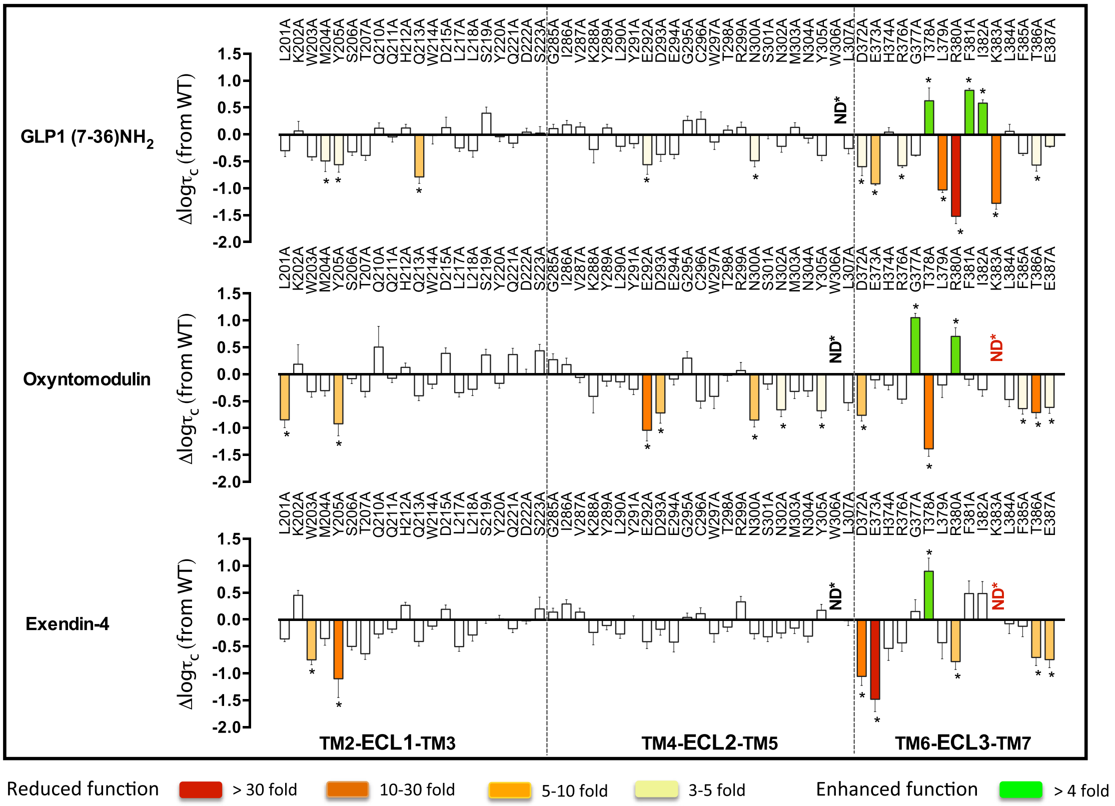

Figure 5. Peptide-Dependent Effects of ECL Mutations on pERK1/2 Efficacy

Differences in the coupling efficiency ($\log\tau_c$) to pERK1/2 of ECL mutations, compared to the wild-type receptor, by GLP-1 (top), oxyntomodulin (middle), and exendin-4 (bottom). Statistical significance of changes in coupling efficacy was determined by one-way analysis of variance and Dunnett's post-test, and values are indicated with an asterisk (*$p < 0.05$ compared with wild-type). Data that are statistically significant are colored based on the direction and extent of effect. All values are $\log\tau_c \pm$ SEM of four to six independent experiments, conducted in duplicate.

DISCUSSION

Ligands binding to GPCRs modify the conformational landscape and thus stabilize a subset of conformational ensembles, providing the basis for both differential efficacy and biased agonism. There is limited information linking the dynamic events of receptor activation to engagement of specific effector proteins, and this is particularly true for class B GPCRs. The current study explores the molecular determinants for ligand affinity and engagement of signaling. Specifically, we highlight crucial surface residues within a class B GPCR that link initial peptide agonist interactions to distinct intracellular signaling pathways and biased agonism.

Peptide interactions with the extracellular surface and TM domains of class B GPCRs promote conformational transitions required to allow the binding of signaling effectors at the intracellular surface of these receptors. The ability of receptor mutants to affect signaling at only a single pathway highlights that different elements of the extracellular face are required for coupling to different effectors. In addition, differential effects on signaling by the three peptide agonists following mutation of individual residues supports the notion that the extracellular face of the receptor is important for initiating a switch in the conformational landscape that the receptor explores, with different ligands capable of promoting/stabilizing alternative subsets of ensembles that lead to biased agonism.

Importance of the ECL Regions of the GLP-1R for Peptide Binding

Large portions of ECL2, ECL3 and the juxtamembrane positions of TM2/ECL1, ECL2/TM5 and TM6/ECL3 were important for molecular recognition of all peptide agonists but not for binding of the N-terminally truncated antagonist exendin-4(9–39). Despite the separation in sequence of these residues, they are all located together in 3D space. In addition, there is a network of residues provided by all three loops lining the cavity entrance in the TM bundle, and these residues are important for GLP-1 affinity, extending the peptide-binding groove from the N-terminal ECD into the TM domain cavity. These residues include L201, M204 (TM2/ECL1), E294, W297, T298, R299, N300, Y305 (ECL2), R380, L384 (ECL3) (Figure S4). Interestingly, most of these residues appear to have indirect effects on agonist affinity as only five of these residues interact directly with GLP-1 in our model (L201, W297, R299, N300 and R380) (Figure S3).

The peptide-binding groove in the molecular model extends from the ECLs down into the TM bundle, forming a deep cavity

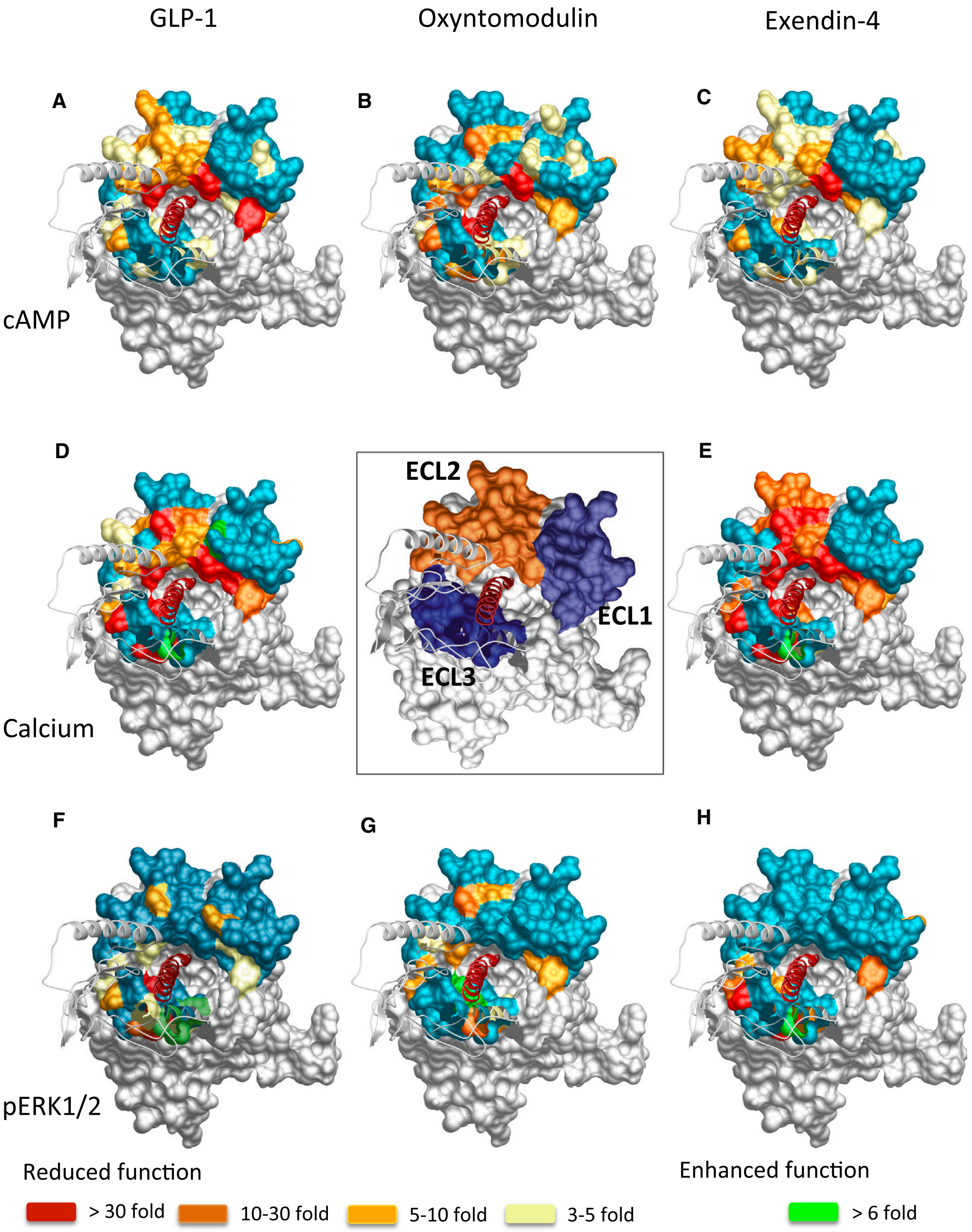

Figure 6. Heatmap 3D Representation of the GLP-1R Extracellular Face Based on Efficacy Data from Three Different Signaling Assays

Molecular model of the GLP-1R-GLP-1 complex showing the extracellular surface of the TM bundle. All residues assessed in this study are shown in the center box; the locations of residues in ECL1, ECL2, and ECL3 are highlighted in purple, orange, and blue, respectively. Residues that when mutated altered efficacy are highlighted in (A)–(H). (A–C) cAMP efficacy of GLP-1 (A), oxyntomodulin (B), and exendin-4 (C); (D and E) Ca^{2+} efficacy of GLP-1 (D) and exendin-4 (E); (F–H) pERK1/2 efficacy of GLP-1 (F), oxyntomodulin (G), and pERK1/2 (H). Teal indicates residues that were assessed and did not alter efficacy; yellow (3- to 5-fold), pale orange (5- to 10-fold), orange (10- to 30-fold), and red (>30-fold) are residues that statistically altered efficacy. The 3D heatmaps can be found in Active model S1 (Data S1).

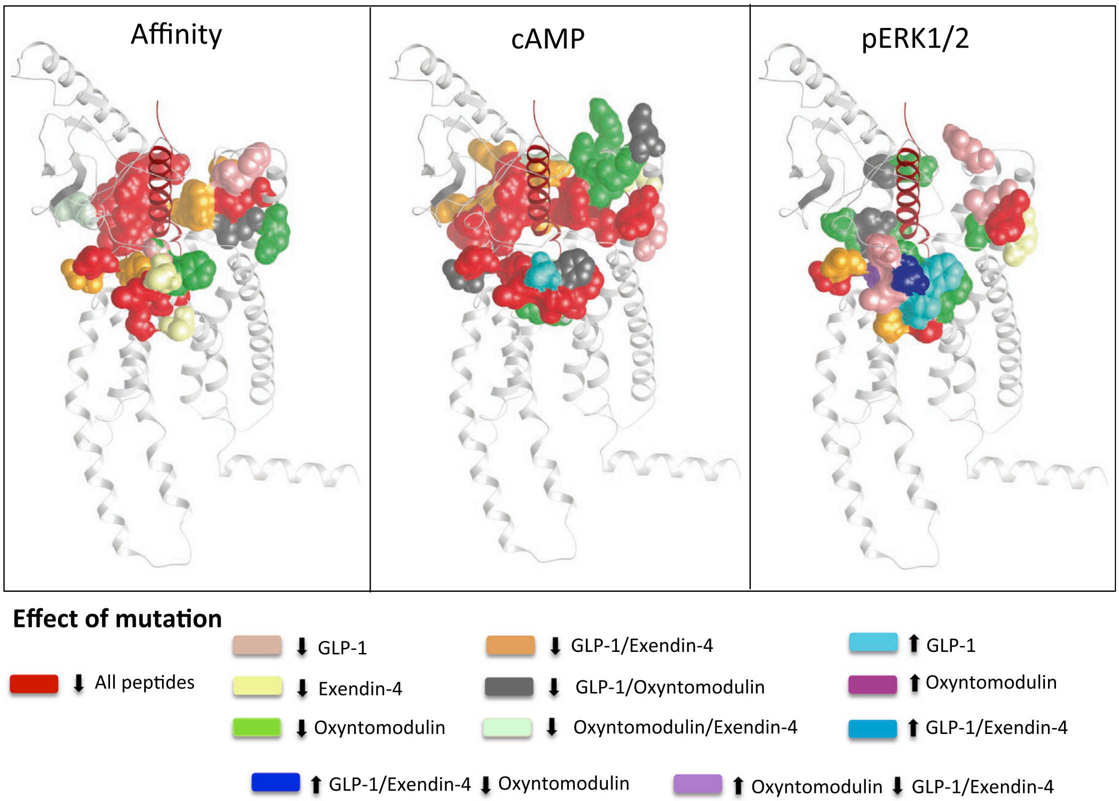

Figure 7. 3D Model Illustrating GLP-1R ECL Loop Residues that Are Globally or Selectively Important for GLP-1, Oxyntomodulin, and Exendin-4

Based on statistical significance ($p < 0.05$) of effect when mutated to alanine, experimentally observed effects on peptide affinity and efficacy can be mapped onto the molecular model to clearly highlight similarities and differences between the three peptide agonists. Residues highlighted in red reduce function (affinity [A] or efficacy [B and C]) of all three peptides, those in pink selectively reduce GLP-1 only, those in yellow selectively reduce exendin-4 only, and those in green selectively reduce oxyntomodulin only. A large number of residues are important for both GLP-1 and exendin but not oxyntomodulin, and these are highlighted in orange. Other colors represent either enhanced function (GLP-1 only/oxyntomoduin only or GLP-1 and exendin) or existence of opposite effects when mutated on oxyntomodulin compared to GLP-1 and exendin-4.

lined by residues in all TMs except TM4 (Figure S4). In addition to the identified residues within the ECLs, published information on the requirement of other residues within this proposed cavity for GLP-1 affinity support our molecular model wherein GLP-1 enters into this cavity upon binding, with its N terminus residing deep within the helical bundle in the final ligand-docked model (Figures S3, S4, S5, and S6, Active model S1). Four of the residues that reside at the bottom of this pocket (R190$^{2.60}$, N240$^{3.43}$, E364$^{6.53}$, and Q394$^{7.49}$) form part of a hydrogen bond network in the inactive, unliganded receptor and are important for the binding and function of GLP-1 and exendin-4, though these residues also have roles in the biased agonism of these peptides (Wootten et al., 2013b, 2016). K197$^{2.67}$ sits below L201 and W297 in 3D space and is important for GLP-1 binding and activation (Coopman et al., 2010). R310$^{5.40}$ resides below N300 and also displays reduced potency in cAMP when mutated to alanine. Our model is also consistent with recent extensive studies on the GCGR (Siu et al., 2013) and CRF1R (Coin et al., 2013).

Despite the conservation of N-terminal sequence across all three peptides, oxyntomodulin appears to engage with the GLP-1R in a manner that is significantly different from that of exendin-4 and GLP-1. Although this ligand also requires large portions of ECL2 and, although to a lesser degree, the residues L201 and L384 deep in the extracellular surface of the protein, it does not require some key residues lining the entry to the cavity, including C296/W297 in ECL2 and R380 in ECL3, that are crucial for affinity of the other two peptides. The deeper membrane-proximal residues of ECL2 and ECL3 are also less important, and other residues such as L218 in ECL1 play no role in the affinity of oxyntomodulin, and this implies that oxyntomodulin, which has a lower affinity than GLP-1 and exendin-4, may not bind in the same manner as the other two peptides. This is supported by previous data that show only a limited role of residues at the bottom of the binding cavity (R190$^{2.60}$, N240$^{3.43}$, E364$^{6.53}$, and Q394$^{7.49}$) in oxyntomodulin affinity or cAMP formation (Wootten et al., 2013b, 2016). Indeed, a key predicted interaction in the GLP-1-GLP-1R model occurs between E^{9} (position 3) of the peptide and R190$^{2.60}$ of the receptor. However, oxyntomodulin contains a Q at this position that would not be expected to form a salt bridge with R190$^{2.60}$. Modified GLP-1 and oxyntomodulin peptides where the residue at position 3 is swapped

converts the behavior of these two peptides such that R190$^{2.60}$ is required for cAMP production by the modified oxyntomodulin but not for the modified GLP-1 (Figure S3). This provides strong evidence validating the positioning of the N-terminal segment of GLP-1 in our molecular model, and extended molecular dynamics (MD) simulation indicates that this interaction is stable (Figures S5E–S5H).

Although the extreme N terminus of GLP-1 is predicted to interact in the deep cavity within the TM bundle, MD simulations where the peptide N terminus is placed in a superficial position in a model of the open inactive receptor predict that the peptide ligand initially makes interactions with the extracellular surface of the GLP-1R prior to movement of the peptide deeper into the cavity driven by E^{9} (Figure S5). In the open conformation, ECLs 2 and 3 reside further apart in 3D space (Figures S5A–S5D), suggesting that there is also a reorganization of the ECLs in response to peptide binding with ECL2 and ECL3 moving closer together in 3D space in the activated, ligand-occupied receptor (Figures S5E–S5H). Mapping of mutational data (affinity and efficacy) onto this surface formed by ECL2 and ECL3 reveals a continuous surface illustrating that this 3D surface is critical for stabilization of peptide binding and for activation of downstream effectors (Figures 6 and 7, Active model S1). Taken together with the extensive crosslinking/cysteine-trapping studies on other class B GPCRs (Coin et al., 2013; Dong et al., 2012), this supports a role for both interactions of peptide ligands with the extracellular loops and deeper interactions within the TM bundle, which are both important for peptide binding, leading to propagation of signaling in class B GPCRs.

Importance of the ECL Regions of the GLP-1R for Efficacy

Overall, there was a very high correlation between residues important for peptide affinity and those linked to efficacy for cAMP formation and Ca^{2+} mobilization (Figures 2, 6, and 7). This is perhaps not surprising as both cAMP and Ca^{2+} mobilization are predominantly G protein-mediated pathways (Figure S1), and the ternary complex of the agonist-occupied receptor and effector (e.g., G protein) provides thermodynamically reciprocal regulation of agonist binding (De Lean et al., 1980). As such, the heatmaps of mutant effects on agonist affinity are a composite of direct effects on binding and those allosterically imposed via the effects of effector coupling, in particular, G protein coupling. For GLP-1 and exendin-4, unlike effects on affinity, almost the entire region of ECL2 is required for transmission of signal. Moreover, the contribution of individual residues varies between the different functional measures and the two ligands. Exendin-4 and GLP-1 display a similar efficacy for coupling to cAMP; however, exendin-4 is less efficient than GLP-1 at coupling to Ca^{2+} mobilization (Figure S2). Interestingly, the Ca^{2+} response mediated by exendin-4 is more sensitive to mutations within ECL2, ECL3, and TM2 membrane-proximal regions of ECL1 than that mediated by GLP-1, perhaps suggesting that subtle differences in the interactions formed by these ligands account for the small distinctions in signaling bias that are observed experimentally. These subtle differences in bias and the effect of mutations may be reflective of the nature of effector coupling that drives stimulation of individual signaling pathways. This is observed in inhibitor studies where relatively subtle differences were observed between GLP-1 and exendin-4, most notably in the relative contribution of $G_{\beta\gamma}$ subunits to pERK1/2 and iCa^{2+} signaling (Figure S1).

In addition to distinctions in the pattern of residues required for oxyntomodulin affinity compared to GLP-1 and exendin-4, there are also significant differences in the pattern of residues important for coupling the receptor to cAMP. Like the other two peptides, oxyntomodulin utilizes ECL2 and membrane-proximal regions of ECL3 for its function, and there is also evidence for the involvement of deeper residues in ECL2 and ECL3 that were not required for its affinity. However, in contrast to GLP-1 and exendin-4, there is a large involvement of residues in ECL1 and no requirement for residues in the proximal region of ECL2. Oxyntomodulin displays a very distinct signaling profile to GLP-1 (Figure S2), and collectively, the affinity and cAMP data support the notion that oxyntomodulin does not interact in the same manner, requiring a much larger portion of the extracellular surface to engender conformational transitions linking peptide interactions to signaling inside the cell. Furthermore, in contrast to GLP-1 and exendin-4, a component of the oxyntomodulin-mediated cAMP production is dependent on $G_{\beta\gamma}$ subunits, suggesting perhaps that a different subset of adenylate cyclases are activated to generate this cAMP response.

In addition to the network of interconnected residues along the extracellular surface of the receptor, a number of more distal residues were also identified to contribute to signaling efficacy. Studies of the GCGR have suggested that interactions between the receptor ECD and ECLs can occur and that these can influence conformational transitions required for signaling (Koth et al., 2012). It is likely that similar interactions also occur for the GLP-1R, and these may account for the observed effects of some residues distal to the interconnected networks. Extended MD simulation of the full-length receptor is consistent with the potential for such interactions to occur (Movies S1 and S2).

Interactions Determining Activation of Distinct Signaling Pathways and Promotion of Biased Signaling

Regardless of the ligand, ECL3 is essential for coupling the peptide-receptor interaction to pERK1/2, whereas ECL2 is critical for coupling of these interactions to cAMP and Ca^{2+}. Using inhibitors to disrupt various G protein- and β-arrestin-mediated signaling pathways revealed that whereas cAMP and Ca^{2+} mobilization are predominantly driven by G protein-mediated signaling, pERK1/2 is a composite of both G protein- and β-arrestin-driven events, with approximately 30%–60% (depending on the peptide) of the signal attributed to this latter mechanism (Figure S1). As the signaling events leading to pERK1/2 are partially independent of G proteins, this may explain why there is a very distinct region of the receptor required for signaling to this pathway in comparison to Ca^{2+} and cAMP signaling, which are predominantly G protein mediated.

In addition to heatmapping, the data from this study can also be mapped onto models depicting global importance of residues across sets of ligands or importance of residues for individual ligands. In this way, it is easy to visually observe distinct regions of

the receptor that can achieve biased agonism. In Figure 7, which summarizes the three measures of function with data for all three peptides, the differential importance of regions in the extracellular face for oxyntomodulin compared to GLP-1 and exendin-4 can be clearly observed. Oxyntomodulin is a highly biased ligand compared to GLP-1 (and exendin-4), with bias toward both pERK1/2 signaling and regulatory protein recruitment (including β-arrestins) (Figure S2), biases that may be linked given the greater contribution of β-arrestins to the pERK1/2 response of oxyntomodulin relative to the other two peptides (Figure S1). Therefore, it is not surprising that quite a different pattern of residues are required for transmission of signal for this peptide. Compared to GLP-1, exendin-4 only has very minor bias in its signaling profile for the pathways assessed in the current study, and therefore it is not necessarily surprising that the residues important at the extracellular face for each of these peptides to signal to various pathways are similar, even if (as the heatmapping suggests) the extent to which each of these residues contributes may be different.

Combining this study with previously published quantitative information of residues that also contribute to conformational changes associated with activation provides additional context to how these surface interactions link with intramembranous networks to differentially control signaling (Figure S6). This type of information allows us to begin to understand how initial peptide interactions at the extracellular surface engage with distinct networks of intramembranous residues to link extracellular binding to engagement of intracellular effectors.

Mapping mutational effects on efficacy onto molecular models may also provide a basis for rational design of biased peptide agonists. If the required cellular efficacy for translation to therapeutic success is known, then this provides an ability to design peptides that exploit signaling bias therapeutically. This hypothesis can be tested to some extent using the metabolite of GLP-1, GLP-1(9-36)NH_2. Whereas this ligand lacks the first two amino acids of GLP-1, including the N-terminal His^7 that is critical for affinity and activation of cAMP (Adelhorst et al., 1994), it retains mid-regions of the peptide that, in our molecular model, interact with ECL3. Although unable to activate the cAMP pathway, the metabolite can still promote pERK1/2 (Figure S7). This suggests that peptides that maintain interactions with ECL3 while altering/removing interactions deeper in the bundle and perhaps with ECL2 could bias ligands toward pERK1/2; however, it remains to be seen whether ECL3 is a common activation domain for this subclass of receptors to promote coupling to β-arrestin-mediated signaling pathways such as pERK1/2.

Biased agonism is likely to be a crucial element of the function of class B GPCRs, as many of them can be activated by multiple endogenous ligands and their receptors are capable of activating multiple intracellular signaling pathways. Therefore, understanding the molecular determinants linking ligand interactions to activation of distinct signaling pathways, in addition to the physiological benefit of activating individual pathways, could have significant ramifications for future drug development and may provide the potential to rationally design future drug therapies, and this is highlighted by the in vivo actions of the biased GLP-1 agonist, P5 (Zhang et al., 2015).

EXPERIMENTAL PROCEDURES

Molecular Biology

We used Quikchange (Stratagene) to introduce mutations into the GLP-1R cloned into the pEF5/Frt/V5-Dest vector.

Cell Culture

Stable FlpIn CHO cell lines were generated using Gateway technology. For all assays, cells were seeded in 96-well plates at a density of 30,000 cells per well.

Radioligand-Binding Assays

Whole-cell competition radioligand binding was performed using ^{125}I-exendin-4(9–39) as the tracer ligand and competing with increasing concentrations of unlabeled peptide ligands as described previously (Koole et al., 2010).

Cell-Surface Expression

Cell-surface expression was detected either by using a cell-surface ELISA to detect a double c-Myc epitope label incorporated with the N-terminal region of the GLP-1R constructs or by calculation of the Bmax in the radioligand-binding experiments (Koole et al., 2012; Wootten et al., 2016).

Signaling Assays

For cAMP assays, cells were stimulated for 30 min in the presence of the phosphodiesterase inhibitor IBMX and then lysed. For pERK1/2, cells were stimulated for 6 min (the peak of the response) before lysis. Detection of cAMP and pERK1/2 in the lysates was performed using Alphascreen technology as previously described (Koole et al., 2010). Ca^{2+} mobilization was detected using a Fluo-4-AM dye immediately after ligand addition with an excitation wavelength of 485 nM and an emission wavelength of 520 nM with values derived from the peak response.

Data Analysis

Concentration response data were analyzed using a three-parameter logistic equation to determine affinity, EC_{50}, and E_{max} values. Efficacy was calculated by applying the operational model of agonism:

$$Y = Bottom + \frac{E_m - Bottom}{1 + ((10^{logK_A}) + (10^{log[A]}))/(10^{(log\tau + log[A])})}$$

where *Bottom* represents the *y* value in the absence of ligand, E_m is maximal system stimulation, K_A is the agonist-receptor dissociation constant, [*A*] is the ligand concentration, and τ is the operational measure of efficacy in the system, which incorporates signaling efficacy and receptor density. Derived τ values were corrected to cell-surface expression (τ_c) measured by ELISA, and errors were propagated from both τ and cell-surface expression.

Molecular Modeling

Energy-based conformational modeling of the GLP-1R complex with GLP-1 was performed with Modeler 9.15, and peptide docking and energy optimization were guided by published experimental data (Tables S5 and S6), as previously described (Wootten et al., 2016).

Detailed procedures and analysis are reported in the Supplemental Experimental Procedures.

SUPPLEMENTAL INFORMATION

Supplemental Information includes Supplemental Experimental Procedures, seven figures, six tables, one data file, and two movies and can be found with this article online at http://dx.doi.org/10.1016/j.cell.2016.05.023.

AUTHOR CONTRIBUTIONS

Conceptualization, D.W., J.S., P.M.S.; Methodology, D.W., C.A.R., K.J.S., J.C.M., M.L., P.E.T., P.M.S.; Software, C.A.R., K.J.S., J.C.M.; Formal Analysis, D.W., C.K., A.C., P.M.S.; Investigation, D.W., C.A.R., K.J.S., J.C.M., C.K., E.E.S., K.P., M.L., J.S., R.S.; Writing – Original Draft, D.W., C.A.R., L.J.M., A.C., P.M.S.; Writing – Review and Editing, C.K., S.G.B.F.; Visualization,

D.W., P.M.S.; Supervision, D.W., C.A.R., P.E.T., P.M.S.; Project Administration, D.W., P.M.S.; Funding Acquisition, D.W., C.A.R., A.C., P.M.S.

ACKNOWLEDGMENTS

This work was supported by National Health and Medical Research Council of Australia (NHMRC) project grants (1061044 and 1065410) and NHMRC program grant (1055134); Biotechnology and Biological Sciences Research Council of the United Kingdom project grant (BB/M006883/1); and Medical Research Council (UK) Research Leader Fellowship (G1001812) (C.A.R.). D.W. is a NHMRC Career Development Fellow. P.M.S. is a NHMRC Principal Research Fellow, and A.C. is a NHMRC Senior Principal Research Fellow. C.K. is a NHMRC CJ Martin Postdoctoral Fellow.

Received: December 1, 2015
Revised: February 16, 2016
Accepted: May 3, 2016
Published: June 16, 2016

REFERENCES

Adelhorst, K., Hedegaard, B.B., Knudsen, L.B., and Kirk, O. (1994). Structure-activity studies of glucagon-like peptide-1. J. Biol. Chem. *269*, 6275–6278.

Baggio, L.L., and Drucker, D.J. (2007). Biology of incretins: GLP-1 and GIP. Gastroenterology *132*, 2131–2157.

Barwell, J., Conner, A., and Poyner, D.R. (2011). Extracellular loops 1 and 3 and their associated transmembrane regions of the calcitonin receptor-like receptor are needed for CGRP receptor function. Biochim. Biophys. Acta *1813*, 1906–1916.

Bergwitz, C., Jusseaume, S.A., Luck, M.D., Jüppner, H., and Gardella, T.J. (1997). Residues in the membrane-spanning and extracellular loop regions of the parathyroid hormone (PTH)-2 receptor determine signaling selectivity for PTH and PTH-related peptide. J. Biol. Chem. *272*, 28861–28868.

Black, J.W., and Leff, P. (1983). Operational models of pharmacological agonism. Proc. R. Soc. Lond. B Biol. Sci. *220*, 141–162.

Coin, I., Katritch, V., Sun, T., Xiang, Z., Siu, F.Y., Beyermann, M., Stevens, R.C., and Wang, L. (2013). Genetically encoded chemical probes in cells reveal the binding path of urocortin-I to CRF class B GPCR. Cell *155*, 1258–1269.

Coopman, K., Huang, Y., Johnston, N., Bradley, S.J., Wilkinson, G.F., and Willars, G.B. (2010). Comparative effects of the endogenous agonist glucagon-like peptide-1 (GLP-1)-(7-36) amide and the small-molecule ago-allosteric agent “compound 2” at the GLP-1 receptor. J. Pharmacol. Exp. Ther. *334*, 795–808.

Couvineau, A., and Laburthe, M. (2012). The family B1 GPCR: structural aspects and interaction with accessory proteins. Curr. Drug Targets *13*, 103–115.

De Lean, A., Stadel, J.M., and Lefkowitz, R.J. (1980). A ternary complex model explains the agonist-specific binding properties of the adenylate cyclase-coupled beta-adrenergic receptor. J. Biol. Chem. *255*, 7108–7117.

Dong, M., Xu, X., Ball, A.M., Makhoul, J.A., Lam, P.C., Pinon, D.I., Orry, A., Sexton, P.M., Abagyan, R., and Miller, L.J. (2012). Mapping spatial approximations between the amino terminus of secretin and each of the extracellular loops of its receptor using cysteine trapping. FASEB J. *26*, 5092–5105.

Fredriksson, R., Lagerström, M.C., Lundin, L.G., and Schiöth, H.B. (2003). The G-protein-coupled receptors in the human genome form five main families. Phylogenetic analysis, paralogon groups, and fingerprints. Mol. Pharmacol. *63*, 1256–1272.

Hollenstein, K., Kean, J., Bortolato, A., Cheng, R.K., Doré, A.S., Jazayeri, A., Cooke, R.M., Weir, M., and Marshall, F.H. (2013). Structure of class B GPCR corticotropin-releasing factor receptor 1. Nature *499*, 438–443.

Katritch, V., Cherezov, V., and Stevens, R.C. (2013). Structure-function of the G protein-coupled receptor superfamily. Annu. Rev. Pharmacol. Toxicol. *53*, 531–556.

Kenakin, T., and Christopoulos, A. (2013). Signalling bias in new drug discovery: detection, quantification and therapeutic impact. Nat. Rev. Drug Discov. *12*, 205–216.

Koole, C., Wootten, D., Simms, J., Valant, C., Sridhar, R., Woodman, O.L., Miller, L.J., Summers, R.J., Christopoulos, A., and Sexton, P.M. (2010). Allosteric ligands of the glucagon-like peptide 1 receptor (GLP-1R) differentially modulate endogenous and exogenous peptide responses in a pathway-selective manner: implications for drug screening. Mol. Pharmacol. *78*, 456–465.

Koole, C., Wootten, D., Simms, J., Miller, L.J., Christopoulos, A., and Sexton, P.M. (2012). Second extracellular loop of human glucagon-like peptide-1 receptor (GLP-1R) has a critical role in GLP-1 peptide binding and receptor activation. J. Biol. Chem. *287*, 3642–3658.

Koth, C.M., Murray, J.M., Mukund, S., Madjidi, A., Minn, A., Clarke, H.J., Wong, T., Chiang, V., Luis, E., Estevez, A., et al. (2012). Molecular basis for negative regulation of the glucagon receptor. Proc. Natl. Acad. Sci. USA *109*, 14393–14398.

Overington, J.P., Al-Lazikani, B., and Hopkins, A.L. (2006). How many drug targets are there? Nat. Rev. Drug Discov. *5*, 993–996.

Pal, K., Melcher, K., and Xu, H.E. (2012). Structure and mechanism for recognition of peptide hormones by class B G-protein-coupled receptors. Acta Pharmacol. Sin. *33*, 300–311.

Reid, T.S. (2013). Practical use of glucagon-like peptide-1 receptor agonist therapy in primary care. Clin. Diabetes *31*, 148–157.

Siu, F.Y., He, M., de Graaf, C., Han, G.W., Yang, D., Zhang, Z., Zhou, C., Xu, Q., Wacker, D., Joseph, J.S., et al. (2013). Structure of the human glucagon class B G-protein-coupled receptor. Nature *499*, 444–449.

Wootten, D., Savage, E.E., Willard, F.S., Bueno, A.B., Sloop, K.W., Christopoulos, A., and Sexton, P.M. (2013a). Differential activation and modulation of the glucagon-like peptide-1 receptor by small molecule ligands. Mol. Pharmacol. *83*, 822–834.

Wootten, D., Simms, J., Miller, L.J., Christopoulos, A., and Sexton, P.M. (2013b). Polar transmembrane interactions drive formation of ligand-specific and signal pathway-biased family B G protein-coupled receptor conformations. Proc. Natl. Acad. Sci. USA *110*, 5211–5216.

Wootten, D., Reynolds, C.A., Koole, C., Smith, K.J., Mobarec, J.C., Simms, J., Quon, T., Coudrat, T., Furness, S.G.B., Miller, L.J., et al. (2016). A hydrogen-bonded polar network in the core of the glucagon-like peptide-1 receptor is a fulcrum for biased agonism: Lessons from class B crystal structures. Mol. Pharmacol. *89*, 335–347.

Zhang, H., Sturchler, E., Zhu, J., Nieto, A., Cistrone, P.A., Xie, J., He, L., Yea, K., Jones, T., Turn, R., et al. (2015). Autocrine selection of a GLP-1R G-protein biased agonist with potent antidiabetic effects. Nat. Commun. *6*, 8918.

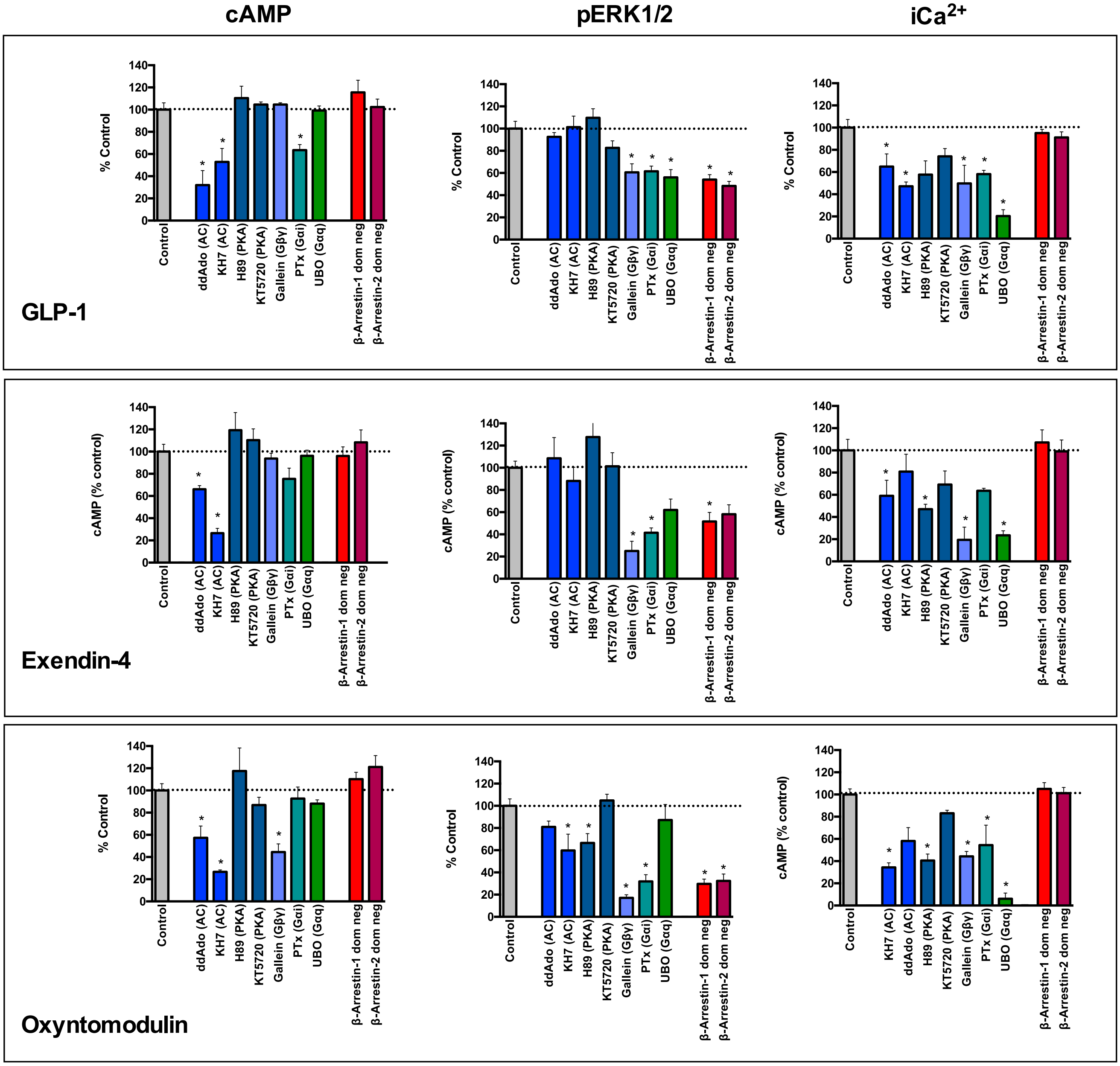

Figure S1. cAMP and Intracellular Calcium Mobilization Are G Protein-Mediated Signaling Pathways, whereas pERK1/2 Is a Composite of G Protein-Mediated and Non-G Protein-Mediated Events, Related to Figures 3, 4, 5, and 6

pEC_{50} concentrations of GLP-1 (top), exendin-4 (middle), and oxyntomodulin (bottom) were assessed for cAMP formation (left), calcium mobilization (right), and pERK1/2 (middle) in the absence (control) and presence of selective inhibitors or dominant-negative constructs. This included effectors downstream of Gαs (adenylate cyclase (AC), ddAdo, KH7) and protein kinase A (PKA), H89, KT5720), selective inhibitors of Gβγ, Gαi or Gαq (Gallein, Petussis toxin (PTx) and UBO respectively) and dominant negative versions of β-Arrestins 1 or 2. Data were normalized to % response in the absence of inhibitor/dominant-negative expression of β-arrestin to assess the importance of the various signaling effectors in peptide-mediated cAMP formation, calcium mobilization, and pERK1/2.

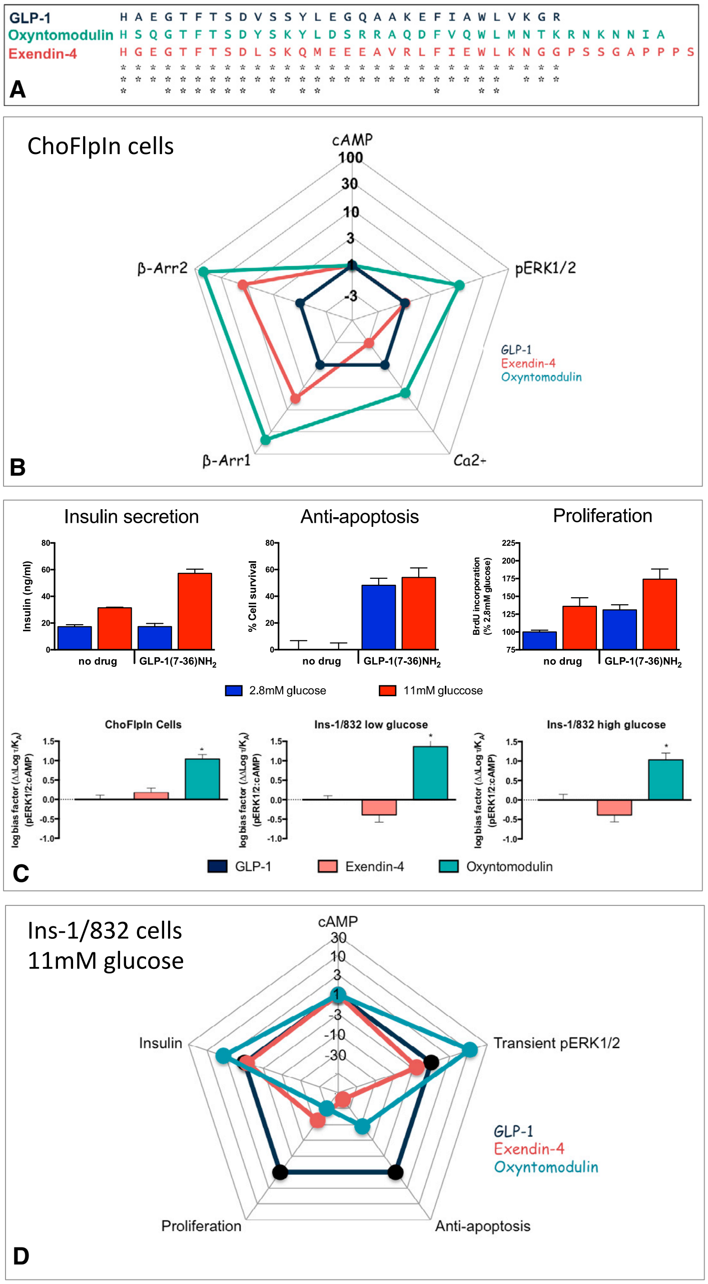

Figure S2. Assessment of Biased Agonism Reveals Distinctions in the Pattern of Signaling by GLP-1, Oxyntomodulin, and Exendin-4 at the GLP-1R in Both Recombinant and Natively Expressing Cells, Related to Figures 3, 4, 5, and 6

(A) Alignment showing the degree of sequence conservation between GLP-1, oxyntomodulin, and exendin-4.

(B and D) The "web of bias" plots $\Delta\Delta\tau/K_A$ values on a logarithmic scale for each ligand, for different signaling pathways in ChoFlpIn recombinant cells and Ins-1/832/3 insulinoma cells. Formation of these values included normalization to the reference ligand GLP-1 and the reference pathway, cAMP accumulation. Note, the plots do not provide information on absolute potency, but on relative efficacy for signaling of individual pathways to that for cAMP.

(C) Top panel: Assessment of insulin secretion, apoptosis and proliferation confirms that the Ins-1/832/3 cells are a suitable model for GLP-1R signaling beta islets with GLP-1 promoting glucose-dependent insulin secretion, in addition to promoting proliferation and decreasing apoptosis; these cells were used to reveal bias between GLP-1 and both exendin-4 and oxyntomodulin in insulin secretion, proliferation, and apoptosis (D). Bottom panel: comparison of biased agonism for cAMP promotion and pERK1/2 in ChoFLpIn cells (left), Ins-1/832/3 insulinoma cells in low glucose conditions (2.8 mM) (middle) and Ins-1/832/3 insulinoma cells in high glucose conditions (11 mM) (right). These data revel the biased profile of oxyntomodulin observed in ChoFlpIn cells overexpressing the GLP-1R translates to the natively expressing insulinoma cell line.

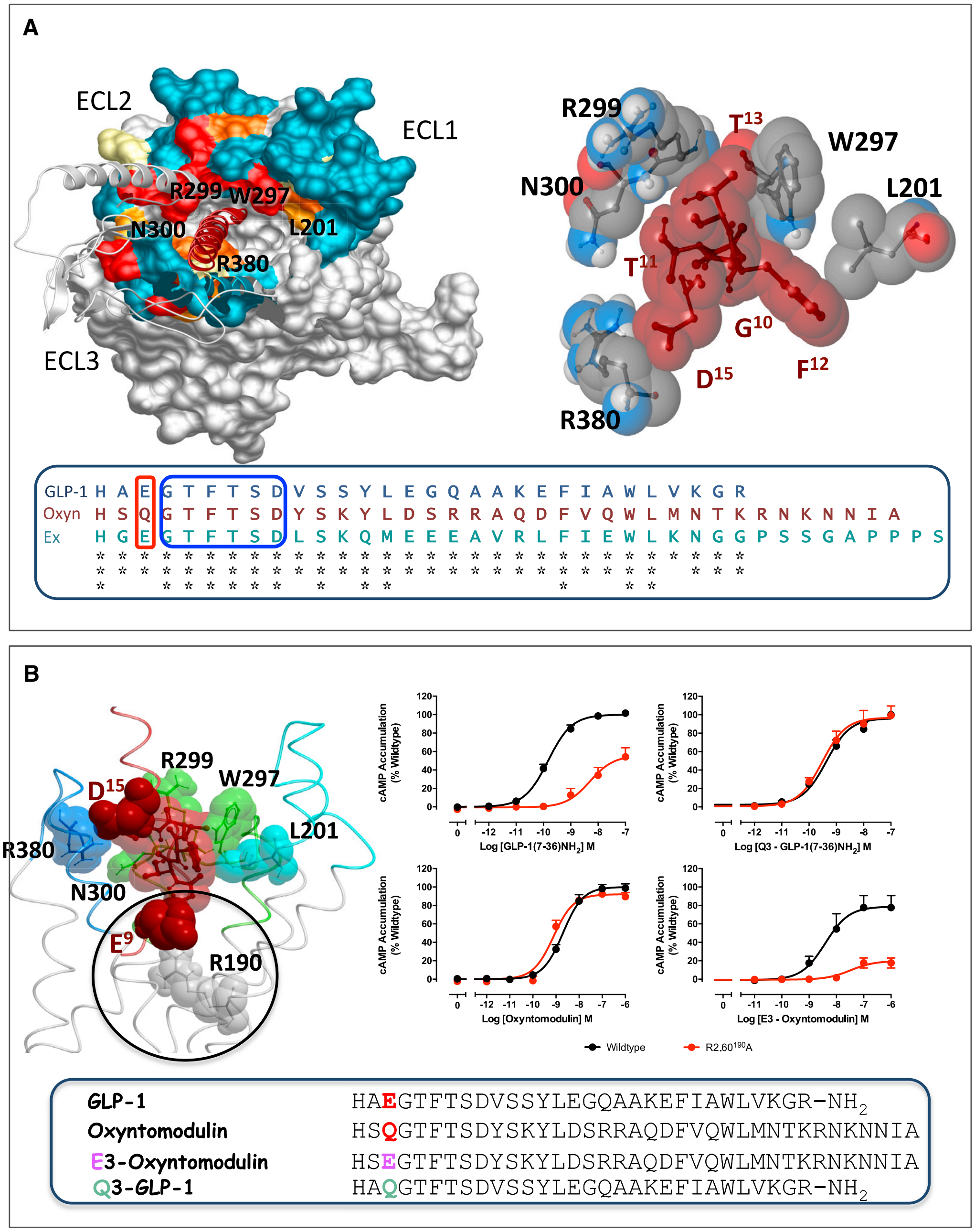

Figure S3. Direct Interactions of the GLP-1R ECL Residues and TM Bundle with GLP-1 Predicted from Molecular Modeling, Related to Figures 1 and 2

(A) Top left: Molecular model of GLP-1 (maroon) docked to the GLP-1R with key ECL residues labeled that line the entry to a deep cavity where the N terminus of GLP-1 is predicted to bind. Colors are the heatmaps from binding studies of GLP-1 from Figure 2. Top right: Close up of the peptide and ECL GLP-1R side chains that form direct interactions. Bottom: Sequence alignment of GLP-1, oxyntomodulin (Oxyn) and exendin-4 (Ex) highlighting the absolute sequence conservation within the region of the GLP-1 peptide (blue box) that is predicted to interact with the ECL residues, L201, W297, R299, N300, and R380 in the molecular model. Within the extreme N terminus of the three peptides, only position 3 (position 9 in the unprocessed GLP-1 peptide) differ significantly between GLP-1 and exendin (E) in comparison to oxyntomodulin (Q), as highlight by the red box.

(B) E^9 of GLP-1 (position 3) is predicted to interact with $R190^{2.60}$ within the TM bundle cavity. Q^3 of oxyntomodulin would not be predicted to form this sat bridge. In support of this, mutation of R190 to A markedly reduced cAMP signaling by GLP-1 (top left graph) but not oxyntomodulin (bottom left graph). To further validate the peptide docking in the GLP-1R model, substitution of E^9 with Q in the GLP-1 displayed a profile similar to that of oxyntomodulin at the R190A mutant receptor (top right graph). In contrast, substitution of Q^3 in oxyntomodulin with E produced a peptide with a similar profile to GLP-1 at R190A, with attenuated cAMP production compared to the wild-type receptor (bottom right graph).

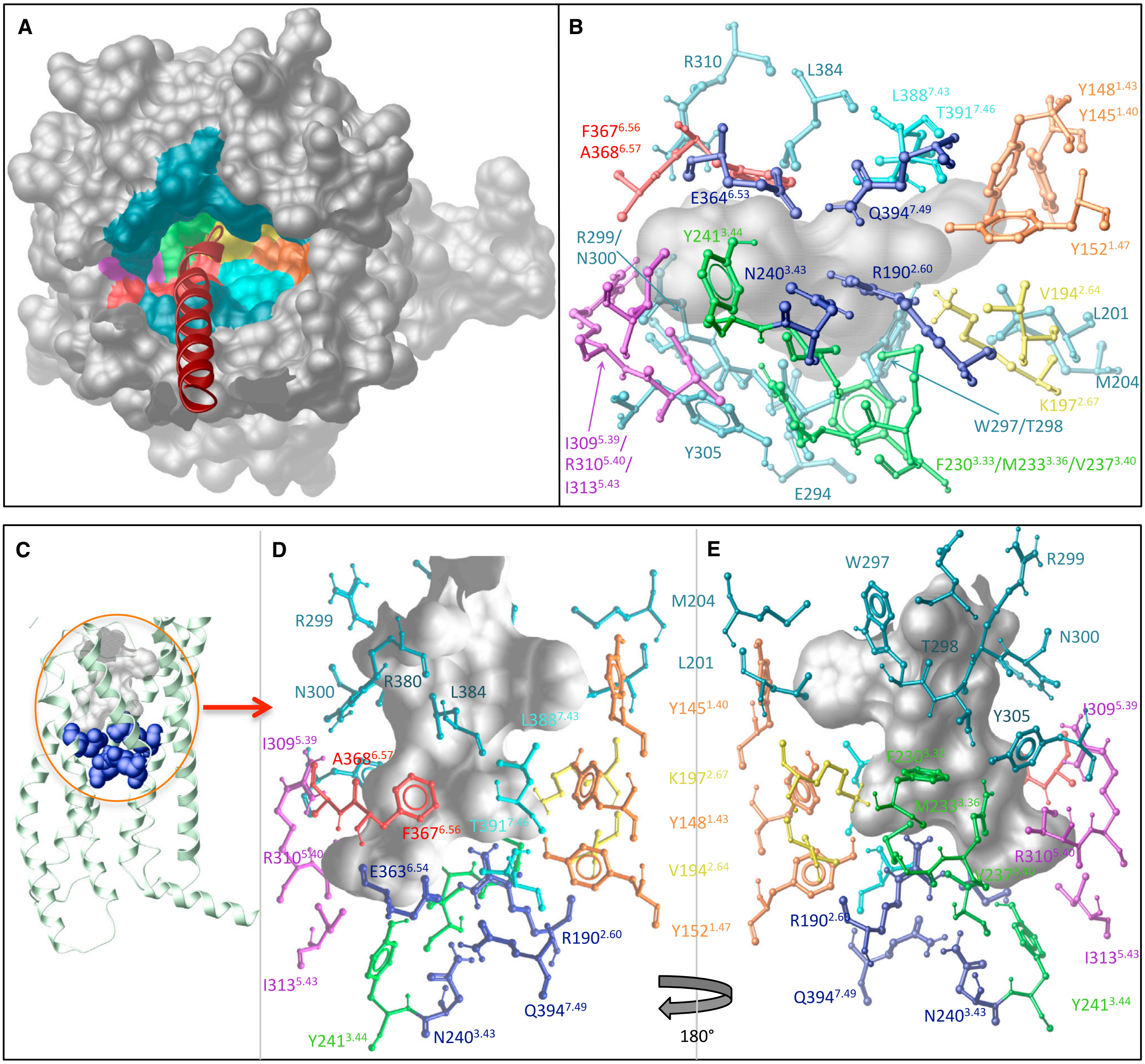

Figure S4. Modeling of the Predicted Peptide-Binding Cavity in the GLP-1R-GLP-1 Molecular Model, Related to Figures 1 and 3

Molecular model of the GLP-1R-GLP-1 complex reveals the N terminus of the peptide binds in a deep cavity within the TM domain of the GLP-1R. (A) Surface representation of the GLP-1R TM domain viewed from the extracellular face (N terminus removed) highlighting the deep cavity within the TM bundle. Residues lining this cavity are contributed from residues in TM1 (orange), TM2 (yellow), TM3 (green), TM5 (purple), TM6 (red), and TM7 (pale blue). Residues located in the ECLs that line the entry to this cavity are shown in teal. (B), (D), and (F) highlight all the residues provided from these TMs and ECLs that surround the peptide binding cavity shown from three different angles (B from beneath the cavity, D and E from the side of the cavity, 180 degree rotated in E compared to D). Four class B conserved polar residues that reside in a hydrogen bond network in the inactive conformation lining the bottom of this binding cavity are highlighted in dark blue (B–E), where the cavity is shown in gray. The remaining residues are colored according to their location in the TMs using the colors depicted in (A).

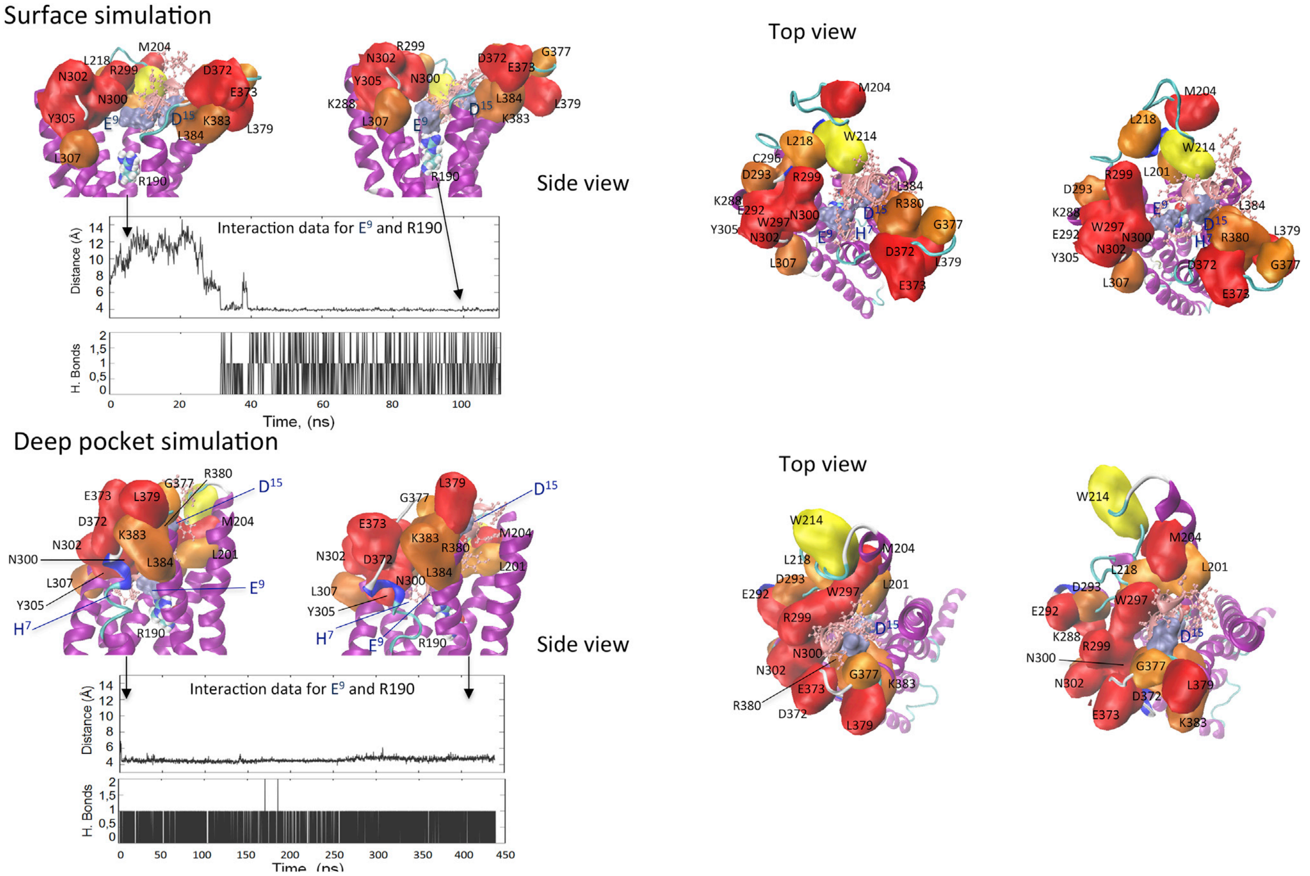

Figure S5. MD Simulations of Peptide Interaction with GLP-1 Receptor Models, Related to Figures 1 and 2

In the surface simulation (A–D), the N-terminal GLP-1 peptide fragment was placed such that D^{15} of the peptide was within 10 Å of R380 of the receptor, but no specific tethers were used. For this simulation, an open model of the receptor was used based on homology modeling using the CRF1 receptor crystal structure as initial template. The simulation was run for 220 ns. Interaction data for peptide residue E^9 and receptor residue R190 are shown in the inset panels. The upper inset displays the distance between C_δ (carbon delta) of E^9 of GLP1 and C_ζ (carbon zeta) of R190 of GLP-1R during the first 110 ns of the MD simulation. The E^9-R190 hydrogen-bonded salt-bridge engaged at ~t = 30 ns and then remained stable during the remainder of the 220 ns MD simulation. The lower panel of the inset displays hydrogen bonds between E^9 of GLP1 and R190 of GLP-1R during the first 110 ns of MD simulation. The donor–acceptor distance cutoff was 3.0 Å, and the angle cutoff was 20°. The E^9-R190 hydrogen-bonded salt-bridge remained stable during the remainder of the 220 ns MD simulation. Side views from the simulation are illustrated in (A) early time point, and (B) after ~100 ns where a stable interaction between the ligand and peptide had been formed. Panels (C) and (D) illustrate top views at each of the time points, respectively.

In the deep pocket simulation (E–H), MD was performed for a total of 500 ns, commencing with the final model of the full-length peptide bound receptor. The interaction between E^9 of the peptide and R190 of the receptor remained stable for the duration of the simulation. Inset panels display distances between C_δ (carbon delta) of E^9 of GLP1 and C_ζ (carbon zeta) of R190 of GLP-1R (upper inset) and hydrogen bonds between the two residues where donor–acceptor distance cutoff was 3.0 Å, and the angle cutoff was 20°. Panels (E) and (F) illustrate side views of early and late time points during the simulation. Panels (G) and (H) illustrate the equivalent top views, respectively.

The GLP-1 peptide fragment is displayed in pink (cpk) except for E^9 and D^{15} of the peptide (surface display, ice blue). The receptor is displayed in cartoon form, with the backbone colored by secondary structure. GLP-R residue 190 is displayed as VDW representation (colored by atom). GLP-1R residues that when mutated to alanine decreased GLP-1 peptide affinity are illustrated by quick surface, colored according to the fold-change in affinity (yellow, 3- to 5-fold; light orange, 5- to 10-fold; dark orange, 10- to 30-fold; red, >30-fold).

See Movies S1 and S2.

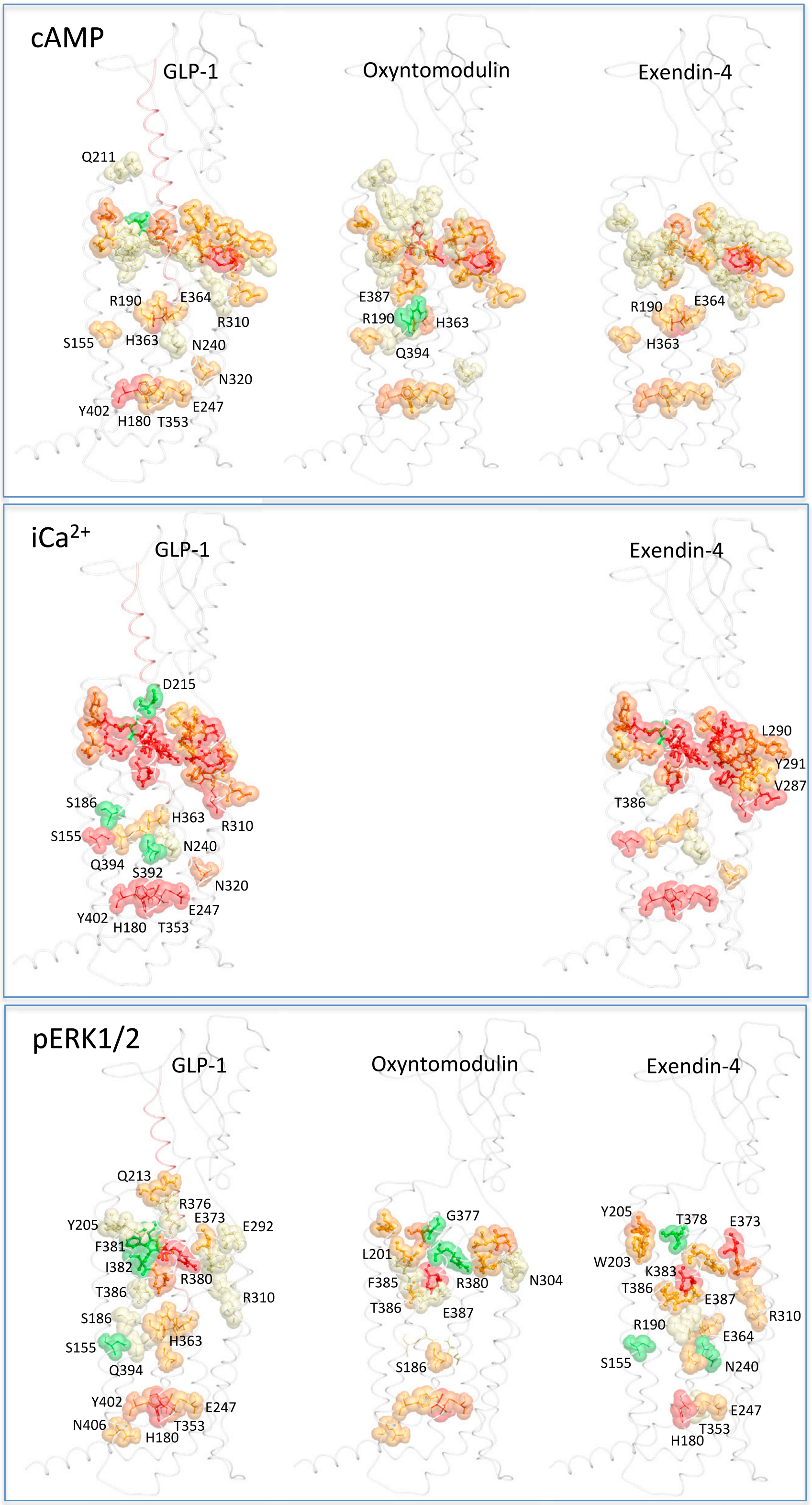

(legend on next page)

Figure S6. Signaling and Bias Agonism Triggered by Contacts with Peptide Ligands at the Extracellular Surface Is Propagated to the Intracellular Surface through Conformational Changes Involving Key Hydrogen Bond Networks, Related to Figures 2 and 6

Molecular model of full-length GLP-1R showing extended heatmaps highlighting the effects of Ala mutations to ECL loops residues in this study combined with polar residues reported in the literature that alter the efficacy (τ_c) of GLP-1 (left), oxyntomodulin (middle) and exendin-4 (right) for cAMP (A), iCa^{2+} (B), and pERK1/2 (C). Residues that statistically altered efficacy 3- to 5-fold are in yellow, 5- to 10-fold are pale orange, 10- to 30-fold are in dark orange, with those in red having greater than 30-fold loss in efficacy.

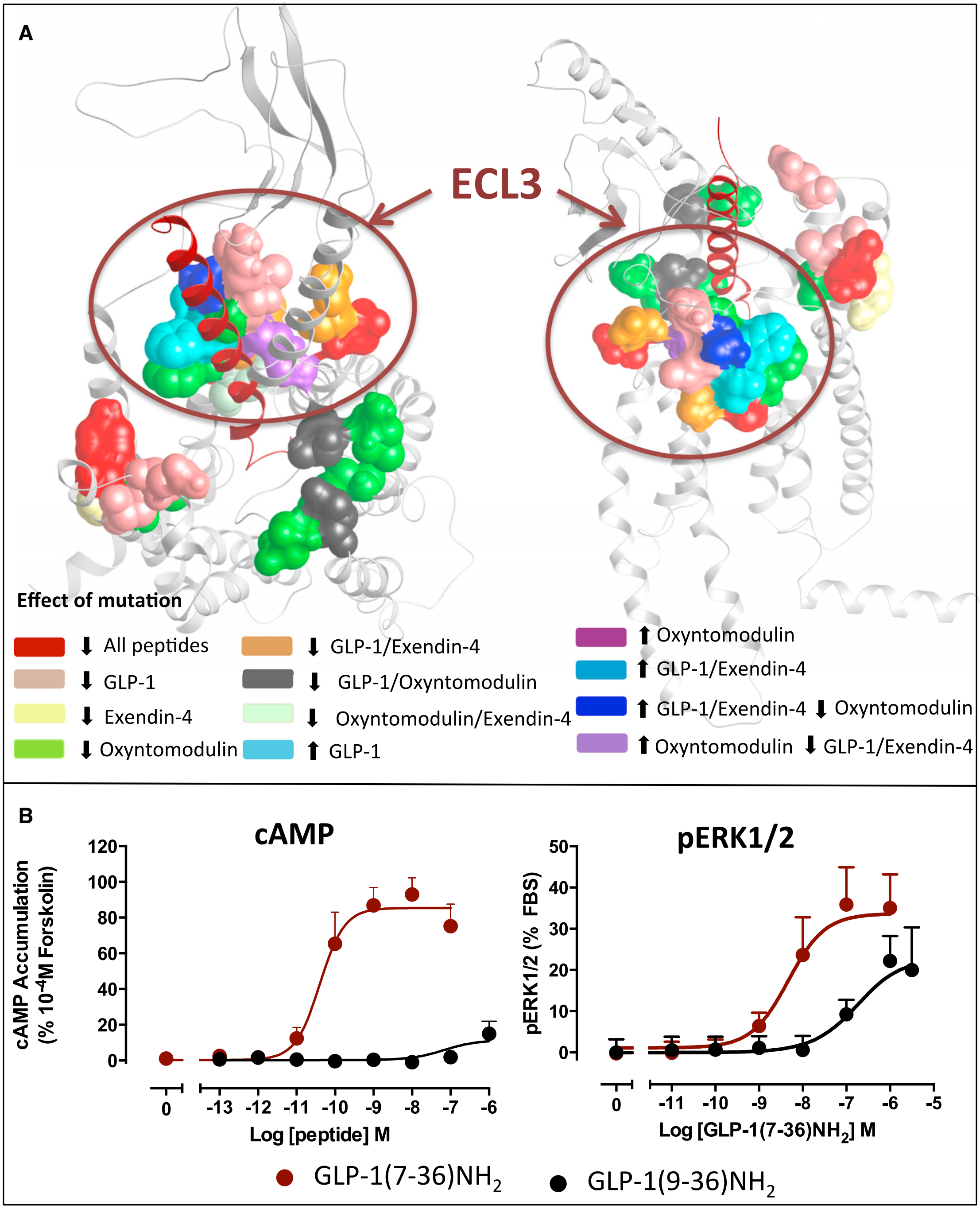

Figure S7. Residues within ECL3 Are Important for pERK1/2, Related to Figure 6

(A) Mapping of effects on pERK1/2 efficacy by GLP-1, oxyntomodulin, and exendin-4 when individual residues are mutated to alanine reveals a commonality in the principal site for signaling to this pathway that is localized to ECL3, although there are only a few residues within this site that, when mutated, have the same effect for all three peptides.

(B) Concentration response curves for cAMP accumulation (left) and pERK1/ (right) for GLP-1 (GLP-1(7-36)NH_2) and its truncated metabolite (GLP-1(9-36)NH_2). Data are normalized to the response elicited forskolin (cAMP) or FBS (pERK1/2) and fitted to a three parameter logistic equation (Equation 1 in SI).

All values are means ± SEM of three independent experiments, conducted in duplicate. These data reveal that removal of the first two residues of GLP-1 results in a peptide that is unable to generate a cAMP response but maintains the ability to promote pERK1/2.

Cell, Volume *165*

Supplemental Information

The Extracellular Surface of the GLP-1 Receptor

Is a Molecular Trigger for Biased Agonism

Denise Wootten, Christopher A. Reynolds, Kevin J. Smith, Juan C. Mobarec, Cassandra Koole, Emilia E. Savage, Kavita Pabreja, John Simms, Rohan Sridhar, Sebastian G.B. Furness, Mengjie Liu, Philip E. Thompson, Laurence J. Miller, Arthur Christopoulos, and Patrick M. Sexton

Supporting Information

Supplemental Experimental Procedures

Materials. GLP-1(7-36)NH_2, exendin-4 and oxyntomodulin were purchased from Mimotopes (Victoria, Australia). Dulbecco's Modified Eagle's Medium (DMEM) and Fluo-4 AM were purchased from Invitrogen (Carlsbad, CA, USA). Foetal bovine serum (FBS) was purchased from Thermo Electron Corporation (Melbourne, VIC, Australia). AlphaScreen reagents, 384-well opti and proxiplates were purchased from PerkinElmer Life and Analytical Sciences (Waltham, MA, USA). SureFire^{TM} ERK1/2 reagents were obtained from TGR Biosciences (Adelaide, SA, Australia). HRTF® insulin kit was from Cisbio. AlexaFluor® 488 annexin V conjugate (A13201) was from Molecular Probes. BrdU Cell Proliferation ELISA (chemiluminescent) kit was from Roche. N^{α}-Fmoc protected amino acids were purchased from Auspep, Chemimpex and Mimotopes. Rink amide resin (0.53 meq/g, 100-200 mesh), 2-chlorotrityl chloride resin (1.12 meq/g, 200-400 mesh) and HCTU were obtained from Chemimpex. TFA was purchased from Alfa Aesar. All other reagents were purchased from Sigma-Aldrich (St. Louis, MO, USA) or BDH Merck (Melbourne, Vic, Australia) and were of an analytical grade.

Peptide synthesis. Linear peptide chains (0.1 mmol scale) were synthesised on Rink amide resin [Q_3-GLP-1(7-36)NH_2] or 2-chlorotrityl chloride resin [E_3-oxyntomodulin] using a 3-channel serial automated peptide synthesiser ("PS3", Protein Technologies Inc.), which adopted standard Fmoc-based solid phase synthesis strategy. Fmoc deprotection was performed by 20% v/v piperidine in DMF for 2×5 min. Fmoc protected amino acids (3 *eq.*) were coupled using DMF as solvent, and DIPEA in DMF (7% v/v) with HCTU (3 *eq.*) as the activating agent for 50 min. Protected peptidyl-resins were cleaved by treating with TFA-TIPS-DMB (92.5%:2.5%:5%, for Rink amide resin) or TFA-TIPS (95%:5%, for 2-chlorotrityl chloride resin) for 2 h. The cleavage mixture was filtered, concentrated by a stream of N_2, precipitated in cold Et_2O and centrifuged at 3000 rpm for 5 min. The crude product was dissolved in water-acetonitrile mixture (50%:50%) and lyophilised, then purified by semi-preparative RP-HPLC. Peptides were purified on a Phenomenex Luna C-8 column (100Å, 10μm, 250×21.2mm) utilising a Waters 600 semi-preparative RP-HPLC that incorporates a Waters 486 UV detector. The wavelength was set at 230 nm. The eluting profile was a linear gradient of 0-80% acetonitrile in water buffered with 0.1% TFA over 60 min at 10 ml/min. ESI-MS was conducted using a Shimadzu LCMS2020 instrument, incorporating a Phenomenex Luna C-8 column (100Å, 3μm, 100×2.00mm). This system used 0.05% TFA in MilliQ water as the aqueous buffer, and 0.05% TFA in acetonitrile as the organic buffer. The eluting profile was a linear gradient of 0-60% acetonitrile in water over 10 minutes at 0.2 ml/min.

Receptor mutagenesis. To study the influence of ECL amino acids, and R190 on receptor function, the desired mutations were introduced to an N-terminally double c-myc labeled wild-type human GLP-1R in the pEF5/FRT/V5-DEST destination vector (Invitrogen); this receptor had equivalent pharmacology to the untagged human GLP-1R. Mutagenesis was carried out using oligonucleotides for site-directed mutagenesis purchased from GeneWorks (HindMarsh, SA, Australia) and the QuikChange^{TM} site-directed mutagenesis kit (Stratagene) and confirmed by automated sequencing (AGRF, Vic, Australia).

Cell culture: ***Stable transfections***; Wild-type and mutant human GLP-1Rs were isogenically integrated into FlpIn-Chinese hamster ovary (FlpInCHO) cells (Invitrogen) and selection of receptor-expressing cells accomplished by treatment with 600 μg ml^{-1} hygromycin-B as previously described (1). ***ChoFlpIn cell culture;*** All Transfected and parental FlpInCHO cells were maintained in DMEM supplemented with 10% heat-inactivated FBS and incubated in a humidified environment at 37°C in 5% CO_2. ***Ins-1 832/3 cell culture;*** Cells were maintained in RPMI 1640, supplemented with 10% v/v FBS, 1mM sodium pyruvate, 50μM 2-mercaptoethanol, 10mM HEPES, penicillin/streptomycin (50μg/mL) and 11mM glucose at 37°C in 5% CO_2.

Radioligand binding assay. FlpInCHO GLP-1R cells were seeded at a density of 3 x 10^4 cells/well into 96-well culture plates and incubated overnight at 37°C in 5% CO_2, and radioligand binding carried out as previously described (Koole et al., 2012). For each cell line in all experiments, total binding was defined by 0.05 nM ^{125}I-exendin-4(9-39) alone, and nonspecific binding was defined by 1 μM exendin-4(9-39). For analysis, data are normalised to the specific binding for each individual experiment.

Cell surface receptor expression. FlpInCHO WT and mutant human GLP-1R cells, with receptor DNA previously incorporated with an N-terminal double c-myc epitope label, were seeded at a density of 25 x 10^4 cells/well into 24-well culture plates and incubated overnight at 37°C in 5% CO_2, washed three times in 1 x PBS and fixed with 3.7% paraformaldehyde (PFA) at 4°C for 15 min. Cell surface receptor detection was then performed as previously described (Koole et al., 2012). Data were normalized to the basal fluorescence detected in FlpInCHO parental cells. Specific ^{125}I-exendin-4(9-39) binding at each receptor mutant, as identification of functional receptors at the cell surface, was also determined (corrected for nonspecific binding using 1 μM exendin-4(9-39)).

cAMP accumulation assay. FlpInCHO WT and mutant human GLP-1R cells were seeded at a density of 3 x 10^4 cells/well into 96-well culture plates and incubated overnight at 37°C in 5% CO_2. Peptide mediated cAMP accumulation assays were performed using the PerkinElmer AlphaScreen^{TM} kit as previously described (Koole et al., 2010). For Ins-1 832/3 cells were seeded at a density of 3 x 10^4 cells/well into laminin coated 96-well cell culture plates and were incubated for 48 h at 37°C in 5% CO_2. Cells were washed with 1X EBSS supplemented with 1.8 mM $CaCl_2$ and 2.8 mM glucose, pH 7.4 and media was replaced with RPMI containing 2.8mM glucose for 90 mins. Cells were washed and further incubated for 30 min in 135 μL of EBSS containing 1.8 mM $CaCl_2$, 2.8 mM glucose, 0.1% (w/v) BSA and 1 mM 3-isobutyl-1-methylxanthine (IBMX), pH 7.4, at 37°C in 5% CO_2. A. Cells were stimulated with the relevant concentrations of peptide for 30 min at 37°C in 5% CO_2 in the presence of low (2.8mM) or high (11mM) glucose. The reaction was terminated by rapid removal of the ligand containing buffer and addition of 100 μL of ice-cold 100% ethanol. cAMP was detected usinf the PerkinElmer AlphaScreen^{TM} kit as previously described for ChoFlpIn cells (Koole et al., 2010). All values were converted to concentration of cAMP using a cAMP standard curve performed in parallel, and data were subsequently normalized to the response of 100 μM forskolin.

ERK1/2 phosphorylation assay. FlpInCHO GLP-1R cells were seeded at a density of 3 x 10^4 cells/well into 96-well culture plates and incubated overnight at 37°C in 5% CO_2. Receptor-mediated pERK1/2 was determined using the AlphaScreen^{TM} ERK1/2 SureFire^{TM} protocol as previously described (Koole et al., 2010). For Ins-1 832/3 cells were seeded at a density of 3 x 10^4 cells/well into laminin coated 96-well cell culture plates and were incubated for 48 h at 37°C in 5% CO_2. Cells were washed with 1X EBSS supplemented with 1.8 mM $CaCl_2$ and 2.8 mM glucose, pH 7.4 and incubated for 5h in RPMI 1640 containing 2.8 mM glucose. Media was replaced with 135 μL 1X EBSS supplemented with 1.8 mM $CaCl_2$, 0.1% w/v BSA and 2.8 mM glucose, pH 7.4 and cells incubate for a further 1h. Cells were stimulated with the relevant concentrations of peptide ligand in either low (2.8mM) or high (11mM) glucose. Agonist stimulation of cells was terminated by the removal of buffer and the addition of 50 μL of SureFire lysis buffer to each well. Lysates were stored at -20°C. pERK1/2 was determined using the AlphaScreen^{TM} ERK1/2 SureFire^{TM} detection as previously described for ChoFlpIn cells (Koole et al., 2010).

Initial pERK1/2 time course experiments were performed over 1 h to determine the time at which agonist-mediated pERK1/2 was maximal for both the ChoFlpIn and Ins-1 832/3 cells. Subsequent experiments were then performed at the peak time required to generate a maximal pERK1/2 response. Note: None of the mutant receptors significantly altered the kinetic profile of the pERK1/2 response. Data were normalized to the maximal response elicited by 10% FBS determined at 6 min in both ChoFlpIn and Ins1-832 cells (peak FBS response).

Intracellular Ca^{2+} mobilisation assay. FlpInCHO GLP-1R cells were seeded at a density of 3 x 10^4 cells/well into 96-well culture plates and incubated overnight at 37°C in 5% CO_2, and receptor-mediated intracellular Ca^{2+} mobilisation determined as previously described (Koole et al., 2010). Fluorescence was determined immediately after ligand addition, with an excitation wavelength set to 485 nm and an emission wavelength set to 520 nm, and readings taken every 1.36 s for 120 s. Peak magnitude was calculated using five-point smoothing, followed by correction against basal fluorescence. The peak value was used to create concentration-response curves. None of the mutant receptors altered the kinetic profile of the calcium response. Data were normalized to the maximal response elicited by 100 μM ATP.

Insulin secretion. Ins-1 832/3 cells were seeded at 3x10^4 cells/well on laminin coated 96-well plates using RPMI 1640 complete media and incubated at 37°C with 5% CO_2 for 24h. Cells were washed and media replaced with RPMI-1640 supplemented with 2.8mM glucose and 2.5 % FBS and incubated for a further 24h. Prior to assay, the cells were washed twice with 1X EBSS supplemented with 2.8 mM

glucose, 1.8 mM $CaCl_2$ and 0.1% w/v BSA, pH 7.4. Peptide ligands were prepared at the relevant concentrations in 1X EBSS supplemented 1.8 mM $CaCl_2$ and 0.1% w/v BSA, pH 7.4 and with with either 2.8mM and 11 mM glucose and added directly to the cells after washing. After 2h, the supernatants were collected and insulin levels were detected using Cisbio HRTF® insulin kit following the manufacturers recommendations.

Apoptosis assay. Ins-1 832/3 cells plated were plated on to 6-well plates at a density of 5×10^5 cells/well using pre-warmed RPMI 1640 complete media and incubate at 37°C in 5% CO_2 for 48h. Media was replaced with RPMI 1640 supplemented with 2.8 mM and 2.5% FBS and incubated for a further 24h. Cells were washed twice with 1X EBSS supplemented with 2.8 mM glucose and 1.8 mM $CaCl_2$, pH 7.4. Subsequently, peptides prepared at the relevant concentrations in the presence and absence of 100 nM staurosporine in RPMI 1640 complete media with either 2.8mM or 11mM glucose were added to the cells (2mL). Cells were incubated for a further 24 h at 37°C with 5% CO_2. Following incubation supernatant was collected and remaining cells were harvested and pelleted at 350g for 3 min. The supernatant was discarded and cells were resuspended in HBSS, pH 7.4. Apoptotic cells were identified using AlexaFluor® 488 annexin V conjugate (1:10 dilution, A13201, Molecular Probes ®) and PI (1mg/mL in water). Apoptotic cells (annexin and PI positive) were identified using a BD FACS Canto™ II flow cytometer measuring the fluorescence at 530 nm / 30 nm (band pass) and 585 nm / 42 nm using 488 nm excitation.

Proliferation assay. Ins-1 832/3 cells were seeded at 1.2×10^4 cells/well in laminin coated, 96-well, clear bottomed, black view plates in pre-warmed culture media and incubated for 24h at 37°C in 5% CO_2. Media was replaced with RPMI 1640 supplemented with 2.8mM glucose and incubated for a further 12h. Cells were washed and media replaced with 200μL RPMI 1640 containing 1% FBS, 0.2% BSA, 2mM sodium pyruvate, 100 μM β-mercaptoethanol, 20 mM HEPES and either low glucose (2.8mM) or high glucose (11mM), containing the relevant concentrations of peptide ligands. Media with 10% FBS was used at a positive control. Plates were incubated at 37°C in 5% CO_2 for 24h. Media was removed from the wells and proliferation was assessed by BrdU incorporation using the Cell Proliferation ELISA, BrdU (chemiluminescent) kit from Roche, following the manufacturers instructions. Luminescence was detected using the LumiSTAR Omega instrument (BMG LABTECH GmbH, Ortenberg, Germany).

Molecular modelling. An active model of the GLP1 human receptor (residues 27-421) containing both GLP-1(7-36)NH_2 and the C-terminal peptide of Gs was constructed from a number of overlapping structural templates using Modeller 9.15 (Eswar et al., 2007). These templates included the glucagon receptor TM X-ray structure (pdb code 4L6R (Siu et al., 2013)), the extracellular domain containing GLP-1(10-35) (pdb code 3IOL (Underwood et al., 2010)) and two fragments taken from the β_2-adrenergic receptor:G-protein active complex (pdb code 3SN6 (Rasmussen et al., 2011)), namely helices 5 and 6 (the biggest structural difference reside in TM6) and an intracellular portion containing the lower part of the TM domain. Both fragments contained the Gs C-terminal peptide (R373-L394); these fragments were mutated to their GLP-1R equivalent (Taddese et al., 2014) using Modeller. In addition, the CRF-1R X-ray structure (pdb code 4K5Y (Hollenstein et al., 2013)) was used to generate an ECL1 helix-containing template as 4L6R lacks a full ECL1. The NMR structure of an active, conformationally-constrained version of GLP-1(7-17) (pdb code 2N0I (Hoang et al., 2015)), with activity similar to GLP-1(7-36)NH_2 was used to model the N-terminal part of the peptide.

All 15 members of the 2N0I ensemble were mutated to GLP-1(7-17)-NH_2 and minimized using PLOP (Jacobson et al., 2004). They were then docked using GLIDE SP peptide (Friesner et al., 2004; Tubert-Brohman et al., 2013) and the recent OPLS3 force field, with the backbone conformation held rigid, to reflect the biologically active conformation, to a GLP-1R model derived from the glucagon X-ray structure (Siu et al., 2013) that had been primed by Modeller (Eswar et al., 2007) to bind GLP-1(7-36)NH_2. The top scoring pose was for model 7, which belongs to the second cluster of the NMR ensemble, as determined by NMRclust (Kelley et al., 1997; Kelley and Sutcliffe, 1997), and was the only pose to form two good salt-bridge interactions (Table S5). The alpha helical residues T13-S17 of GLP-1(7-17) were structurally aligned to the corresponding residues of GLP-1(10-35) of the 3IOL structure using VMD (Humphrey et al., 1996). The overlap of GLP-1(7-17), as docked to the 7TM structure and structurally aligned to the ECD, is a key feature in enabling Modeller to generate a full GLP-1R structure; these structures were used in addition to the 4L6R and 3IOL templates.

The sequences of the templates were aligned to the target sequence of GLP-1(7-36)NH_2 appended to that of GLP-1R. Six key constrains were used by Modeller in generating the model from the alignment and the template structures, namely the photoaffinity distance constraints between GLP-1R and BPA placed at 4 different peptide positions(Chen et al., 2009; Chen et al., 2010; Miller et al., 2011) and two salt-bridges between conserved residues in GLP-1R or related receptors, namely between E^9 of the peptide and TM2 and D^{15} and TM7 (Moon et al., 2015; Solano et al., 2001; Vertongen et al., 2001) (see Table S5). The effect of these 6 constraints is to supplement the quality of the model at the interface between the two templates using the best information currently available. Two thousand models were generated using Modeller, and the structure with the lowest (best) DOPE score (Shen and Sali, 2006) was selected. This model was refined in the presence of an α-helical ECL1 template derived from the CRF-R1 structure (pdb code 4K5Y (Hollenstein et al., 2013)). The α-helical portion (Q213-S219) and its orientation was determined by matching the variability (Baldwin et al., 1997; Vohra et al., 2013) of GLP-1R sequences (taken from the GPCRDB (Horn et al., 2003)) with the corresponding variability from CRF-R1 sequences; the orientation was maintained during model determination using constraints on 5 ECL1 and 5 peptide residues (Table S6). In this second phase, 5000 structures were generated using Modeller and the structure with the lowest DOPE score was selected. The structure is available from ftp://ftp.essex.ac.uk/pub/oyster/Wootten_Cell_2016/ (username: ftp, password: anonymous). The heat maps were plotted using icm (Molsoft) and these are also presented in the Supplementary file Active_movie_S1.icb that can be viewed on the free ICM browser (available from http://www.molsoft.com/icm_browser.html).

Molecular dynamics simulations. ***Surface simulation (Movie S1)***; The TM domain of GLP-1R was modeled based on the CRF1 receptor crystal structure (Hollenstein et al., 2013) using the homology modelling functionality of PLOP (Jacobson et al., 2004); the CRF1R template was chosen to give a more open structure. The N-terminus segment of GLP-1 was positioned on the surface of the extracellular loops with the help of the Maestro (Schrödinger LLC) graphical user interface. This GLP1-GLP1 complex was embedded in a lipidic POPC bilayer with explicit water and ions and simulated using ACEMD (Harvey et al., 2009), with the AMBER 14SB (Hornak et al., 2006) and lipid 14 force fields (Walker et al., 2014) for 220 ns at 300 K and 1 atmosphere pressure. The initial dimensions were 85.1 Å × 85.45 Å× 86.77 Å. ***Deep pocket simulation (Movie S2)***; The full GLP-1R in complex with GLP-1 (see modeling section) with the N-terminus of GLP-1 located deep into the TM domain, was embedded in a lipid POPC bilayer with water and ions and simulated as described for the surface simulations for 500 ns. The initial dimensions were 88.7 Å × 89.1 Å× 134.17 Å.

Data analysis. All experimental data were analysed using Prism 6 (GraphPad Software Inc., San Diego, CA, USA). For all analyses the data are unweighted and each y value (mean of replicates for each individual experiment) is considered an individual point Concentration response signalling data were analysed using a three-parameter logistic equation as previously described:

$$Y = Bottom + \frac{(Top - Bottom)}{1 + 10^{(LogEC_{50} - log[A])}} \qquad (1)$$

where *Bottom* represents the y value in the absence of ligand(s), *Top* represents the maximal stimulation in the presence of ligand(s), [A] is the molar concentration of ligand, and EC_{50} represents the molar concentration of ligand required to generate a response halfway between Top and Bottom. Similarly, this equation was used in the analysis of inhibition binding data, instead replacing EC_{50} with IC_{50}. In this case, *Bottom* defines the specific binding of the radioligand that is equivalent to non-specific ligand binding, whereas *Top* defines radioligand binding in the absence of a competing ligand, and the IC_{50} value represents the molar concentration of ligand required to generate a response halfway between Top and Bottom. IC_{50} values obtained were then corrected for radioligand occupancy as previously described using the radioligand affinity (K_i) experimentally determined for each mutant.

To quantify efficacy in the system, all data were fitted with an operational model of agonism:

$$Y = Bottom + \frac{E_m - Bottom}{1 + ((10^{logK_A}) + (10^{log[A]}))/(10^{(log\tau + log[A])})} \qquad (2)$$

where *Bottom* represents the y value in the absence of ligand(s), E_m represents the maximal stimulation of the system, K_A is the agonist-receptor dissociation constant, in molar concentration, $[A]$ is the molar concentration of ligand and τ is the operational measure of efficacy in the system, which incorporates signaling efficacy and receptor density. Constraints for this model were determined by fitting the operational model for a partial agonist to each of the peptides at the wild-type receptor, with the most efficacious peptide fitted with:

$$Y = Bottom + \frac{E_m - Bottom}{1 + 10^{(LogEC_{50} - log[A])}} \qquad (3)$$

and the less efficacious peptides fitted with equation (2), in order to obtain a value for the system maximum (E_m). This value was then globally constrained in the operational model (equation (2)). All estimated τ values were then corrected to cell surface expression (τ_c) as determined by cell surface ELISA and errors propagated from both τ and cell surface expression.

Bmax from homologous competition was determined using equation 4 to radioligand binding data (^{125}I-exendin(9-39) binding by unlabelled exendin(9-39)), where Bmax is the maximum binding of ligand to receptors, [Hot] is the concentration of ^{125}I-exendin(9-39) in nM, [Cold] is the concentration of unlabelled exendin(9-39) in nM, and Kd is the equilibrium dissociation constant of the ligand in nM, with *Bottom* as defined in equation 1.

$$Y = \frac{Bmax\ x\ [Hot]}{[Hot]+[Cold]+Kd} + Bottom \qquad (4)$$

Statistics. All data are represented as mean ± SEM and were compared using ANOVA followed by Dunnett's test. Repeated measures analysis of variance was used to assess the statistical significance between time courses. The null hypothesis was rejected at $P<0.05$.

Table S1. ***Related to Figure 2.*** **Effects of human GLP-1R ECL1 alanine mutants on peptide ligand binding and cell surface expression.** Binding data were analyzed using a three parameter logistic equation as defined in eq 1 in SI. pIC_{50} values represent the negative logarithm of the concentration of ligand that inhibits binding of half the total concentration of radiolabelled antagonist, ^{125}I-exendin(9-39). Data are normalized to maximum ^{125}I-exendin(9-39) binding in the absence of ligand, with non-specific binding measured in the presence of 1 μM exendin(9-39). All values are expressed as mean ± S.E.M. of three to four independent experiments, conducted in duplicate. Cell surface expression was determined through antibody detection of the N-terminal c-myc epitope label and expressed as percentage of wildtype human GLP-1R expression. All values are mean ± S.E.M. of five to six individual experiments. Bmax values were determined through homologous whole cell competition binding. Data is expressed as percentage wildtype human GLP-1R Bmax, with all values expressed as mean ± S.E.M. of three to four independent experiments performed in duplicate Data were analyzed with one-way analysis of variance and Dunnett's post test.

		Whole cell competition radioligand binding (pKi)				Cell surface expression (% wildtype)	Whole cell binding (Bmax) (% wildtype)
		GLP-1(7-36)NH_2	Oxyntomodulin	Exendin-4	Exendin(9-39)		
	Wildtype	8.76 ± 0.04	7.66 ± 0.06	9.11 ± 0.07	7.96 ± 0.04	100 ± 2	100 ± 1
**ECL1**	L201A	8.05 ± 0.13*	6.74 ± 0.14*	8.83 ± 0.13	7.66 ± 0.10	94 ± 2	94 ± 11
	K202A	8.42 ± 0.11	6.77 ± 0.07*	8.88 ± 0.11	7.59 ± 0.09	94 ± 3	110 ± 9
	W203A	8.52 ± 0.06	7.50 ± 0.05	9.00 ± 0.04	7.89 ± 0.05	94 ± 6	104 ± 3
	M204A	7.09 ± 0.18*	6.21 ± 0.24*	7.98 ± 0.07*	7.78 ± 0.84	99 ± 2	100 ± 16
	Y205A	8.25 ± 0.11	7.33 ± 0.08	8.18 ± 0.03	7.90 ± 0.09	68 ± 5*	58 ± 6*
	S206A	8.64 ± 0.10	7.76 ± 0.08	8.83 ± 0.07	8.12 ± 0.09	111 ± 6	112 ± 9
	T207A	8.67 ± 0.06	7.65 ± 0.15	8.85 ± 0.09	7.89 ± 0.10	75 ± 6*	59 ± 5*
	Q210A	8.64 ± 0.08	7.02 ± 0.12	8.81 ± 0.08	7.93 ± 0.06	88 ± 4	87 ± 11
	Q211A	8.66 ± 0.08	7.63 ± 0.10	8.73 ± 0.14	8.05 ± 0.03	112 ± 5	105 ± 6
	H212A	8.81 ± 0.07	7.84 ± 0.10	9.43 ± 0.10	7.96 ± 0.05	103 ± 3	72 ± 9
	Q213A	8.88 ± 0.10	7.74 ± 0.04	8.72 ± 0.06	7.96 ± 0.07	101 ± 2	104 ± 3
	W214A	8.20 ± 0.07*	7.44 ± 0.12	8.90 ± 0.09	7.78 ± 0.12	99 ± 5	108 ± 4
	D215A	8.34 ± 0.04	7.50 ± 0.12	*8.92* ± 0.13	7.90 ± 0.02	90 ± 2	85 ± 9
	L217A	8.72 ± 0.07	7.47 ± 0.08	8.69 ± 0.09	7.91 ± 0.06	109 ± 5	97 ± 9
	L218A	7.91 ± 0.08*	7.15 ± 0.10	8.14 ± 0.08*	7.64 ± 0.07	89 ± 6	98 ± 8
	S219A	8.82 ± 0.03	7.79 ± 0.06	9.05 ± 0.06	8.23 ± 0.09	104 ± 3	93 ± 9
	Y220A	8.49 ± 0.07	7.33 ± 0.10	9.23 ± 0.09	7.88 ± 0.07	94 ± 4	73 ± 5
	Q221A	8.53 ± 0.05	7.81 ± 0.08	8.91 ±0.05	7.69 ± 0.09	97 ± 3	87 ± 4
	D222A	8.62 ± 0.07	7.35 ± 0.04	8.95 ± 0.05	7.99 ± 0.04	102 ± 4	99 ± 17

	S223A	8.32 ± 0.07	7.32 ± 0.07	8.68 ± 0.06	8.07 ± 0.08	90 ± 5	100 ± 1
	Wildtype	8.75 ± 0.06	7.30 ± 0.07	9.22 ± 0.04	8.22 ± 0.04	100 ± 3	100 ± 1
**ECL3**	D372A	7.15 ± 0.08*	6.81 ± 0.26	8.12 ± 0.06*	7.63 ± 0.09	86 ± 4	92 ± 5
	E373A	7.08 ± 0.09*	6.43 ± 0.17*	7.95 ± 0.02*	8.15 ± 0.07	94 ± 7	99 ± 8
	H374A	8.59 ± 0.08	7.08 ± 0.16	9.10 ± 0.07	7.35 ± 0.12*	82 ± 8	89 ± 3
	R376A	8.15 ± 0.13	7.14 ± 0.13	8.90 ± 0.06	7.79 ± 0.11	92 ± 7	96 ± 6
	G377A	7.85 ± 0.06*	6.89 ± 0.10	8.79 ± 0.12	8.18 ± 0.09	96 ± 3	103 ± 4
	T378A	8.79 ± 0.09	6.67 ± 0.13	8.71 ± 0.05*	8.10 ± 0.07	173 ± 11*	153 ± 8*
	L379A	7.08 ± 0.05*	6.45 ± 0.20*	8.47 ± 0.11*	8.30 ± 0.05	90 ± 6	97 ± 3
	R380A	7.65 ± 0.13*	6.71 ± 0.29	7.75 ± 0.07*	8.03 ± 0.07	73 ± 5*	76 ± 7*
	F381A	8.42 ± 0.04	6.18 ± 0.16*	8.97 ± 0.06	6.86 ± 0.10*	92 ± 5	95 ± 1
	I382A	8.82 ± 0.10	7.37 ± 0.12	9.10 ± 0.03	8.18 ± 0.16	115 ± 6	109 ± 6
	K383A	7.76 ± 0.14*	6.40 ± 0.34*	8.17 ± 0.13*	7.29 ± 0.09*	78 ± 4*	81 ± 1*
	L384A	7.69 ± 0.12*	6.47 ± 0.30*	8.06 ± 0.08*	7.89 ± 0.15	74 ± 7*	83 ± 3
	F385A	8.97 ± 0.07	7.17 ± 0.07	9.00 ± 0.09	8.29 ± 0.09	110 ± 7	106 ± 5
	T386A	8.41 ± 0.08	7.23 ± 0.14	8.66 ± 0.05*	8.22 ± 0.07	128 ± 6	108 ± 5
	E387A	8.55 ± 0.07	6.86 ± 0.09	8.83 ± 0.08	8.03 ± 0.06	104 ± 6	101 ± 4

* Statistically significant at $p < 0.05$, one way ANOVA and Dunnett's post test in comparison to wildtype performed within the same dataset (ECL1 or ECL3).

Table S2. ***Related to Figure 4.*** **Effects of human GLP-1R ECL1 and ECL3 mutants on peptide agonist-induced cAMP accumulation.** Data were analyzed using an operational model of agonism as defined in eq 1 in SI. pEC$_{50}$ values represent the negative logarithm of the concentration of agonist that produces half the maximal response. E$_{max}$ E$_{max}$ are presented as a % of the WT response. Logτ values represent the intrinsic efficacy. All Logτ values were corrected to the cell surface expression data as determined by the ELISA (Logτ_c) All values are expressed as mean ± S.E.M. of four to six individual experiments, performed in duplicate. Data were analyzed with one-way analysis of variance and Dunnett's post test.

		Agonist-mediated cAMP Accumulation								
		GLP-1(7-36)NH$_2$			Oxyntomodulin			Exendin		
		pEC$_{50}$	E$_{max}$	Logτ_c (τ_c)	pEC$_{50}$	E$_{max}$	Logτ_c (τ_c)	pEC$_{50}$	E$_{max}$	Logτ_c (τ_c)
	Wildtype	9.66 ± 0.05	100 ± 1	1.02 ± 0.04 (10.5)	8.70 ± 0.06	100 ± 2	0.83 ± 0.05 (6.7)	10.45 ± 0.07	100 ± 2	1.03 ± 0.05 (10.7)
**ECL1**	L201A	8.47 ± 0.11*	92 ± 3	0.65 ± 0.05 (4.5)*	7.43 ± 0.15*	61 ± 4*	0.10 ± 0.19 (1.3)*	9.30 ± 0.12*	100 ± 3	0.51 ± 0.07 (3.2)*
	K202A	8.39 ± 0.08*	97 ± 2	0.37 ± 0.04 (2.4)*	8.14 ± 0.06*	98 ± 2	0.54 ± 0.10 (3.5)	9.80 ± 0.09*	104 ± 2	0.61 ± 0.05 (4.1)
	W203A	9.13 ± 0.06*	96 ± 2	0.63 ± 0.06 (4.3)	8.81 ± 0.11	90 ± 6	0.57 ± 0.05 (3.7)	10.43 ± 0.07	94 ± 2	0.88 ± 0.08 (7.6)
	M204A	7.93 ± 0.05*	105 ± 2	0.90 ± 0.07 (7.9)	7.58 ± 0.06*	100 ± 5	0.69 ± 0.17 (4.9)	9.71 ± 0.07*	107 ± 2	1.19 ± 0.07 (15.5)
	Y205A	8.90 ± 0.27*	30 ± 3*	-0.21 ± 0.09 (0.6)*	7.90 ± 0.20*	40 ± 8*	0.23 ± 0.06 (1.7)*	9.32 ± 0.25*	47 ± 8*	0.56 ± 0.08 (3.6)*
	S206A	9.78 ± 0.10	112 ± 3	1.27 ± 0.07 (18.6)	8.83 ± 0.05	109 ± 2	0.78 ± 0.05 (6.0)	10.84 ± 0.09	120 ± 3*	1.47 ± 0.08 (29.3)
	T207A	9.10 ± 0.04*	102 ± 1	0.95 ± 0.05 (8.9)	7.92 ± 0.05*	94 ± 2	0.53 ± 0.04 (3.4)	10.34 ± 0.09	102 ± 2	1.23 ± 0.07 (17.1)
	Q210A	9.63 ± 0.10	96 ± 3	0.93 ± 0.06 (8.5)	8.17 ± 0.07*	92 ± 4	0.85 ± 0.18 (7.1)	10.78 ± 0.10	99 ± 4	1.30 ± 0.11 (19.8)
	Q211A	9.14 ± 0.01*	98 ± 3	0.67 ± 0.04 (4.7)*	8.20 ± 0.08*	101 ± 4	0.45 ± 0.04 (2.8)*	10.51 ± 0.10	104 ± 4	1.13 ± 0.09 (13.5)
	H212A	9.62 ± 0.06	99 ± 3	1.10 ± 0.05 (13)	8.64 ± 0.07	100 ± 2	0.77 ± 0.04 (5.9)	10.45 ± 0.08	96 ± 2	1.06 ± 0.06 (11.6)
	Q213A	9.42 ± 0.11	100 ± 1	0.86 ± 0.07 (7.3)	8.70 ± 0.08	102 ± 3	0.48 ± 0.04 (3.1)*	10.38 ± 0.16	100 ± 5	0.96 ± 0.12 (9.0)
	W214A	8.95 ± 0.09*	97 ± 3	0.69 ± 0.04 (4.9)	8.24 ± 0.11*	92 ± 4	0.28 ± 0.04 (1.9)*	10.29 ± 0.13	104 ± 3	0.97 ± 0.10 (9.4)
	D215A	9.49 ± 0.12	105 ± 4	1.22 ± 0.38 (16.4)	8.40 ± 0.09	104 ± 3	0.42 ± 0.05 (2.6)*	10.02 ± 0.08*	107 ± 3	0.94 ± 0.06 (8.7)
	L217A	9.55 ± 0.14	97 ± 3	1.08 ± 0.08 (12.1)	8.66 ± 0.09	106 ± 4	0.66 ± 0.05 (4.6)	10.25 ± 0.05	112 ± 2	0.66 ± 0.04 (4.6)
	L218A	8.92 ± 0.07*	99 ± 2	0.87 ± 0.05 (7.4)	7.59 ± 0.10*	94 ± 4	0.41 ± 0.11 (2.6)*	9.51 ± 0.14*	100 ± 5	0.74 ± 0.09 (5.5)
	S219A	9.76 ± 0.09	101 ± 2	1.13 ± 0.06 (13.5)	8.55 ± 0.14	97 ± 7	0.57 ± 0.06 (3.7)	10.23 ± 0.09	108 ± 4	1.0 ± 0.07 (10.0)
	Y220A	9.47 ± 0.05	105 ± 2	1.09 ± 0.04 (12.4)	8.05 ± 0.10*	95 ± 7	0.50 ± 0.04 (3.1)*	10.53 ± 0.07	104 ± 3	1.33 ± 0.06 21.6)
	Q221A	9.85 ± 0.05	108 ± 1	1.38 ± 0.04 (23.9)	8.39 ± 0.10	92 ± 5	0.60 0.03 (4.0)	9.75 ± 0.08*	102 ± 2	0.75 ± 0.06 (5.6)
	D222A	9.94 ± 0.08*	99 ± 3	1.20 ± 0.06 (15.9)	8.48 ± 0.06	101 ± 2	0.57 ± 0.04 (3.7)	10.73 ± 0.08	103 ± 2	0.63 ± 0.09 (4.3)*

	S223A	*9.36 ± 0.07*	98 ± 2	0.92 ± 0.05 (8.4)	8.12 ± 0.07*	102 ± 2	0.51 ± 0.04 (3.2)	9.86 ± 0.09*	103 ± 3	0.63 ± 0.21 (4.3)*
**ECL3**	Wildtype	10.0 ± 0.09	100 ± 1	0.97 ± 0.07 (9.3)	8.43 ± 0.05	100 ± 3	0.91 ± 0.06 (8.0)	10.6 ± 0.10	100 ± 4	1.09 ± 0.11 (12.2)
	D372A	7.82 ± 0.15*	80 ± 9	0.50 ± 0.09 (3.2)*	6.63 ± 0.22*	75 ± 10	0.45 ± 0.18 (2.8)*	9.10 ± 0.11*	99 ± 5	0.99 ± 0.35 (9.8)
	E373A	6.97 ± 0.23*	64 ± 9*	0.23 ± 0.10 (1.7)*	6.92 ± 0.31*	40 ± 6*	-0.22 ± 0.11 (0.6)*	8.81 ± 0.24*	80 ± 13	0.39 ± 0.11 (2.5)*
	H374A	10.11 ± 0.08	102 ± 3	1.20 ± 0.09 (15.9)	8.49 ± 0.21	93 ± 6	0.94 ± 0.09 (8.7)	10.7 ± 0.17	101 ± 6	1.11 ± 0.13 (13.0)
	R376A	9.58 ± 0.10	101 ± 5	1.03 ± 0.16 (10.9)	8.29 ± 0.13	92 ± 4	0.72 ± 0.07 (5.2)	10.4 ± 0.14	97 ± 6	0.91 ± 0.10 (8.2)
	G377A	9.45 ± 0.11*	98 ± 4	0.83 ± 0.11 (6.7)	8.09 ± 0.16	88 ± 5	0.55 ± 0.06 (3.5)	10.5 ± 0.19	92 ± 9	0.83 ± 0.09 (6.8)
	T378A	10.6 ± 0.08*	101 ± 4	1.33 ± 0.09 (21.6)	8.44 ± 0.14	105 ± 4	0.92 ± 0.10 (8.4)	11.0 ± 0.16	108 ± 4	1.03 ± 0.11 (10.6)
	L379A	8.23 ± 0.15*	80 ± 6	0.45 ± 0.07 (2.8)*	6.95 ± 0.21*	62 ± 6*	0.13 ± 0.11 (1.4)*	10.0 ± 0.27	78 ± 13	0.45 ± 0.11 (2.8)*
	R380A	6.73 ± 0.13*	74 ± 8	0.58 ± 0.14 (3.8)*	ND*	ND*	ND*	8.33 ± 0.25*	69 ± 10*	0.36 ± 0.09 (2.3)*
	F381A	9.71 ± 0.18	74 ± 6	0.40 ± 0.05 (2.5)*	7.96 ± 0.16	80 ± 4	0.40 ± 0.10 (2.5)*	10.5 ± 0.24	88 ± 11	0.80 ± 0.10 (6.3)
	I382A	9.55 ± 0.10	96 ± 3	0.57 ± 0.05 (3.7)*	8.02 ± 0.12	86 ± 3	0.42 ± 0.06 (2.6)*	10.2 ± 0.15	86 ± 6	0.55 ± 0.07 (3.5)*
	K383A	6.89 ± 0.09*	87 ± 6	0.87 ± 0.22 (7.4)	7.22 ± 0.29*	32 ± 4*	-0.26 ± 0.12 (0.5)*	8.70 ± 0.24*	73 ± 14*	0.38 ± 0.10 (2.4)*
	L384A	8.65 ± 0.16*	91 ± 4	0.69 ± 0.08 (4.9)	7.34 ± 0.17*	92 ± 6	0.70 ± 0.12 (5.0)	10.0 ± 0.13	82 ± 2	0.50 ± 0.10 (3.1)*
	F385A	9.41 ± 0.11*	91 ± 6	0.44 ± 0.04 (2.8)*	7.84 ± 0.13	101 ± 5	0.54 ± 0.07 (3.5)*	10.3 ± 0.13	90 ± 6	0.64 ± 0.08 (4.3)*
	T386A	10.0 ± 0.13	101 ± 5	0.83 ± 0.07 (6.8)	8.56 ± 0.13	101 ± 4	0.91 ± 0.10 (8.2)	10.7 ± 0.15	98 ± 6	0.92 ± 0.11 (8.3)
	E387A	9.90 ± 0.09	108 ± 2	1.03 ± 0.08 (10.7)	7.23 ± 0.17*	61 ± 9*	0.14 ± 0.12 (1.4)*	10.3 ± 0.11	106 ± 4	0.98 ± 0.11 (9.5)

* Statistically significant at $p < 0.05$, one way ANOVA and Dunnett's post test in comparison to the wildtype response

Table S3. ***Related to Figure 5.*** **Effects of human GLP-1R ECL1 and ECL3 mutants on peptide agonist-induced intracellular calcium mobilisation.** Data were analyzed using an operational model of agonism as defined in eq 1 inSI. pEC_{50} values represent the negative logarithm of the concentration of agonist that produces half the maximal response. E_{max} are presented as a % of the WT response. Logτ values represents the intrinsic efficacy. All Logτ values were corrected to the cell surface expression data as determined by the ELISA ($Log\tau_c$) All values are expressed as mean ± S.E.M. of four to six individual experiments, performed in duplicate. Data were analyzed with one-way analysis of variance and Dunnett's post test.

		Agonist-mediated intracellular calcium mobilisation					
		GLP-1(7-36)NH_2			Exendin		
		pEC_{50}	E_{max}	$Log\tau_c$ (τ_c)	pEC_{50}	E_{max}	$Log\tau_c$ (τ_c)
	Wildtype	7.81 ± 0.09	100 ± 4	0.23 ± 0.08 (1.68)	7.94 ± 0.09	100 ± 4	0.43 ± 0.12 (2.69)
**ECL1**	L201A	ND *	ND*	ND*	6.76 ± 0.58*	17 ± 5*	-0.94 ± 0.33 (0.11)*
	K202A	6.86 ± 0.36*	40 ± 8*	-0.85 ± 0.20 (0.14)*	6.76 ± 0.25*	39 ± 5*	-0.52 ± 0.20 (0.30)*
	W203A	7.31 ± 0.41	51 ± 9*	-0.50 ± 0.20 (0.32)*	7.89 ± 0.19	74 ± 6*	0.03 ± 0.11 (1.07)
	M204A	ND*	ND*	ND*	ND*	ND*	ND*
	Y205A	7.26 ± 0.78	14 ± 3*	-0.99 ± 0.24 (0.10)*	6.84 ± 0.78*	22 ± 7*	-0.57 ± 0.28 (0.27)*
	S206A	7.92 ± 0.14	97 ± 5	0.17 ± 0.19 (1.45)	8.25 ± 0.18	82 ± 6	0.01 ± 0.10 (1.02)
	T207A	7.81 ± 0.33	22 ± 3*	-0.76 ± 0.27 (0.18)*	7.65 ± 0.26	13 ± 1*	-0.78 ± 0.21 (0.17)*
	Q210A	7.62 ± 0.16	113 ± 7	0.34 ± 0.19 (2.18)	7.78 ± 0.26	83 ± 10	0.22 ± 0.15 (1.66)
	Q211A	7.83 ± 0.11	135 ± 7*	0.69 ± 0.24 (3.89)	8.06 ± 0.16	123 ± 8*	0.65 ± 0.17 (4.47)
	H212A	7.87 ± 0.12	109 ± 5	0.48 ± 0.20 (3.03)	8.24 ± 0.12	87 ± 4	0.35 ± 0.12 (2.24)
	Q213A	7.39 ± 0.25	74 ± 8*	-0.20 ± 0.16 (0.64)	7.83 ± 0.28	85 ± 10	0.17 ± 0.12 (1.48)
	W214A	6.80 ± 0.14*	97 ± 8	0.44 ± 0.29 (2.77)	7.68 ± 0.19	90 ± 7	0.22 ± 011 (1.60)
	D215A	7.83 ± 0.10	125 ± 5*	1.00 ± 0.32 (9.90)*	8.08 ± 0.15	117 ± 7	0.70 ± 0.16 (5.01)
	L217A	7.40 ± 0.19	84 ± 7	-0.07 0.16 (0.86)	8.13 ± 0.20	78 ± 6	0.11 ± 0.09 (1.29)
	L218A	7.03 ± 0.12*	95 ± 9	0.58 ± 0.23 (3.81)	7.64 ± 0.14	85 ± 4	0.23 ± 0.12 (1.70)
	S219A	8.14 ± 0.14	101 ± 5	0.39 ± 0.21 (2.48)	8.26 ± 0.19	90 ± 6	0.29 ± 0.13 (1.95)
	Y220A	7.73 ± 0.13	118 ± 7	0.50 ± 0.21 (3.20)	7.36 ± 0.23	90 ± 9	0.37 ± 0.17 (2.34)
	Q221A	7.46 ± 0.12	94 ± 1	0.09 ± 0.17 (1.24)	7.96 ± 0.14	93 ± 5	0.39 ± 0.13 (2.45)
	D222A	7.84 ± 0.13	97 ± 5	0.22 ± 0.18 (1.64)	7.85 ± 0.10	101 ± 5	0.44 ± 0.13 (2.75)
	S223A	7.45 ± 0.14	74 ± 5*	-0.17 ± 0.16 (0.67)	7.99 ± 0.18	71 ± 5*	-0.03 ± 0.09 (0.93)

**ECL3**	Wildtype	7.87 ± 0.12	100 ± 6	0.23 ± 0.08 (1.68)	7.99 ± 0.08	100 ± 5	0.45 ± 0.10 (2.81)
	D372A	6.83 ± 0.18*	35 ± 2*	-0.50 ± 0.09 (0.31)*	6.79 ± 0.14*	18 ± 6*	-0.83 ± 0.10 (0.15)*
	E373A	ND*	ND*	ND*	ND*	ND*	ND*
	H374A	7.69 ± 0.08	97± 8	0.20 ± 0.09 (1.59)	7.98 ± 0.09	92 ± 7	0.34 ± 0.09 (2.19)
	R376A	7.88 ± 0.19	102 ± 10	0.24 ± 0.16 (1.73)	8.01 ± 0.10	95 ± 5	0.41 ± 0.09 (2.58)
	G377A	7.47 ± 0.19	89 ± 8	-0.02 ± 0.11 (0.95)	7.94 ± 0.12	86 ± 4	0.36 ± 0.01 (2.30)
	T378A	8.42 ± 0.09	179 ± 18*	1.23 ± 0.09 (171)*	8.55 ± 0.10	157 ± 14*	1.09 ± 0.14 (12.3)*
	L379A	ND*	ND*	ND*	6.61 ± 0.17*	38 ± 10*	-1.37 ± 0.12 (0.04)*
	R380A	ND*	ND*	ND*	ND*	ND*	ND*
	F381A	7.16 ± 0.15	83 ± 11	-0.40 ± 0.05 (0.39)	7.58 ± 0.16	110 ± 9	0.28 ± 0.08 (1.92)
	I382A	7.21 ± 0.10	79 ± 7	-0.27 ± 0.05 (0.54)	7.83 ± 0.10	85 ± 10	0.18 ± 0.16 (1.53)
	K383A	ND	ND	ND*	ND	ND	ND*
	L384A	7.47 ± 0.07	82 ± 6	-0.17 ± 0.08 (0.68)	6.89 ± 0.29*	75 ± 7*	-0.89 ± 0.11 (0.13)*
	F385A	7.81 ± 0.05	85 ± 4	0.02 ± 0.04 (1.06)	8.12 ± 0.08	99 ± 6	0.46 ± 0.10 (2.88)
	T386A	7.78 ± 0.12	95 ± 9	0.03 ± 0.06 (1.07)	7.29 ± 0.12	73 ± 9	-0.20 ± 0.08 (0.63)*
	E387A	7.69 ± 0.13	98 ± 10	0.23 ± 0.08 (1.70)	8.12 ± 0.14	106 ± 8	0.49 ± 0.10 (3.09)

* Statistically significant at $p < 0.05$, one way ANOVA and Dunnett's post test in comparison to the wildtype response

Table S4. ***Related to Figure 6.*** **Effects of human GLP-1R ECL1 and ECL3 mutants on peptide agonist-induced ERK/1/2 phosphorylation.** Data were analyzed using an operational model of agonism as defined in eq 1 in SI. pEC_{50} values represent the negative logarithm of the concentration of agonist that produces half the maximal response. E_{max} are presented as a % of the WT response. Logτ values represents the intrinsic efficacy. All Logτ values were corrected to the cell surface expression data as determined by the ELISA ($Log\tau_c$) All values are expressed as mean ± S.E.M. of four to six individual experiments, performed in duplicate. Data were analyzed with one-way analysis of variance and Dunnett's post test.

		Agonist-mediated ERK1/2 phosphorylation								
		GLP-1(7-36)NH_2			Oxyntomodulin			Exendin		
		pEC_{50}	E_{max}	$Log\tau_c$ (τ_c)	pEC_{50}	E_{max}	$Log\tau_c$ (τ_c)	pEC_{50}	E_{max}	$Log\tau_c$ (τ_c)
	Wildtype	8.33 ± 0.06	100 ± 2	0.42 ± 0.06 (2.62)	7.63 ± 0.08	100 ± 3	0.41 ± 0.05 (2.54)	8.48 ± 0.06	100 ± 2	0.44 ± 0.06 (2.74)
**ECL1**	L201A	7.37 ± 0.19*	82 ± 7	0.12 ± 0.11 (1.31)	7.93 ± 0.44	36 ± 7*	-0.44 ± 0.15 (0.36)*	7.94 ± 0.20	78 ± 6*	0.08 ± 0.05 (1.19)
	K202A	8.32 ± 0.11	111 ± 5	0.48 ± 0.18 (3.03)	7.62 ± 0.23	115 ± 11	0.59 ± 0.36 (3.90)	8.72 ± 0.17	129 ± 7*	0.89 ± 0.09 (7.72)
	W203A	7.79 ± 0.34	74 ± 10*	0.01 ± 0.06 (1.01)	6.87 ± 0.15	100 ± 9	0.08 ± 0.10 (1.20)	7.39 ± 0.40*	55 ± 11*	-0.31 ± 0.09 (0.49)*
	M204A	6.98 ± 0.12*	67 ± 5*	-0.08 ± 0.20 (0.84)*	7.35 ± 0.25	79 ± 9	0.01 ± 0.09 (1.26)	7.61 ± 0.18*	77 ± 6*	0.08 ± 0.12 (1.21)
	Y205A	7.87 ± 0.55	44 ± 9*	-0.14 ± 0.14 (0.72)*	6.15 ± 0.23*	49 ± 3*	-0.51 ± 0.22 (0.31)*	7.55 ± 0.14*	24 ± 5*	-0.68 ± 0.24 (0.22)*
	S206A	8.01 ± 0.18	83 ± 6	0.09 ± 0.07 (1.24)	7.68 ± 0.26	95 ± 11	0.32 ± 0.09 (2.10)	7.98 ± 0.18	72 ± 4*	-0.06 ± 0.07 (0.87)
	T207A	7.85 ± 0.39	58 ± 10*	0.03 ± 0.09 (1.07)	7.61 ± 0.30	53 ± 7*	0.08 ± 0.09 (1.21)	8.26 ± 0.33	36 ± 3*	-0.20 ± 0.10 (0.63)
	Q210A	8.71 ± 0.16	96 ± 5	0.54 ± 0.10 (3.43)	7.71 ± 0.13	123 ± 4	0.91 ± 0.38 (8.13)	8.22 ± 0.27	78 ± 8	0.17 ± 0.07 (1.48)
	Q211A	7.71 ± 0.23	117 ± 11	0.37 ± 0.09 (2.36)	7.89 ± 0.32	86 ± 11	0.33 ± 0.08 (2.14)	8.17 ± 0.17	95 ± 6	0.26 ± 0.07 (1.82)
	H212A	7.97 ± 0.24	109 ± 11	0.54 ± 0.07 (3.45)	7.60 ± 0.19	98 ± 8	0.53 ± 0.08 (3.40)	8.60 ± 0.17	105 ± 6	0.70 ± 0.06 (5.01)
	Q213A	7.79 ± 0.28	45 ± 3*	-0.37 ± 0.12 (0.42)*	7.25 ± 0.50	75 ± 21	0.01 ± 0.09 (1.01)	7.88 ± 0.21	80 ± 7	0.03 ± 0.08 (1.07)
	W214A	7.62 ± 0.21*	106 ± 10	0.40 ± 0.16 (2.52)	7.65 ± 0.26	85 ± 10	0.22 ± 0.07 (1.66)	8.02 ± 0.21	105 ± 9	0.32 ± 0.06 (2.07)
	D215A	7.75 ± 0.10	109 ± 5	0.55 ± 0.19 (3.52)	7.74 ± 0.25	127 ± 14	0.79 ± 0.10 (6.20)	8.65 ± 0.20	105 ± 7	0.63 ± 0.08 (4.23)
	L217A	7.37 ± 0.12*	108 ± 6	0.17 ± 0.07 (1.47)	7.43 ± 0.30	73 ± 10	0.06 ± 0.08 (1.16)	8.03 ± 0.19	64 ± 5*	-0.06 ± 0.09 (0.86)
	L218A	7.69 ± 0.20*	83 ± 7	0.12 ± 0.12 (1.31)	7.26 ± 0.22	80 ± 9	0.13 ± 0.12 (1.33)	7.85 ± 0.18	82 ± 6*	0.15 ± 0.11 (1.41)
	S219A	8.43 ± 0.13	125 ± 6	0.81 ± 0.11 (6.50)	7.86 ± 0.16	120 ± 8	0.76 ± 0.10 (5.82)	8.77 ± 0.14	91 ± 4	0.44 ± 0.07 (2.73)
	Y220A	8.13 ± 0.22	90 ± 8	0.38 ± 0.09 (2.39)	7.48 ± 0.22	77 ± 7	0.24 ± 0.09 (1.73)	8.12 ± 0.13	100 ± 5	0.45 ± 0.07 (2.82)
	Q221A	8.14 ± 0.27	86 ± 9	0.25 ± 0.08 (1.80)	7.81 ± 0.26	119 ± 8	0.77 ± 0.11 (5.90)	8.14 ± 0.23	89 ± 8	0.27 ± 0.07 (1.86)
	D222A	8.13 ± 0.25	108 ± 10	0.46 ± 0.08 (2.88)	7.39 ± 0.16	107 ± 7	0.41 ± 0.09 (2.56)	8.30 ± 0.20	104 ± 8	0.42 ± 0.06 (2.61)
	S223A	8.06 ± 0.16	141 ± 9	0.45 ± 0.11 (2.82)	7.76 ± 0.23	131 ± 12	0.84 ± 0.13 (6.91)	8.64 ± 0.25	100 ± 8	0.63 ± 0.22 (4.31)

**ECL3**	Wildtype	8.28 ± 0.11	100 ± 4	0.40 ± 0.03 (2.5)	7.57 ± 0.09	100 ± 4	0.49 ± 0.07 (3.09)	8.42 ± 0.14	100 ± 5	0.43 ± 0.19 (2.69)
	D372A	6.45 ± 0.20*	99 ± 14	-0.20 ± 0.17 (0.63)*	6.01 ± 0.08*	105 ± 21	-0.26 ± 0.11 (0.55)*	7.88 ± 0.40	60 ± 9*	-0.62 ± 0.17 (0.24)*
	E373A	6.78 ± 0.38*	59 ± 13*	-0.52 ± 0.03 (0.30)*	6.48 ± 0.25	104 ± 20	0.39 ± 0.16 (2.45)	7.27 ± 0.34	42 ± 6*	-1.05 ± 0.23 (0.09)*
	H374A	8.62 ± 0.12	88 ± 4	0.44 ± 0.09 (2.75)	7.71 ± 0.19	96 ± 8	0.30 ± 0.09 (2.0)	9.01 ± 0.18	64 ± 3*	-0.10 ± 0.22 (0.79)
	R376A	8.09 ± 0.17	77 ± 5	-0.18 ± 0.04 (0.66)*	6.99 ± 0.14	98 ± 8	0.04 ± 0.08 (1.10)	8.33 ± 0.24	79 ± 6	0.01 ± 0.16 (1.02)
	G377A	7.80 ± 0.15	87 ± 6	0.01 ± 0.02 (1.02)	7.05 ± 0.27	96 ± 13	1.53 ± 0.08 (33.9)*	8.78 ± 0.21	94 ± 6	0.58 ± 0.22 (3.80)
	T378A	8.77 ± 0.08	136 ± 4*	1.02 ± 0.24 (10.5)*	7.64 ± 0.13	192 ± 11*	-0.89 ± 0.14 (0.13)*	9.05 ± 0.15	136 ± 7*	1.33 ± 0.25 (21.4)*
	L379A	7.87 ± 0.22	52 ± 3*	-0.64 ± 0.05 (0.23)*	6.89 ± 0.23	92 ± 13	0.30 ± 0.24 (2.0)	7.70 ± 0.36	87 ± 7	0.06 ± 0.30 (1.15)
	R380A	7.15 ± 0.48*	25 ± 6*	-1.13 ± 0.04 (0.07)*	7.32 ± 0.45	30 ± 8*	1.20 ± 0.16 (15.8)*	6.94 ± 0.39*	69 ± 14	-0.34 ± 0.15 (0.46)*
	F381A	8.98 ± 0.12	115 ± 5	1.22 ± 0.44 (16.6)*	6.92 ± 0.20	105 ± 13	0.41 ± 0.12 (2.57)	8.50 ± 0.14	125 ± 5	0.92 ± 0.23 (8.32)
	I382A	8.71 ± 0.16	117 ± 6	0.98 ± 0.07 (9.55)*	7.54 ± 0.15	143 ± 9*	0.21 ± 0.12 (1.62)	8.51 ± 0.20	125 ± 8	0.92 ± 0.22 (8.32)
	K383A	6.68 ± 0.71*	38 ± 15*	-0.89 ± 0.11 (0.13)*	ND*	ND*	ND*	ND*	ND*	ND*
	L384A	7.06 ± 0.16*	99 ± 9	0.46 ± 0.13 (2.88)	6.63 ± 0.24	64 ± 10	0.03 ± 0.13 (1.07)	7.80 ± 0.11	108 ± 5	0.36 ± 0.18 (2.29)
	F385A	7.94 ± 0.17	91 ± 6	0.04 ± 0.04 (1.10)	7.24 ± 0.20	96 ± 8	-0.14 ± 0.10(0.72)*	8.47 ± 0.25	93 ± 8	0.31 ± 0.19 (2.04)
	T386A	7.76 ± 0.24	88 ± 9	-0.17 ± 0.11 (0.68)*	7.26 ± 0.11	93 ± 5	-0.21 ± 0.10 (0.62)*	8.34 ± 0.37	74 ± 9	-0.27 ± 0.15 (0.54)*
	E387A	7.79 ± 0.18	45 ± 7*	0.18 ± 0.03 (1.51)	6.96 ± 0.32	74 ± 15	-0.12 ± 0.11 (0.76)*	8.14 ± 0.21	72 ± 6	-0.31 ± 0.15 (0.49)*

* Statistically significant at $p < 0.05$, one way ANOVA and Dunnett's post test in comparison to the wildtype response

Table S5. *Related to Supplemental Experimental Precedures.* Major distance Constraints used in Modeller for the peptide docking.

Point A (GLP-1R)	GLP-1R location	Point B (GLP-1)	Evidence for constraint	constraint[c]	Reference
E125; C_δ	ECD	G^{35}; C_α	Bpa^{35} GLP-1 photoaffinity crosslink with E125.[a]	$r_{AB} \geq 20.0$ Å	Chen *et al.,* 2009
E133; C_γ	ECD	A^{24}; C_β	Bpa^{24} GLP-1 photoaffinity crosslink with E133.	$r_{AB} \leq 9.0$ Å	Chen *et al.,* 2009
L141;$C_{\delta 1}$	TM1	V^{16}; $C_{\gamma 1}$	Bpa^{16} GLP-1 photoaffinity crosslink with L141.	$r_{AB} \leq 9.0$ Å	Miller *et al.*, 2011
Y145;C_ξ	TM1	F^{12}; C_γ	Bpa^{12} GLP-1 photoaffinity crosslink with Y145.	$r_{AB} \leq 6.0$ Å	Chen *et al.*, 2010
W297;$C_{\eta 2}$	ECL2	L^{20}; C_γ	Bpa^{20} GLP-1 photoaffinity crosslink with W297.	$r_{AB} \leq 9.0$ Å	Miller *et al.*, 2011
K197;N_ξ	TM2	E^9; $O_{\varepsilon 2}$	Gain of function via reciprocal mutagenesis of residues between VIP and VPAC2R.[b]	$r_{AB} \leq 4.0$ Å	Solano et al., 2001
R380;$N_{\eta 2}$	ECL3	D^{15}; $O_{\varepsilon 2}$	Gain of function via reciprocal mutagenesis of residues between GLP-1 and GLP-1R.	$r_{AB} \leq 4.0$ Å	Moon *et al.*, 2015

a Constraint not used as the distance between $O\varepsilon 1$ of E133 and $C\alpha$ of G^{35} is 21.5 Å.
b The restraint gives similar results for E^9 with K197 and/or R190; if the constraint is used with both it is relaxed to $r_{AB} \leq 6.0$ Å
c The distances used were estimated from preliminary models in which BPA was replaced by tyrosine and a 6 Å constraint was used between O_η of the tyrosine (topologically equivalent to the reactive carbon atom of BPA) and a suitable point on the target residue.

Table S6. *Related to Supplemntal Experimental Procedures.* ECL1 Distance Constraints and other minor constraints used in Modeller for the molecular modelling. The ECL1 constraints were used to control the orientation of ECL1 in line with the variability data and were chosen by visual analysis. Torsional constraints were also used to constrain the backbone conformation of the last four residues of the Gs C-terminal peptide.

Point A (GLP-1R)	point A Location	Point B (GLP-1R)	Point B location	constraint
M204; C_{ε}	TM2	V26; $C_{\gamma 1}$	GLP-1	$r_{AB} \leq 8.0$
W214; $C_{\xi 2}$	ECL1	V16; $C_{\gamma 1}$	GLP-1	$r_{AB} \geq 12.0$
W214; $C_{\xi 2}$	ECL1	W203; $C_{\xi 3}$	TM2	$r_{AB} \geq 5.0$
H212; $C_{\varepsilon 1}$	ECL1	F12; C_{ξ}	GLP-1	$r_{AB} \geq 10.0$
H212; $C_{\varepsilon 1}$	ECL1	V16; C_{β}	GLP-1	$r_{AB} \geq 12.0$
H212; $C_{\varepsilon 1}$	ECL1	M13; C_{γ}	GLP-1	$r_{AB} \geq 12.0$
H212; $C_{\varepsilon 1}$	ECL1	L20; C_{γ}	GLP-1	$r_{AB} \geq 12.0$
Q213; C_{δ}	ECL1	H19; C_{β}	GLP-1	$r_{AB} \geq 10.0$
D215; C_{γ}	ECL1	V16; C_{β}	GLP-1	$r_{AB} \geq 12.0$
C226; C_{γ}	TM3	C296; C_{γ}	ECL2	disulfide
D15	GLP-1	L33	GLP-1	helix
T378	Helix 8	S392	Helix8	helix
V194	TM2	A209	TM3	helix
A221	TM3	F232	TM3	helix

Supplemental References

Baldwin, J.M., Schertler, G.F., and Unger, V.M. (1997). An alpha-carbon template for the transmembrane helices in the rhodopsin family of G-protein-coupled receptors. J Mol Biol *272*, 144-164.

Chen, Q., Pinon, D.I., Miller, L.J., and Dong, M.Q. (2009). Molecular Basis of Glucagon-like Peptide 1 Docking to Its Intact Receptor Studied with Carboxyl-terminal Photolabile Probes. J Biol Chem *284*, 34135-34144.

Chen, Q.A., Pinon, D.I., Miller, L.J., and Dong, M.Q. (2010). Spatial Approximations between Residues 6 and 12 in the Amino-terminal Region of Glucagon-like Peptide 1 and Its Receptor. A region critical for biological activity. J Biol Chem *285*, 24508-24518.

Dickson, C.J., Madej, B.D., Skjevik, A.A., Betz, R.M., Teigen, K., Gould, I.R., Walker, R.C. (2014). J Chem Theory Comput *10,* 865-879.

Eswar, N., Webb, B., Marti-Renom, M.S., Madhusudham, D.E., Shen, M.Y., Pieper, U., and Sali, A. (2007). Comparative Protein Structure Modeling with MODELLER. Curr Prot in Bioinformatics, 2.9.1-2.9.31.

Friesner, R.A., Banks, J.L., Murphy, R.B., Halgren, T.A., Klicic, J.J., Mainz, D.T., Repasky, M.P., Knoll, E.H., Shelley, M., Perry, J.K*., et al.* (2004). Glide: A new approach for rapid, accurate docking and scoring. 1. Method and assessment of docking accuracy. J Med Chem *47*, 1739-1749.

Harvey M.J, Giupponi G, and De Fabritiis G. (2009). ACEMD: Accelerating biomolecular dynamics in the microsecond time scale. J Chem Theory Comp *5,* 1632-1639.

Hoang, H.N., Song, K., Hill, T.A., Derksen, D.R., Edmonds, D.J., Kok, W.M., Limberakis, C., Liras, S., Loria, P.M., Mascitti, V*., et al.* (2015). Short Hydrophobic Peptides with Cyclic Constraints Are Potent Glucagon-like Peptide-1 Receptor (GLP-1R) Agonists. J Med Chem *58*, 4080-4085.

Hollenstein, K., Kean, J., Bortolato, A., Cheng, R.K.Y., Dore, A.S., Jazayeri, A., Cooke, R.M., Weir, M., and Marshall, F.H. (2013). Structure of class B GPCR corticotropin-releasing factor receptor 1. Nature *499*, 438-43.

Horn, F., Bettler, E., Oliveira, L., Campagne, F., Cohen, F.E., and Vriend, G. (2003). GPCRDB information system for G protein-coupled receptors 3. Nucleic Acids Res *31*, 294-297.

Hornak V, Abel R, Okur A, Strockbine B, Roitberg A, and Simmerling C. (2006). Comparison of multiple Amber force fields and development of improved protein backbone parameters. Proteins *65*, 712-725.

Humphrey, W., Dalke, A., and Schulten, K. (1996). VMD: visual molecular dynamics. J MolGraph *14*, 33-38.

Jacobson, M.P., Pincus, D.L., Rapp, C.S., Day, T.J., Honig, B., Shaw, D.E., and Friesner, R.A. (2004). A hierarchical approach to all-atom protein loop prediction. Proteins *55*, 351-367.

Kelley, L.A., Gardner, S.P., and Sutcliffe, M.J. (1997). An automated approach for defining core atoms and domains in an ensemble of NMR-derived protein structures. Protein Engineering *10*, 737-741.

Kelley, L.A., and Sutcliffe, M.J. (1997). OLDERADO: On-line database of ensemble representatives and domains. Protein Sci *6*, 2628-2630.

Koole, C., Wootten, D., Simms, J., Miller, L.J., Christopoulos, A., and Sexton, P.M. (2012). Second extracellular loop of human glucagon-like peptide-1 receptor (GLP-1R) has a critical role in GLP-1 peptide binding and receptor activation. J Biol Chem *287*, 3642-3658.

Koole, C., Wootten, D., Simms, J., Valant, C., Sridhar, R., Woodman, O.L., Miller, L.J., Summers, R.J., Christopoulos, A., and Sexton, P.M. (2010). Allosteric ligands of the glucagon-like peptide 1 receptor (GLP-1R) differentially modulate endogenous and exogenous peptide responses in a pathway-selective manner: implications for drug screening. Mol Pharmacol *78*, 456-465.

Miller, L.J., Chen, Q., Lam, P.C.H., Pinon, D.I., Sexton, P.M., Abagyan, R., and Dong, M.Q. (2011). Refinement of Glucagon-like Peptide 1 Docking to Its Intact Receptor Using Mid-region Photolabile Probes and Molecular Modeling. J Biol Chem *286*, 15895-15907.

Moon, M.J., Lee, Y.N., Park, S., Reyes-Alcaraz, A., Hwang, J.I., Millar, R.P., Choe, H., and Seong, J.Y. (2015). Ligand Binding Pocket Formed by Evolutionarily Conserved Residues in the Glucagon-like Peptide-1 (GLP-1) Receptor Core Domain. J Biol Chem *290*, 5696-5706.

Rasmussen, S.G.F., DeVree, B.T., Zou, Y.Z., Kruse, A.C., Chung, K.Y., Kobilka, T.S., Thian, F.S., Chae, P.S., Pardon, E., Calinski, D., *et al.* (2011). Crystal structure of the beta(2) adrenergic receptor-Gs protein complex. Nature *477*, 549-U311.

Shen, M.Y., and Sali, A. (2006). Statistical potential for assessment and prediction of protein structures. Protein Sci *15*, 2507-2524.

Siu, F.Y., He, M., de Graaf, C., Han, G.W., Yang, D., Zhang, Z., Zhou, C., Xu, Q., Wacker, D., Joseph, J.S., *et al.* (2013). Structure of the human glucagon class B G-protein-coupled receptor. Nature *499*, 444-449.

Solano, R.M., Langer, I., Perret, J., Vertongen, P., Juarranz, M.G., Robberecht, P., and Waelbroeck, M. (2001). Two basic residues of the h-VPAC(1) receptor second transmembrane helix are essential for ligand binding and signal transduction. J Biol Chem *276*, 1084-1088.

Taddese, B., Upton, G.J., Bailey, G.R., Jordan, S.R., Abdulla, N.Y., Reeves, P.J., and Reynolds, C.A. (2014). Do plants contain G protein-coupled receptors? Plant Physiol *164*, 287-307.

Tubert-Brohman, I., Sherman, W., Repasky, M., and Beuming, T. (2013). Improved Docking of Polypeptides with Glide. J Chem Inf Model *53*, 1689-1699.

Underwood, C.R., Garibay, P., Knudsen, L.B., Hastrup, S., Peters, G.H., Rudolph, R., and Reedtz-Runge, S. (2010). Crystal Structure of Glucagon-like Peptide-1 in Complex with the Extracellular Domain of the Glucagon-like Peptide-1 Receptor. J Biol Chem *285*, 723-730.

Vertongen, P., Solano, R.M., Perret, J., Langer, I., Robberecht, P., and Waelbroeck, M. (2001). Mutational analysis of the human vasoactive intestinal peptide receptor subtype VPAC(2): role of basic residues in the second transmembrane helix. Brit J Pharmacol *133*, 1249-1254.

Vohra, S., Taddese, B., Conner, A.C., Poyner, D.R., Hay, D.L., Barwell, J., Reeves, P.J., Upton, G.J., and Reynolds, C.A. (2013). Similarity between class A and class B G-protein-coupled receptors exemplified through calcitonin gene-related peptide receptor modelling and mutagenesis studies. J RSoc Interface *10*, 20120846.
